# Supplementary material for: Sugar-Induced Obesity and Insulin Resistance Are Uncoupled from Shortened Survival in Drosophila
Source: Cell Metab. 2020 Apr 7;31(4):710–725.e7. doi: 10.1016/j.cmet.2020.02.016 (PMC7156915; doi:10.1016/j.cmet.2020.02.016)
Supplement: Document S1. Figures S1–S7 and Tables S1–S5 [file mmc1.pdf]

Cell Metabolism, Volume *31*

## Supplemental Information

# Sugar-Induced Obesity and Insulin Resistance Are Uncoupled from Shortened Survival in *Drosophila*

**Esther van Dam, Lucie A.G. van Leeuwen, Eliano dos Santos, Joel James, Lena Best, Claudia Lennicke, Alec J. Vincent, Georgios Marinos, Andrea Foley, Marcela Buricova, Joao B. Mokochinski, Holger B. Kramer, Wolfgang Lieb, Matthias Laudes, Andre Franke, Christoph Kaleta, and Helena M. Cochemé**

# Supplemental Figure 1

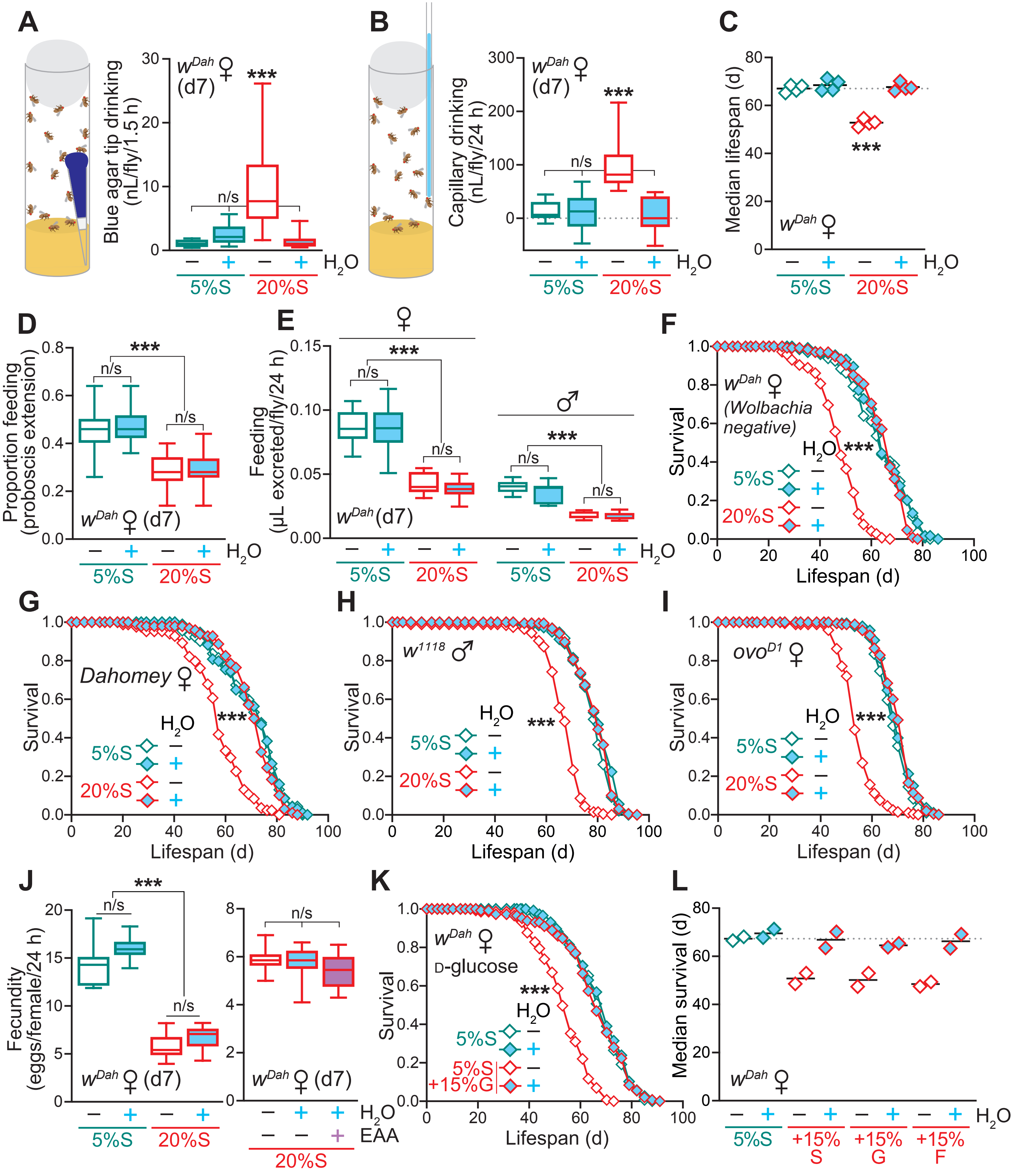

**Figure S1. A High-Sucrose Diet that Induces Thirst, Dehydration and Shortens Lifespan in Adult *Drosophila* Is Rescued by Water Supplementation (Related to Figure 1)**

(A and B) Drinking assays to quantify the thirst of WT ($w^{Dah}$) flies in response to a high-sugar diet. Females were pre-treated for 7 days on 5%S or 20%S ± $H_2O$. Data (n = 10 replicates per condition, each with 15 flies) are presented as box-and-whisker plots (min–max error bars), analyzed by one-way ANOVA with Tukey correction (n/s, $p > 0.05$; ***$p < 0.001$). (A) Blue dye drinking assay, with flies provided agar colored by blue dye and incubated for 1.5 h. The amount of dye ingested was then analyzed spectrophotometrically from fly homogenates to estimate the volume of water consumed from the agar tips. (B) Capillary drinking assay of flies incubated for 24 h. The drinking volume was corrected for an evaporation control, consisting of a capillary inserted into an empty vial (n = 8).

(C) Summary of median survival for n = 4 independent lifespan experiments of WT females on 5%S and 20%S ± $H_2O$. Data were analyzed by one-way ANOVA with Tukey correction (n/s, $p > 0.05$; ***$p < 0.001$).

(D and E) Feeding assays of WT flies pre-treated for 7 days on 5%S or 20%S ± $H_2O$. (D) Behavioral proboscis extension assay (n = 10 vials per condition, each with 5 females), with the proportion of flies feeding in each vial observed every ~2 min over a 90 min time course. Data are the average of n = 46 time points over the full experiment, presented as box-and-whisker plots (min–max error bars) and analyzed by one-way ANOVA with Tukey correction (n/s, $p > 0.05$; ***$p < 0.001$). (E) Quantification of fly feeding based on the excretion of food containing a blue tracer dye. WT females (n = 10 vials per condition, each with 15 flies) and males (n = 10 vials per condition, each with 20 flies) were pre-treated for 7 days on 5%S or 20%S ± $H_2O$, then exposed to the same food supplemented with 1% blue dye ± $H_2O$ for 24 h at 25ºC. Excreta from the vials were then solubilised, and the amount of blue dye was assayed spectrophotometrically. Data are presented as box-and-whisker plots (min–max error bars), analyzed by one-way ANOVA with Tukey correction (n/s, $p >0.05$; ***$p < 0.001$).

(F) Lifespan of $w^{Dah}$ females, negative for the endosymbiont *Wolbachia*, on 5%S and 20%S ± $H_2O$ (n ~ 150 flies per condition).

(G) Lifespan of *Dahomey* females on 5%S and 20%S ± $H_2O$ (n ~ 90–105 flies per condition).

(H) Lifespan of $w^{1118}$ males on 5%S and 20%S ± $H_2O$ (n ~ 200 flies per condition).

(I) Lifespan of sterile $ovo^{D1}$ mutant females on 5%S and 20%S ± $H_2O$ (n ~ 200 flies per condition).

(J) Fecundity (eggs laid/female/24 h) of WT females pre-treated for 7 days on 5%S or 20%S ± $H_2O$. Supplementation of the agar tips with a cocktail of essential amino acids (EAA) did not increase fecundity of females pre-treated on a 20%S diet for 7 days (n = 10 vials per condition, each with 15 flies). Data are presented as box-and-whisker plots (min–max error bars), analyzed by one-way ANOVA with Tukey correction (n/s, $p > 0.05$; ***$p < 0.001$).

(K) Lifespan of WT females ± $H_2O$ on a control diet (5%S) supplemented with 15% D-glucose (n ~ 150 flies per condition).

(L) Summary of median survival ± $H_2O$ for n = 2 independent lifespan experiments on excess 15% D-glucose or D-fructose, compared to the 20%S high-sucrose diet.

Statistical analysis of survival curves (F,G,H,I,K) was performed by log-rank test (n/s, $p > 0.05$; ***$p < 0.001$). See Table S3 for exact n numbers and p values.

# Supplemental Figure 2

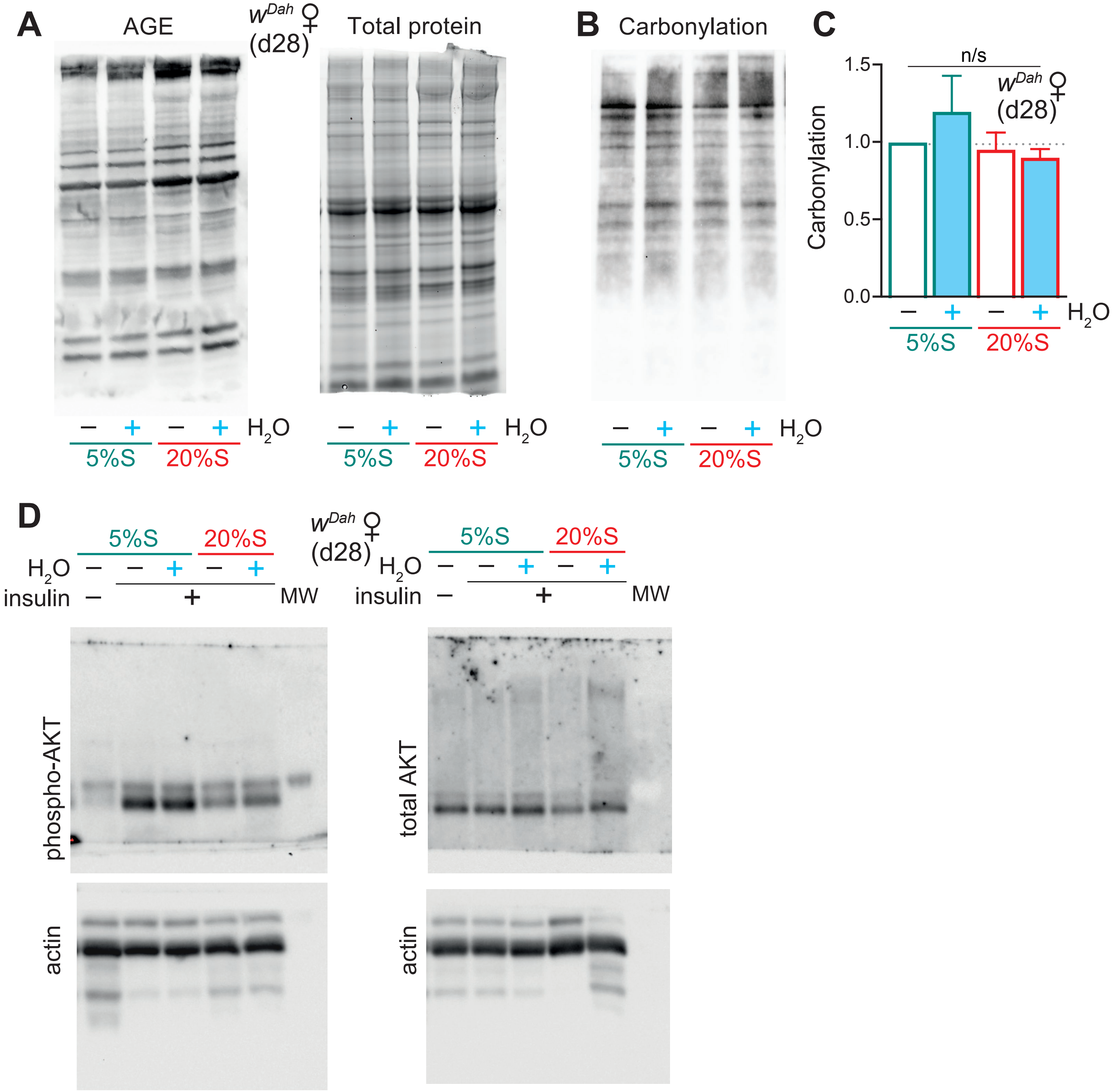

**Figure S2. Metabolic Effects of a High-Sucrose Diet and Water Supplementation in WT Adults (Related to Figure 2)**

(A) Glycation damage in whole body WT (*w*$^{Dah}$) females fed for 28 days on 5%S or 20%S ± $H_2O$. Full anti-AGE western blot and stain-free gel image of total protein relating to the quantification in Figure 2C.

(B and C) Protein carbonylation levels in whole body WT females fed for 28 days on 5%S or 20%S ± $H_2O$, assessed by oxyblot (B), and quantification by densitometry (C). Data are means + SEM of n = 3 experiments (with n = 5 flies per sample), analyzed by one-way ANOVA (n/s, $p > 0.05$).

(D) Insulin resistance of WT females fed for 28 days on 5%S or 20%S ± $H_2O$, assessed by AKT phosphorylation. Full western blots relating to Figures 2E,F. Dissected fat bodies (n = 5 per sample) were incubated ± insulin for 15 min, then homogenized in Laemmli buffer. Samples were run in parallel on two SDS-PAGE gels. The blots were cut horizontally, and the upper portion was probed against phospho-AKT and total AKT respectively (~65 kDa), while both lower portions were probed against actin as a loading control (~42 kDa).

# Supplemental Figure 3

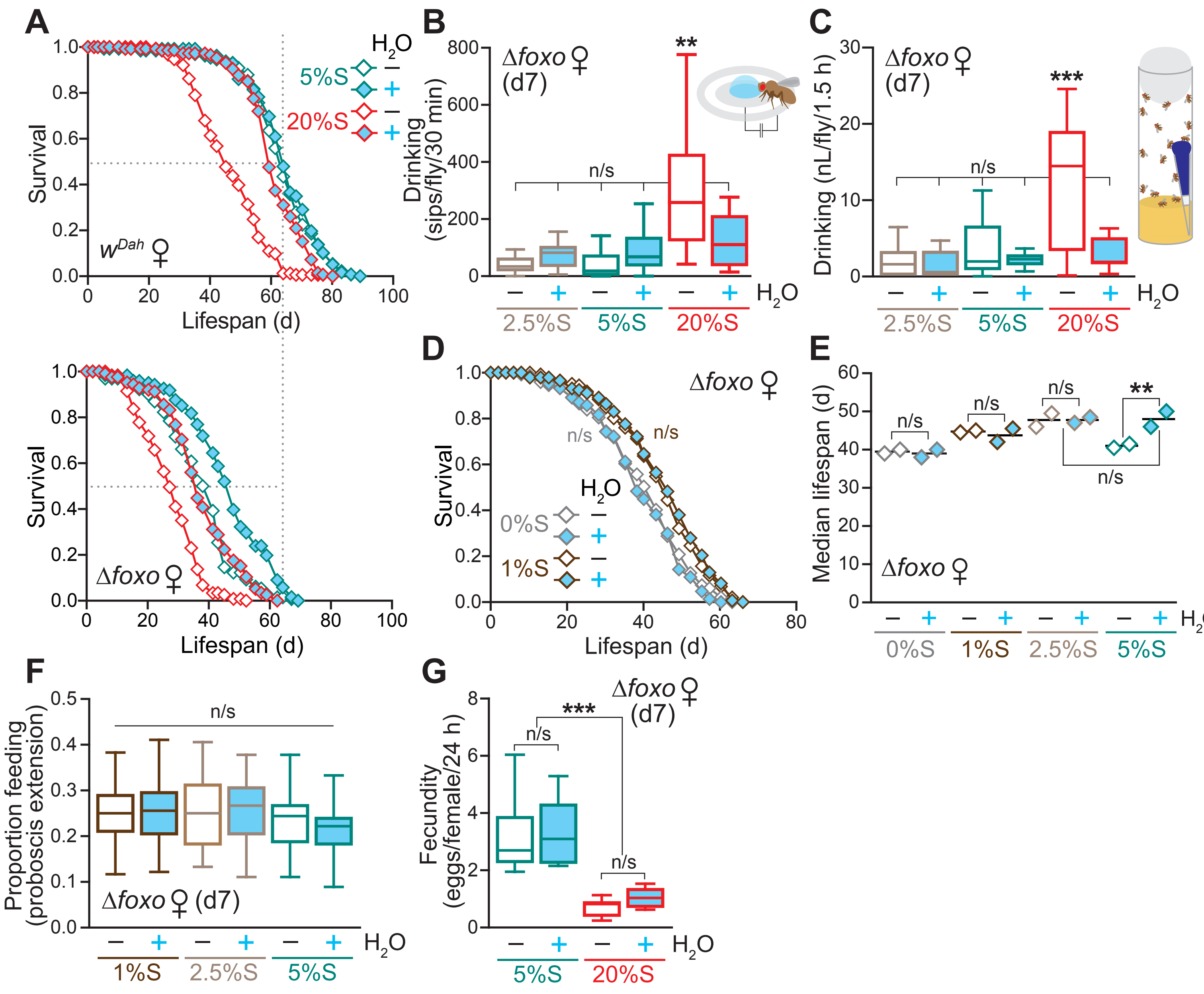

**Figure S3. Δ*foxo* Mutants are Hypersensitive to Dietary Sugar (Related to Figure 3)**

(A) Lifespan of $w^{Dah}$ and Δ*foxo* females on 5%S and 20%S ± $H_2O$ from a simultaneous experiment (n ~ 135–150 flies per condition). The grey dotted lines indicate median survival.

(B and C) Drinking assays of Δ*foxo* females pre-treated for 7 days on 2.5%S, 5%S or 20%S ± $H_2O$: (B) FlyPAD (n = 14–19 individual flies per condition), and (C) blue-dyed agar tips (n = 10 vials per condition, each with n = 15 flies). Box-and-whisker plots (min–max error bars), analyzed by one-way ANOVA with Tukey correction (n/s, $p > 0.05$; **$p < 0.01$; ***$p < 0.001$).

(D) Lifespan of Δ*foxo* females on 0%S and 1%S ± $H_2O$ (n ~ 150 flies per condition). Statistical analysis was performed by log-rank test (n/s, $p > 0.05$). See Table S3 for exact n numbers and p values.

(E) Summary of median survival for 2 independent Δ*foxo* lifespan experiments on 0%S, 1%S, 2.5%S and 5%S ± $H_2O$. Data were analyzed by one-way ANOVA (n/s, $p > 0.05$; **$p < 0.01$).

(F) Quantification of fly feeding based on behavioral proboscis extension. Δ*foxo* females (n = 10 vials per condition, each with 5 flies) were pre-treated for 7 days on 1%S, 2.5%S or 5%S ± $H_2O$. The proportion of flies feeding in each vial was observed every ~3 min over a 90 min time course. Data are the average of n = 31 time points over the full experiment, presented as box-and-whisker plots (min–max error bars) and analyzed by one-way ANOVA with Tukey correction (n/s, $p > 0.05$).

(G) Fecundity of Δ*foxo* females pre-treated for 7 days on 5%S or 20%S ± $H_2O$ (n = 10 vials per condition, each with 15 flies). Box-and-whisker plots (min–max error bars), analyzed by one-way ANOVA with Tukey correction (n/s, $p > 0.05$; ***$p < 0.001$).

# Supplemental Figure 4

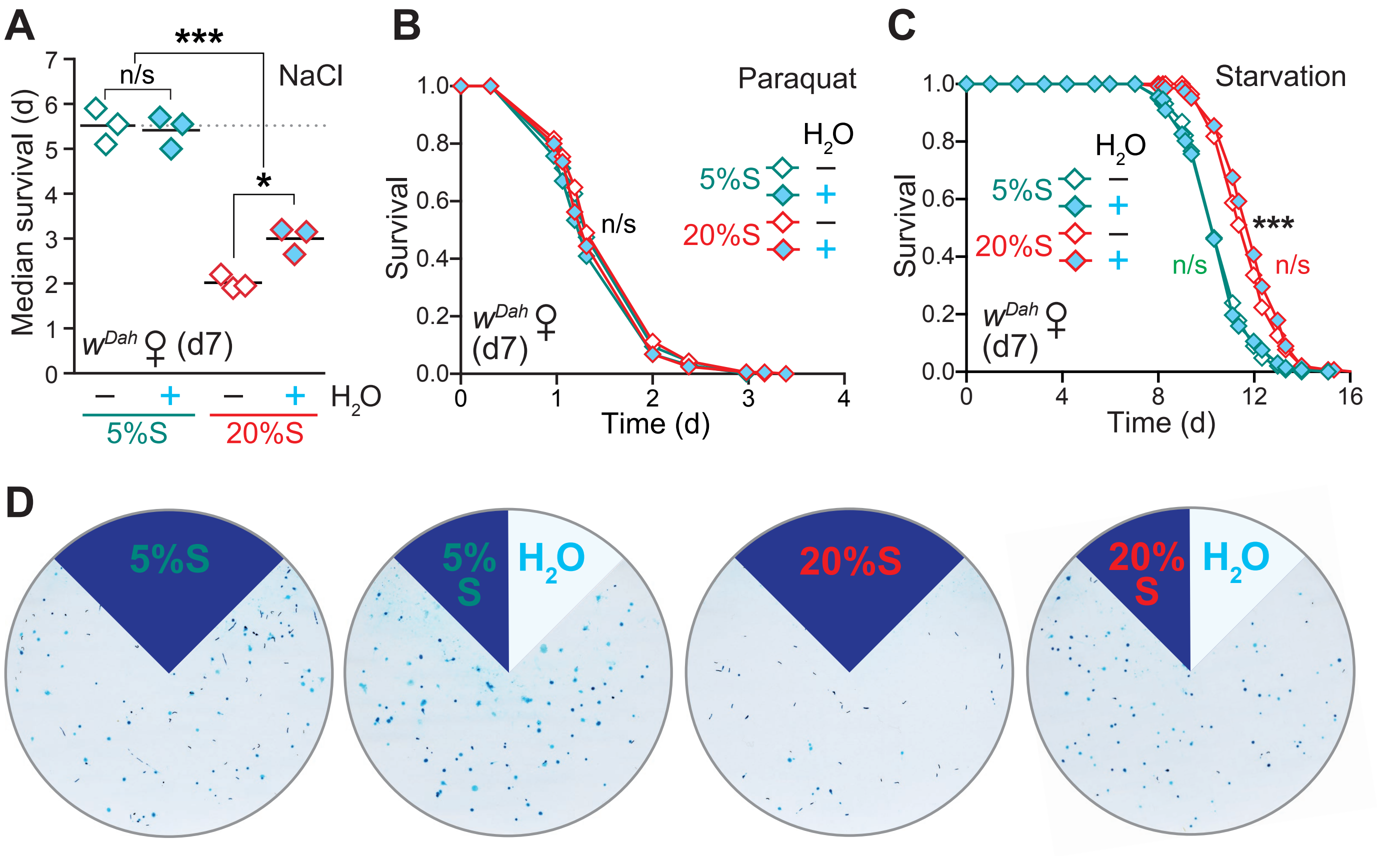

**Figure S4. Effects of a High-Sugar Diet on Stress Responses and Gut Physiology (Related to Figure 4)**

(A) Summary of median survival for 3 independent stress assays on 500 mM NaCl (see Figure 4B). Data were analyzed by one-way ANOVA with Tukey correction (n/s, $p > 0.05$; *$p < 0.05$; ***$p < 0.001$).

(B and C) Stress response of WT ($w^{Dah}$) females pre-treated for 7 days on 5%S or 20%S ± $H_2O$, then exposed to: oxidative stress consisting of 20 mM paraquat in 5%S food (B, n ~ 160 per condition), and starvation stress consisting of 1.5% agar (C, n ~ 150 flies per condition).

(D) Experimental setup and typical scans for the analysis of fly excreta. Food was supplemented with 2.5% w/v blue dye, while the agar for the water supplementation was undyed.

Statistical analysis of survival curves (B,C) was performed by log-rank test (n/s, $p > 0.05$; ***$p < 0.001$). See Table S3 for exact n numbers and p values.

# Supplemental Figure 5

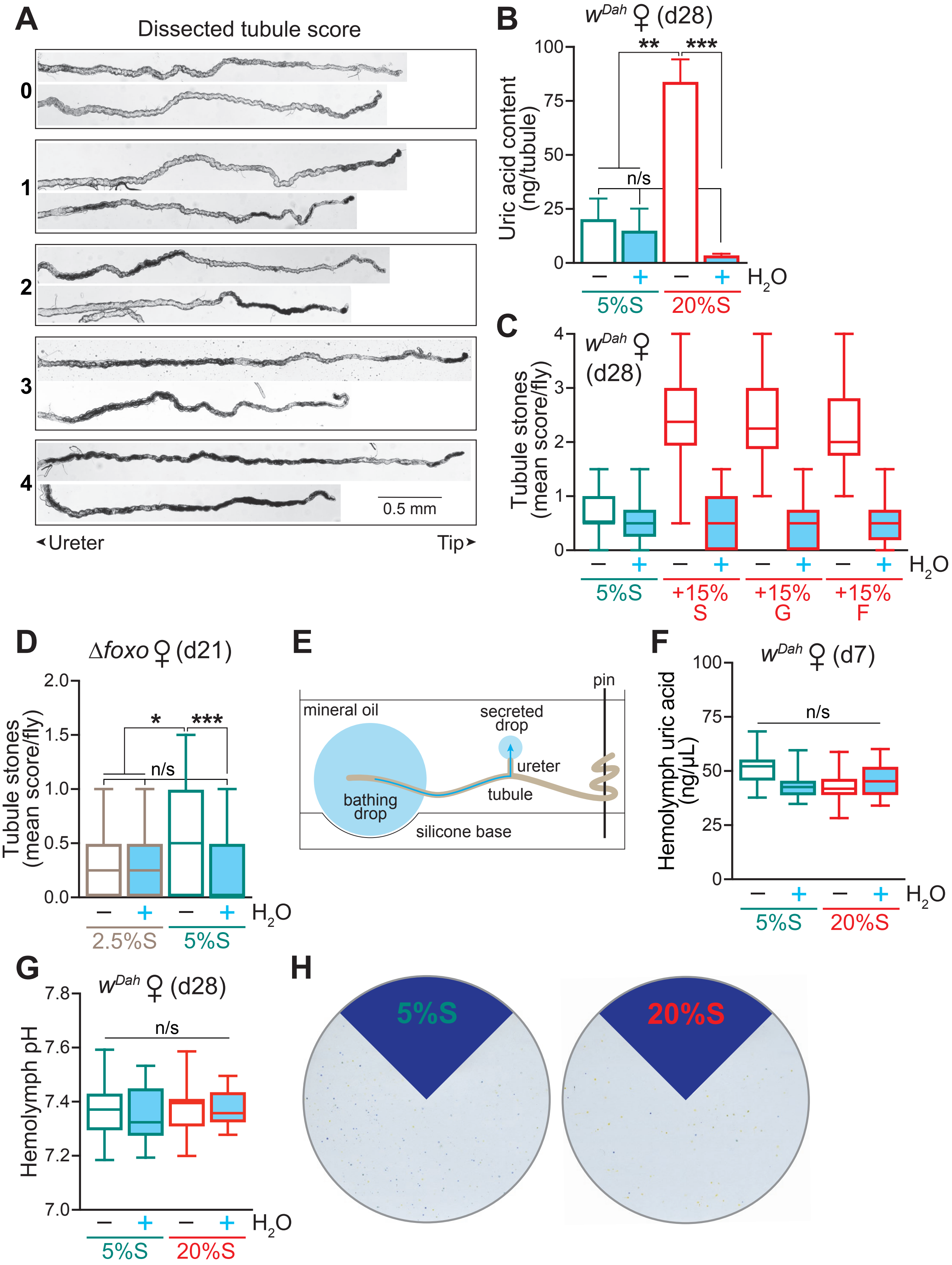

**Figure S5. A High-Sugar Diet Induces Uric Acid Deposition and Tubule Dysfunction (Related to Figure 5)**

(A) Scoring of the tubule stone phenotype based on light microscopy imaging of dissected tubules, arranged from the ureter (left) to the distal tip (right). Scale bar = 500 µm. Scoring: 0 = clear (~0%), 1 = mild (< 25%), 2 = moderate (25–50%), 3 = strong (50–75%), 4 = severe (> 75%).

(B) Uric acid content of dissected tubules from WT ($w^{Dah}$) females fed for 28 days on 5%S or 20%S ± $H_2O$. Data are means + SEM of n = 3 replicates (each with n = 6 pairs of tubules per sample), analyzed by one-way ANOVA with Tukey correction (n/s, $p > 0.05$; **$p < 0.01$; ***$p < 0.001$).

(C) Tubule phenotype scoring according to the scale in (A) of WT females maintained for 28 days ± $H_2O$ on excess 15% D-glucose or D-fructose, compared to the 5%S and 20%S sucrose diets (n = 100 flies per condition). Data are box-and-whisker plots (min–max error bars), analyzed by Kruskal-Wallis test with Dunn correction (n/s, $p > 0.05$; ***$p < 0.001$).

(D) Tubule phenotype scoring according to the scale in (A) of Δ*foxo* females maintained for 21 days on 2.5%S and 5%S ± $H_2O$. Box-and-whisker plots with min–max error bars (n = 57–70 flies per condition), analyzed by Kruskal-Wallis test with Dunn correction (n/s, $p > 0.05$; *$p < 0.05$; ***$p < 0.001$).

(E) Scheme of the tubule secretion assay. One arm of the tubule is bathed in a drop of saline with blue food dye, while the other is secured with a pin. Drops secreted from the ureter are measured over time.

(F) Uric acid levels in the hemolymph of flies fed for 7 days on 5%S or 20%S ± $H_2O$ (n = 12 replicates per condition, each with 12 flies per sample). Box-and-whisker plots (min–max error bars), analyzed by one-way ANOVA with Tukey correction (n/s, $p > 0.05$).

(G) Hemolymph pH of WT females treated for 28 days on 5%S or 20%S ± $H_2O$ (n = 9–17 samples, with 12 flies per sample). Box-and-whisker plots with min–max error bars, analyzed by one-way ANOVA with Tukey correction (n/s, $p > 0.05$).

(H) Typical scanned plates for the analysis of excreta pH using media supplemented with bromocresol purple (see Figure 5L).

# Supplemental Figure 6

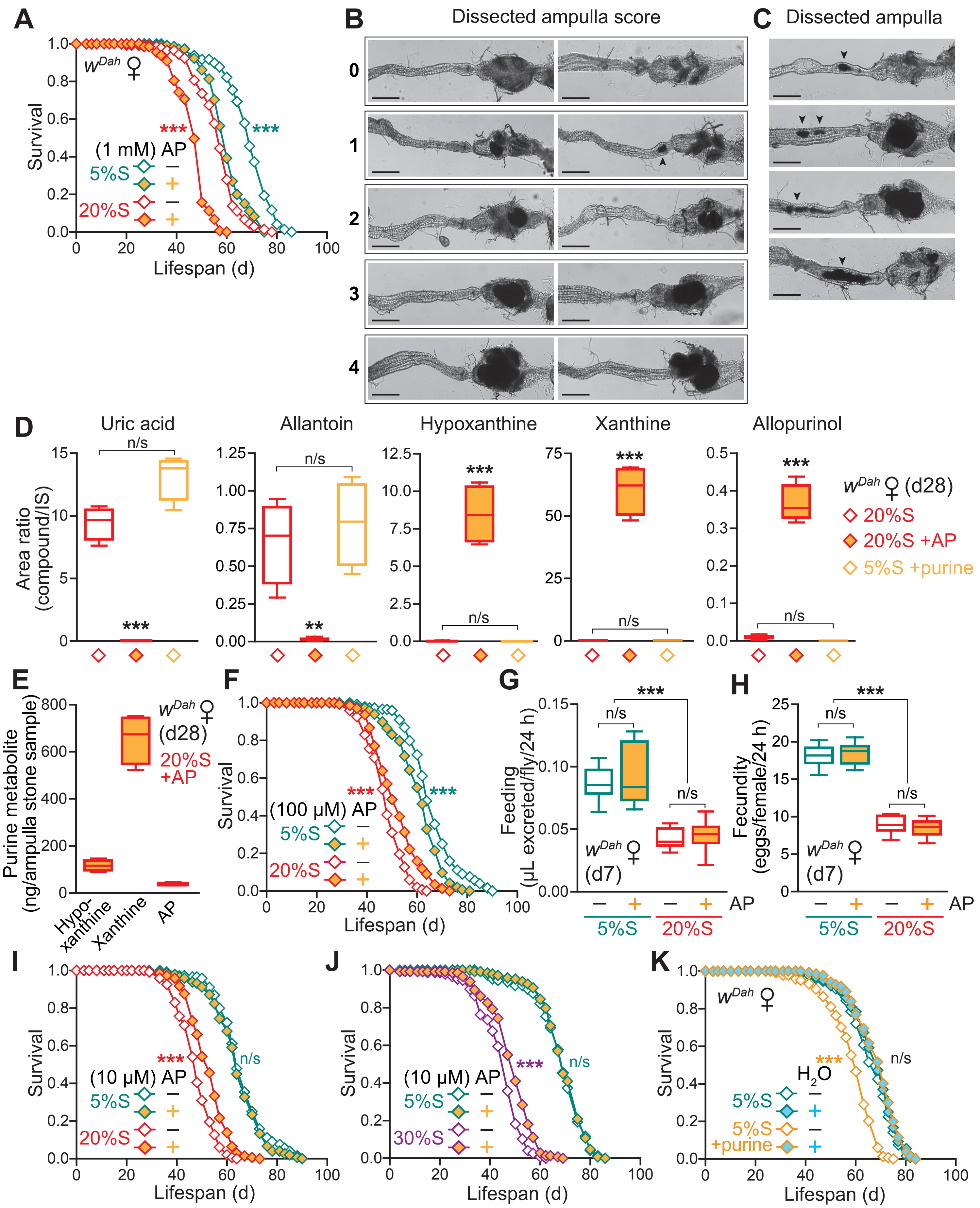

**Figure S6. Pharmacological Treatments and Dietary Interventions Targeting Purine Metabolism Impact on Lifespan (Related to Figure 6)**

(A) Chronic high allopurinol (1 mM AP) shortens the lifespan of WT ($w^{Dah}$) females on both the control (5%S ± AP) and high-sugar (20%S ± AP) diets (n ~ 135–150 flies per condition).

(B) Scoring of the rectal ampulla stone phenotype based on light microscopy imaging of dissected hindguts. Scale bar = 100 µm. Scoring: 0 = clear, 1 = mild, 2 = moderate, 3 = strong, 4 = severe.

(C) Light microscopy images showing concretions in the anterior hindgut (arrowheads), apparent upon allopurinol treatment.

(D) Metabolomics analysis of dissected rectal ampulla stones from WT females fed high-sugar (20%S), allopurinol-treated (20%S + AP, 1 mM), or high-purine (5%S + purine, 10 mM) diets for 28 days. n = 4 samples per condition, each with 25 units of stones as determined by the scoring scale in (B). Peak area ratios for the metabolite relative to the internal standard (IS) are presented as box-and-whisker plots (min–max error bars), analyzed by one-way ANOVA with Tukey correction (n/s, $p > 0.05$; ***$p < 0.001$).

(E) Quantification of hypoxanthine, xanthine and allopurinol levels in dissected rectal ampulla stones formed on the 20%S + AP condition (n = 4 per condition). Peak area data from (D) were converted to ng/sample based on calibration curves for each metabolite, and presented as box-and-whisker plots (min–max error bars).

(F) Independent biological repeat of the lifespan on 5%S and 20%S ± 100 µM AP in Figure 6C (n ~ 150 flies per condition).

(G and H) Dietary AP (100 µM) pre-treatment of WT females for 7 days does not affect feeding assessed by the blue dye excretion method (G) or fecundity (H) on each respective diet. Data (n = 10 replicates per condition, each with 15 flies per vial) are presented as box-and-whisker plots (min–max error bars), analyzed by one-way ANOVA with Tukey correction (n/s, $p > 0.05$; ***$p < 0.001$).

(I and J) Supplementation with 10 µM AP extends the lifespan of WT females on the 20%S (I) or 30%S (J) high-sugar diets, without affecting survival on control food 5%S ± AP (n ~ 150 flies per condition).

(K) Independent biological repeat of the lifespan on high-purine (10 mM) ± $H_2O$ in Figure 6F (n ~ 135–150 flies per condition).

Statistical analysis of survival curves (A,F,I,J,K) was performed by log-rank test (n/s, $p > 0.05$; ***$p < 0.001$). See Table S3 for exact n numbers and p values.

# Supplemental Figure 7

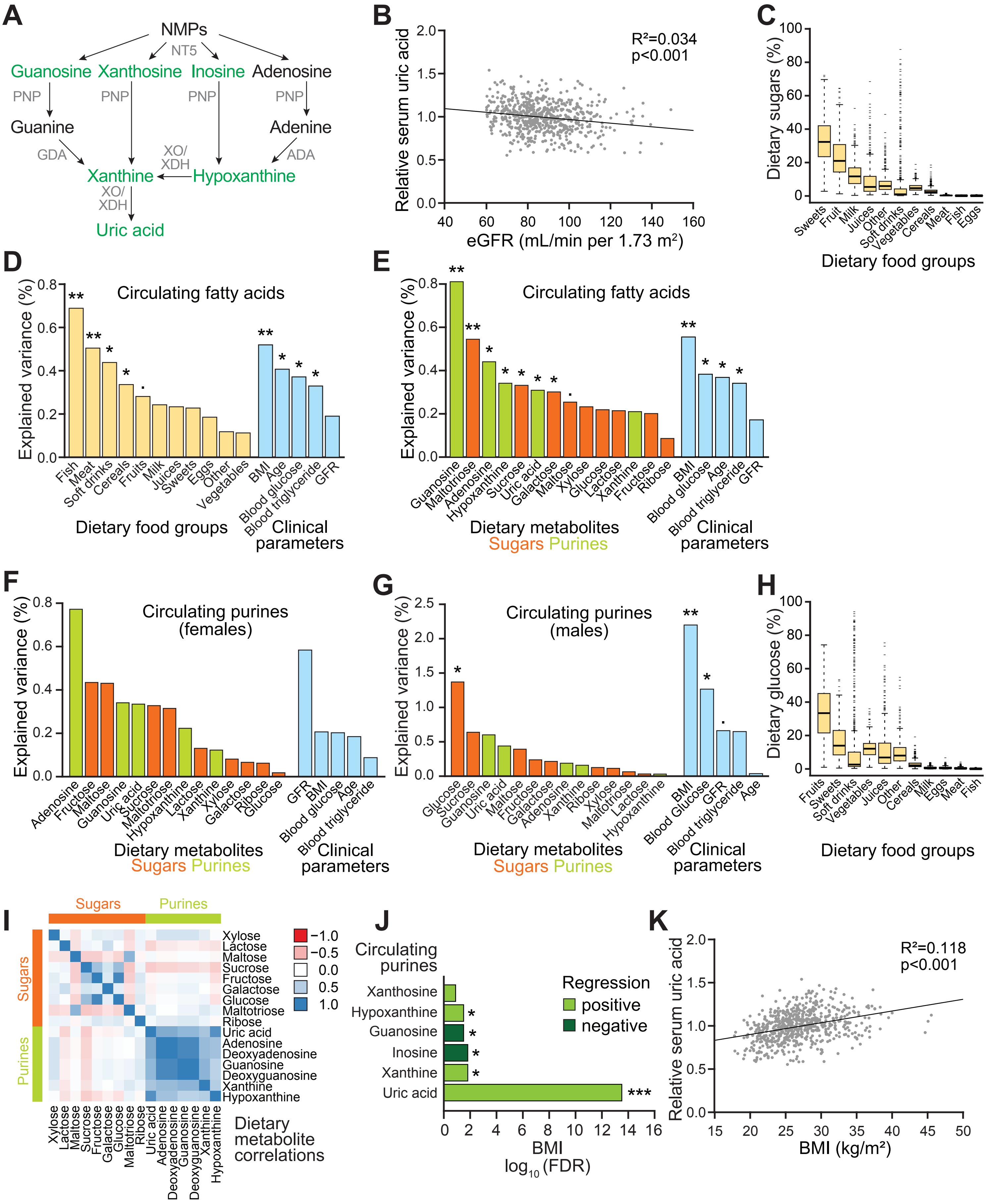

**Figure S7. Human Metabolomics Analysis Links Dietary Sugar Intake with Renal Function and Circulating Purine Levels (Related to Figure 7)**

(A) Scheme of the purine catabolism pathway, with metabolites measured by LC-MS from human blood serum color-coded in green. Nucleotide monophosphates (NMPs; i.e., GMP, XMP, IMP and AMP) are converted by various 5'-nucleotidases (NT5) into their respective nucleosides. The next steps are catalyzed by purine nucleoside phosphorylase (PNP), and guanine or adenine deaminase (GDA and ADA, respectively). The conversion of hypoxanthine to xanthine and finally to uric acid is catalyzed by the enzyme xanthine oxidase (XO)/xanthine dehydrogenase (XDH).

(B) Scatter plot of estimated glomerular filtration rate (eGFR) against circulating uric acid levels for the full cohort of n = 650 participants. A linear model predicting serum uric acid levels from eGFR, without any further independent terms, was overlaid as a regression line.

(C) Relative contribution of each food group to an individual's total dietary sugar intake (summed mono- and disaccharides). Data for the full cohort of n = 650 participants are displayed as box-and-whisker plots of the interquartile range (IQR), with the line corresponding to median and whiskers to 1.5x IQR.

(D-G) PERMANOVA analysis of circulating metabolites against dietary food groups or dietary metabolites (•p < 0.1; *p < 0.05; **p < 0.01). Clinical parameters are separated for visual clarity. Explained variance of dietary food groups (D) or dietary metabolites (E), color-coded in orange for sugars and green for purines, on the concentrations of circulating fatty acids. Analysis of variance of dietary metabolites against circulating purines in only female (F) or male (G) participants.

(H) Relative contribution of each food group to an individual's total dietary glucose intake. Fruit consumption accounted for more than 33.4% of total glucose intake in half of the cohort. Soft drinks showed a broad distribution as apparent from the dots corresponding to outliers, with some participants receiving up to 93.8% of their total glucose intake from soft drinks, while others hardly consuming any soft drinks at all (minimum = 0.33% of total glucose intake). Data for the full cohort of n = 650 participants are displayed as box-and-whisker plots of the interquartile range (IQR), with the line corresponding to median and whiskers to 1.5x IQR.

(I) Correlations between dietary metabolites imputed from food items in the food questionnaire. Spearman's rank-order correlation coefficient (rho; range –1 to 1) plotted as a heatmap (blue = positive, red = negative correlation). Darker colours are indicative of stronger correlations. Amongst dietary sugars, the only clear pattern is a very strong positive correlation between the dietary intake of glucose and fructose (*rho* > 0.937).

(J) Linear model of BMI predicting serum levels of individual purines (same analysis as for eGFR, shown in Figure 7B). Logarithmic FDRs are plotted as bars and color-coded for positive (light green) or negative (dark green) regressions.

(K) Scatter plot of BMI against circulating uric acid levels for the full cohort of n = 650 participants. A linear model predicting serum uric acid levels from BMI, without any further independent terms, was overlaid as a regression line.

## Table S1. *Drosophila* Diet Recipes (Related to STAR Methods)

### Sucrose Diets (per L of Media)

| Ingredient | Source | Low sucrose diets | | | Control diet | High sucrose diets | | |
|---|---|---|---|---|---|---|---|---|
| | | 0%S | 1%S | 2.5%S | 5%S | 20%S | 30%S | 40%S |
| Sucrose (% w/v) | Tate & Lyle, Granulated sugar | 0 g | 10 g | 25 g | 50 g | 200 g | 300 g | 400 g |
| Yeast (10% w/v) | MP Biomedicals, #903312 | 100 g | | | | | | |
| Agar (1.5% w/v) | Sigma, A7002 | 15 g | | | | | | |
| Nipagin | Sigma, H5501 | 30 mL of 10% w/v nipagin in 95% EtOH | | | | | | |
| Propionic acid (0.3% v/v) | Sigma, P1386 | 3 mL | | | | | | |
| $H_2O$ | | up to 1 L | | | | | | |

### Other High Sugar Diets (per L of Media)

| Ingredient | Source | 5%S | 5%S +15%G | 5%S +15%F |
|---|---|---|---|---|
| Sucrose (5% w/v) | Tate & Lyle, Granulated sugar | 50 g | | |
| D-Glucose (15% w/v) | Sigma G8270 | - | 150 g | - |
| D-Fructose (15% w/v) | Sigma F0127 | - | - | 150 g |
| Yeast (10% w/v) | MP Biomedicals, #903312 | 100 g | | |
| Agar (1.5% w/v) | Sigma, A7002 | 15 g | | |
| Nipagin | Sigma, H5501 | 30 mL of 10% w/v nipagin in 95% EtOH | | |
| Propionic acid (0.3% v/v) | Sigma, P1386 | 3 mL | | |
| $H_2O$ | | up to 1 L | | |

### Allopurinol (AP) Treatment (per L of Media)

| Ingredient | Source | 5%S ±AP | | | | 20%S ±AP | | | |
|---|---|---|---|---|---|---|---|---|---|
| | | - | +10 μM | +100 μM | +1 mM | - | +10 μM | +100 μM | +1 mM |
| Sucrose (% w/v) | Tate & Lyle, Granulated sugar | 50 g | | | | 200 g | | | |
| Yeast (10% w/v) | MP Biomedicals, #903312 | 100 g | | | | | | | |
| Agar (1.5% w/v) | Sigma, A7002 | 15 g | | | | | | | |
| Nipagin | Sigma, H5501 | 30 mL of 10% w/v nipagin in 95% EtOH | | | | | | | |
| Propionic acid (0.3% v/v) | Sigma, P1386 | 3 mL | | | | | | | |
| Allopurinol | Sigma, A8003 | - | 1.361 mg | 13.61 mg | 136.11 mg | - | 1.361 mg | 13.61 mg | 136.11 mg |
| $H_2O$ | | up to 1 L | | | | | | | |

### High Purine Diet (per L of Media)

| Ingredient | Source | 5%S | 5%S +10 mM purine |
|---|---|---|---|
| Sucrose (% w/v) | Tate & Lyle, Granulated sugar | 50 g | |
| Yeast (10% w/v) | MP Biomedicals, #903312 | 100 g | |
| Agar (1.5% w/v) | Sigma, A7002 | 15 g | |
| Nipagin | Sigma, H5501 | 30 mL of 10% w/v nipagin in 95% EtOH | |
| Propionic acid (0.3% v/v) | Sigma, P1386 | 3 mL | |
| Adenine (5 mM) | Sigma, A8626 | - | 675.65 mg |
| Guanine (5 mM) | Sigma, G11950 | - | 755.65 mg |
| $H_2O$ | | up to 1 L | |

# Table S2. *Drosophila* Survival Data (Related to Figures 1, 3, 4, 6)

**Fig. 1E**

| Experiment | Genotype | Condition | Flies per vial | # vials | Total flies (set-up) | Deaths | Censors | Total flies (actual) | Median (d) |
|---|---|---|---|---|---|---|---|---|---|
| Lifespan | $w^{Dah}$ females | 5%S –$H_2O$ | 15 | 10 | 150 | 129 | 9 | 138 | 66.3 |
| | | 5%S +$H_2O$ | 15 | 10 | 150 | 110 | 32 | 142 | 66.4 |
| | | 20%S –$H_2O$ | 15 | 10 | 150 | 141 | 7 | 148 | 51.0 |
| | | 20%S +$H_2O$ | 15 | 10 | 150 | 137 | 5 | 142 | 66.0 |

**P-value (Log-Rank test)**

| | *b* | *c* | *d* |
|---|---|---|---|
| *a* | 0.9347 | 4.4E-37 | 0.0585 |
| *b* | | 3.8E-34 | 0.0689 |
| *c* | | | 5.5E-31 |
| *d* | | | |

**Fig. 1F**

| Experiment | Genotype | Condition | Flies per vial | # vials | Total flies (set-up) | Deaths | Censors | Total flies (actual) | Median (d) |
|---|---|---|---|---|---|---|---|---|---|
| Lifespan | $w^{1118}$ females | 5%S –$H_2O$ | 15 | 11 | 165 | 157 | 9 | 166 | 75.2 |
| | | 5%S +$H_2O$ | 15 | 11 | 165 | 152 | 15 | 167 | 75.5 |
| | | 20%S –$H_2O$ | 15 | 11 | 165 | 162 | 4 | 166 | 59.5 |
| | | 20%S +$H_2O$ | 15 | 11 | 165 | 161 | 3 | 164 | 76.9 |

**P-value (Log-Rank test)**

| | *b* | *c* | *d* |
|---|---|---|---|
| *a* | 0.6184 | 3.1E-59 | 0.1403 |
| *b* | | 4.6E-58 | 0.0345 |
| *c* | | | 1.9E-63 |
| *d* | | | |

**Fig. 1G**

| Experiment | Genotype | Condition | Flies per vial | # vials | Total flies (set-up) | Deaths | Censors | Total flies (actual) | Median (d) |
|---|---|---|---|---|---|---|---|---|---|
| Lifespan (D-fructose) | $w^{Dah}$ females | 5%S –$H_2O$ | 15 | 10 | 150 | 145 | 2 | 147 | 68.1 |
| | | 5%S +$H_2O$ | 15 | 10 | 150 | 142 | 8 | 150 | 71.3 |
| | | 5%S+15% D-F –$H_2O$ | 15 | 10 | 150 | 144 | 2 | 146 | 49.4 |
| | | 5%S+15% D-F +$H_2O$ | 15 | 10 | 150 | 132 | 14 | 146 | 69.2 |

**P-value (Log-Rank test)**

| | *b* | *c* | *d* |
|---|---|---|---|
| *a* | 0.2099 | 1.3E-51 | 0.7720 |
| *b* | | 5.8E-52 | 0.2468 |
| *c* | | | 6.3E-35 |
| *d* | | | |

**Fig. 1H**

| Experiment | Genotype | Condition | Flies per vial | # vials | Total flies (set-up) | Deaths | Censors | Total flies (actual) | Median (d) |
|---|---|---|---|---|---|---|---|---|---|
| Lifespan | $w^{Dah}$ females | 5%S –$H_2O$ | 15 | 9 | 135 | 130 | 6 | 136 | 62.7 |
| | | 5%S +$H_2O$ | 15 | 9 | 135 | 123 | 11 | 134 | 63.7 |
| | | 30%S –$H_2O$ | 15 | 8 | 120 | 113 | 3 | 116 | 42.6 |
| | | 30%S +$H_2O$ | 15 | 8 | 120 | 118 | 0 | 118 | 60.7 |
| | | 40%S –$H_2O$ | 15 | 8 | 120 | 114 | 2 | 116 | 26.1 |
| | | 40%S +$H_2O$ | 15 | 8 | 120 | 115 | 3 | 118 | 60.2 |

**P-value (Log-Rank test)**

| | *b* | *c* | *d* | *e* | *f* |
|---|---|---|---|---|---|
| *a* | 0.6042 | 2.5E-37 | 0.0130 | 1.0E-49 | 0.0004 |
| *b* | | 5.1E-38 | 0.0014 | 1.6E-51 | 2.2E-05 |
| *c* | | | 2.3E-38 | 2.5E-11 | 1.7E-28 |
| *d* | | | | 9.1E-55 | 0.1575 |
| *e* | | | | | 7.3E-47 |
| *f* | | | | | |

**Fig. 3A**

| Experiment | Genotype | Condition | Flies per vial | # vials | Total flies (set-up) | Deaths | Censors | Total flies (actual) | Median (d) |
|---|---|---|---|---|---|---|---|---|---|
| Lifespan | Δ*foxo* females | 5%S –$H_2O$ | 15 | 13 | 195 | 176 | 15 | 191 | 39.5 |
| | | 5%S +$H_2O$ | 15 | 13 | 195 | 173 | 20 | 193 | 45.7 |
| | | 20%S –$H_2O$ | 15 | 13 | 195 | 180 | 14 | 194 | 26.7 |
| | | 20%S +$H_2O$ | 15 | 13 | 195 | 185 | 6 | 191 | 38.0 |

**P-value (Log-Rank test)**

| | *b* | *c* | *d* |
|---|---|---|---|
| *a* | 6.0E-07 | 2.8E-21 | 0.0329 |
| *b* | | 2.4E-40 | 5.9E-13 |
| *c* | | | 5.9E-17 |
| *d* | | | |

**Fig. 3D**

| Experiment | Genotype | Condition | Flies per vial | # vials | Total flies (set-up) | Deaths | Censors | Total flies (actual) | Median (d) |
|---|---|---|---|---|---|---|---|---|---|
| Lifespan | Δ*foxo* *females* | 2.5%S –$H_2O$ | 15 | 10 | 150 | 146 | 4 | 150 | 49.4 |
| | | 2.5%S +$H_2O$ | 15 | 10 | 150 | 136 | 9 | 145 | 46.9 |

**P-value (Log-Rank test)**

| | *b* |
|---|---|
| *a* | 0.4767 |
| *b* | |

**Fig. 4A**

| Experiment | Genotype | Condition (pre-treatment) | Flies per vial | # vials | Total flies (set-up) | Deaths | Censors | Total flies (actual) | Median (d) |
|---|---|---|---|---|---|---|---|---|---|
| Desiccation stress | $w^{Dah}$ females d28 | 5%S –$H_2O$ | 15 | 7 | 105 | 95 | 0 | 95 | 0.446 |
| | | 5%S +$H_2O$ | 15 | 7 | 105 | 103 | 0 | 103 | 0.418 |
| | | 20%S –$H_2O$ | 15 | 7 | 105 | 95 | 0 | 95 | 0.350 |
| | | 20%S +$H_2O$ | 15 | 7 | 105 | 94 | 0 | 94 | 0.417 |

**P-value (Log-Rank test)**

| | *b* | *c* | *d* |
|---|---|---|---|
| *a* | 0.0784 | 1.4E-09 | 0.1037 |
| *b* | | 8.5E-07 | 0.7036 |
| *c* | | | 3.3E-05 |
| *d* | | | |

**Fig. 4B**

| Experiment | Genotype | Condition (pre-treatment) | Flies per vial | # vials | Total flies (set-up) | Deaths | Censors | Total flies (actual) | Median (d) |
|---|---|---|---|---|---|---|---|---|---|
| Salt stress (500 mM NaCl) | $w^{Dah}$ females d7 | 5%S –$H_2O$ | 20 | 6 | 120 | 115 | 0 | 115 | 5.1 |
| | | 5%S +$H_2O$ | 20 | 6 | 120 | 113 | 0 | 113 | 5.0 |
| | | 20%S –$H_2O$ | 20 | 5 | 100 | 96 | 0 | 96 | 1.9 |
| | | 20%S +$H_2O$ | 20 | 6 | 120 | 118 | 0 | 118 | 2.7 |

**P-value (Log-Rank test)**

| | *b* | *c* | *d* |
|---|---|---|---|
| *a* | 0.5093 | 1.8E-27 | 6.5E-13 |
| *b* | | 3.5E-31 | 1.9E-12 |
| *c* | | | 3.7E-08 |
| *d* | | | |

**Fig. 6C**

| Experiment | Genotype | Condition | Flies per vial | # vials | Total flies (set-up) | Deaths | Censors | Total flies (actual) | Median (d) |
|---|---|---|---|---|---|---|---|---|---|
| Lifespan (100 μM allopurinol) | $w^{Dah}$ females | 5%S –AP | 15 | 10 | 150 | 140 | 6 | 146 | 64.7 |
| | | 5%S +AP | 15 | 10 | 150 | 147 | 0 | 147 | 61.0 |
| | | 20%S –AP | 15 | 10 | 150 | 149 | 0 | 149 | 47.5 |
| | | 20%S +AP | 15 | 10 | 150 | 145 | 3 | 148 | 51.4 |

**P-value (Log-Rank test)**

| | *b* | *c* | *d* |
|---|---|---|---|
| *a* | 1.4E-04 | 2.7E-40 | 5.3E-24 |
| *b* | | 7.0E-30 | 5.3E-13 |
| *c* | | | 5.7E-06 |
| *d* | | | |

**Fig. 6F**

| Experiment | Genotype | Condition | Flies per vial | # vials | Total flies (set-up) | Deaths | Censors | Total flies (actual) | Median (d) |
|---|---|---|---|---|---|---|---|---|---|
| Lifespan (10 mM purine) | $w^{Dah}$ females | 5%S –$H_2O$ | 15 | 8 | 120 | 118 | 1 | 119 | 72.9 |
| | | 5%S +$H_2O$ | 15 | 8 | 120 | 115 | 3 | 118 | 75.0 |
| | | 5%S+purine –$H_2O$ | 15 | 8 | 120 | 120 | 0 | 120 | 64.3 |
| | | 5%S+purine +$H_2O$ | 15 | 8 | 120 | 114 | 4 | 118 | 73.9 |

**P-value (Log-Rank test)**

| | *b* | *c* | *d* |
|---|---|---|---|
| *a* | 0.1633 | 4.5E-13 | 0.6151 |
| *b* | | 6.9E-18 | 0.4106 |
| *c* | | | 5.8E-14 |
| *d* | | | |

# Table S3. *Drosophila* Survival Data (Related to Figures 1, 3, 4, 6 and S1, S3, S4, S6)

**Fig. S1F**

| Experiment | Genotype | Condition | Flies per vial | # vials | Total flies (set-up) | Deaths | Censors | Total flies (actual) | Median (d) |
|---|---|---|---|---|---|---|---|---|---|
| Lifespan | $w^{Dah}$ | 5%S $-H_2O$ | 15 | 10 | 150 | 144 | 4 | 148 | 62.8 |
| | *Wolbachia* | 5%S $+H_2O$ | 15 | 10 | 150 | 127 | 14 | 141 | 63.3 |
| | negative | 20%S $-H_2O$ | 15 | 10 | 150 | 145 | 2 | 147 | 47.0 |
| | females | 20%S $+H_2O$ | 15 | 10 | 150 | 129 | 8 | 137 | 65.2 |

**P-value (Log-Rank test)**

| | *b* | *c* | *d* |
|---|---|---|---|
| *a* | 0.3795 | 8.0E-36 | 0.1177 |
| *b* | | 1.1E-40 | 0.0827 |
| *c* | | | 4.4E-43 |
| *d* | | | |

**Fig. S1G**

| Experiment | Genotype | Condition | Flies per vial | # vials | Total flies (set-up) | Deaths | Censors | Total flies (actual) | Median (d) |
|---|---|---|---|---|---|---|---|---|---|
| Lifespan | *Dahomey* | 5%S $-H_2O$ | 15 | 7 | 105 | 92 | 0 | 92 | 72.8 |
| | females | 5%S $+H_2O$ | 15 | 7 | 105 | 94 | 3 | 97 | 73.0 |
| | | 20%S $-H_2O$ | 15 | 6 | 90 | 84 | 1 | 85 | 56.3 |
| | | 20%S $+H_2O$ | 15 | 7 | 105 | 98 | 0 | 98 | 71.2 |

**P-value (Log-Rank test)**

| | *b* | *c* | *d* |
|---|---|---|---|
| *a* | 0.7744 | 2.1E-14 | 0.18286 |
| *b* | | 1.8E-14 | 0.12491 |
| *c* | | | 7.4E-16 |
| *d* | | | |

**Fig. S1H**

| Experiment | Genotype | Condition | Flies per vial | # vials | Total flies (set-up) | Deaths | Censors | Total flies (actual) | Median (d) |
|---|---|---|---|---|---|---|---|---|---|
| Lifespan | $w^{1118}$ | 5%S $-H_2O$ | 20 | 10 | 200 | 185 | 13 | 198 | 78.3 |
| | males | 5%S $+H_2O$ | 20 | 10 | 200 | 179 | 12 | 191 | 79.3 |
| | | 20%S $-H_2O$ | 20 | 10 | 200 | 198 | 2 | 200 | 66.7 |
| | | 20%S $+H_2O$ | 20 | 10 | 200 | 187 | 9 | 196 | 79.1 |

**P-value (Log-Rank test)**

| | *b* | *c* | *d* |
|---|---|---|---|
| *a* | 0.0133 | 5.8E-48 | 0.1633 |
| *b* | | 1.1E-45 | 0.1988 |
| *c* | | | 2.9E-48 |
| *d* | | | |

**Fig. S1I**

| Experiment | Genotype | Condition | Flies per vial | # vials | Total flies (set-up) | Deaths | Censors | Total flies (actual) | Median (d) |
|---|---|---|---|---|---|---|---|---|---|
| Lifespan | $ovo^{D1}$ | 5%S $-H_2O$ | 20 | 10 | 200 | 193 | 6 | 199 | 67.6 |
| | females | 5%S $+H_2O$ | 20 | 10 | 200 | 193 | 4 | 197 | 68.3 |
| | | 20%S $-H_2O$ | 20 | 10 | 200 | 197 | 1 | 198 | 52.6 |
| | | 20%S $+H_2O$ | 20 | 10 | 200 | 193 | 2 | 195 | 70.0 |

**P-value (Log-Rank test)**

| | *b* | *c* | *d* |
|---|---|---|---|
| *a* | 0.0531 | 5.3E-58 | 0.0109 |
| *b* | | 2.3E-64 | 0.5894 |
| *c* | | | 4.7E-65 |
| *d* | | | |

**Fig. S1K**

| Experiment | Genotype | Condition | Flies per vial | # vials | Total flies (set-up) | Deaths | Censors | Total flies (actual) | Median (d) |
|---|---|---|---|---|---|---|---|---|---|
| Lifespan | $w^{Dah}$ | 5%S $-H_2O$ | 15 | 10 | 150 | 136 | 3 | 139 | 66.5 |
| (D-glucose) | females | 5%S $+H_2O$ | 15 | 10 | 150 | 146 | 1 | 147 | 67.8 |
| | | 5%S+15% D-G $-H_2O$ | 15 | 10 | 150 | 145 | 4 | 149 | 53.0 |
| | | 5%S+15% D-G $+H_2O$ | 15 | 10 | 150 | 143 | 5 | 148 | 65.4 |

**P-value (Log-Rank test)**

| | *b* | *c* | *d* |
|---|---|---|---|
| *a* | 0.4259 | 1.3E-25 | 0.9382 |
| *b* | | 3.6E-28 | 0.4137 |
| *c* | | | 5.5E-23 |
| *d* | | | |

**Fig. S3A**

| Experiment | Genotype | Condition | Flies per vial | # vials | Total flies (set-up) | Deaths | Censors | Total flies (actual) | Median (d) |
|---|---|---|---|---|---|---|---|---|---|
| Lifespan | $w^{Dah}$ | 5%S $-H_2O$ | 15 | 9 | 135 | 130 | 6 | 136 | 62.7 |
| | females | 5%S $+H_2O$ | 15 | 9 | 135 | 123 | 11 | 134 | 63.7 |
| | | 20%S $-H_2O$ | 15 | 10 | 150 | 153 | 1 | 154 | 44.5 |
| | | 20%S $+H_2O$ | 15 | 10 | 150 | 144 | 5 | 149 | 59.0 |
| Lifespan | $\Delta$*foxo* | 5%S $-H_2O$ | 15 | 9 | 135 | 130 | 6 | 136 | 38.1 |
| | females | 5%S $+H_2O$ | 15 | 9 | 135 | 121 | 10 | 131 | 45.6 |
| | | 20%S $-H_2O$ | 15 | 9 | 135 | 117 | 21 | 138 | 27.1 |
| | | 20%S $+H_2O$ | 15 | 9 | 135 | 124 | 13 | 137 | 35.4 |

**P-value (Log-Rank test)**

| | *b* | *c* | *d* |
|---|---|---|---|
| *a* | 0.6042 | 1.1E-29 | 0.001 |
| *b* | | 3.9E-31 | 6.6E-05 |
| *c* | | | 6.9E-24 |
| *d* | | | |

| | *f* | *g* | *h* |
|---|---|---|---|
| *e* | 2.0E-08 | 3.2E-14 | 0.8824 |
| *f* | | 2.5E-31 | 7.6E-09 |
| *g* | | | 2.1E-13 |
| *h* | | | |

**Fig. S3D**

| Experiment | Genotype | Condition | Flies per vial | # vials | Total flies (set-up) | Deaths | Censors | Total flies (actual) | Median (d) |
|---|---|---|---|---|---|---|---|---|---|
| Lifespan | $\Delta$*foxo* | 0%S $-H_2O$ | 15 | 10 | 150 | 149 | 0 | 149 | 40.2 |
| | females | 0%S $+H_2O$ | 15 | 10 | 150 | 147 | 3 | 150 | 37.9 |
| | | 1%S $-H_2O$ | 15 | 10 | 150 | 146 | 2 | 148 | 44.7 |
| | | 1%S $+H_2O$ | 15 | 10 | 150 | 151 | 1 | 152 | 45.7 |

**P-value (Log-Rank test)**

| | *b* | *c* | *d* |
|---|---|---|---|
| *a* | 0.3017 | 1.0E-03 | 8.2E-06 |
| *b* | | 1.5E-05 | 3.5E-08 |
| *c* | | | 0.2079 |
| *d* | | | |

**Fig. S4B**

| Experiment | Genotype | Condition (pre-treatment) | Flies per vial | # vials | Total flies (set-up) | Deaths | Censors | Total flies (actual) | Median (d) |
|---|---|---|---|---|---|---|---|---|---|
| Oxidative stress | $w^{Dah}$ | 5%S $-H_2O$ | 20 | 8 | 160 | 158 | 0 | 158 | 1.29 |
| (20 mM paraquat) | females d7 | 5%S $+H_2O$ | 20 | 8 | 160 | 161 | 0 | 161 | 1.22 |
| | | 20%S $-H_2O$ | 20 | 8 | 160 | 159 | 0 | 159 | 1.30 |
| | | 20%S $+H_2O$ | 20 | 8 | 160 | 160 | 0 | 160 | 1.25 |

**P-value (Log-Rank test)**

| | *b* | *c* | *d* |
|---|---|---|---|
| *a* | 0.1702 | 0.6081 | 0.4543 |
| *b* | | 0.0578 | 0.5040 |
| *c* | | | 0.2292 |
| *d* | | | |

**Fig. S4C**

| Experiment | Genotype | Condition (pre-treatment) | Flies per vial | # vials | Total flies (set-up) | Deaths | Censors | Total flies (actual) | Median (d) |
|---|---|---|---|---|---|---|---|---|---|
| Starvation stress | $w^{Dah}$ | 5%S $-H_2O$ | 15 | 10 | 150 | 146 | 0 | 146 | 10.2 |
| (1.5% agar) | females d7 | 5%S $+H_2O$ | 15 | 10 | 150 | 132 | 0 | 132 | 10.2 |
| | | 20%S $-H_2O$ | 15 | 10 | 150 | 143 | 0 | 143 | 11.4 |
| | | 20%S $+H_2O$ | 15 | 10 | 150 | 145 | 0 | 145 | 11.7 |

**P-value (Log-Rank test)**

| | *b* | *c* | *d* |
|---|---|---|---|
| *a* | 0.8907 | 4.3E-13 | 3.2E-17 |
| *b* | | 3.4E-11 | 8.5E-15 |
| *c* | | | 0.1624 |
| *d* | | | |

# Table S3. *Drosophila* Survival Data (Related to Figures 1, 3, 4, 6 and S1, S3, S4, S6) - *continued*

**Fig. S6A**

| Experiment | Genotype | Condition | Flies per vial | # vials | Total flies (set-up) | Deaths | Censors | Total flies (actual) | Median (d) |
|---|---|---|---|---|---|---|---|---|---|
| Lifespan (1 mM allopurinol) | $w^{Dah}$ females | 5%S –AP | 15 | 10 | 150 | 146 | 1 | 147 | 68.4 |
| | | 5%S +AP | 15 | 10 | 150 | 145 | 1 | 146 | 58.2 |
| | | 20%S –AP | 15 | 10 | 150 | 143 | 6 | 149 | 56.3 |
| | | 20%S +AP | 15 | 9 | 135 | 133 | 2 | 135 | 46.9 |

**P-value (Log-Rank test)**

| | *b* | *c* | *d* |
|---|---|---|---|
| *a* | 4.8E-19 | 2.4E-28 | 1.5E-55 |
| *b* | | 0.0044 | 7.5E-41 |
| *c* | | | 9.7E-27 |
| *d* | | | |

**Fig. S6F**
**Fig. S6I**

| Experiment | Genotype | Condition | Flies per vial | # vials | Total flies (set-up) | Deaths | Censors | Total flies (actual) | Median (d) |
|---|---|---|---|---|---|---|---|---|---|
| Lifespan (10 and 100 µM allopurinol) | $w^{Dah}$ females | 5%S –AP | 15 | 10 | 150 | 143 | 5 | 148 | 63.6 |
| | | 5%S +AP (10 µM) | 15 | 10 | 150 | 149 | 1 | 150 | 63.2 |
| | | 5%S +AP (100 µM) | 15 | 10 | 150 | 148 | 1 | 149 | 61.2 |
| | | 20%S –AP | 15 | 10 | 150 | 148 | 0 | 148 | 46.7 |
| | | 20%S +AP (10 µM) | 15 | 10 | 150 | 139 | 9 | 148 | 51.5 |
| | | 20%S +AP (100 µM) | 15 | 10 | 150 | 144 | 2 | 146 | 49.7 |

**P-value (Log-Rank test)**

| | *b* | *c* | *d* | *e* | *f* |
|---|---|---|---|---|---|
| *a* | 0.2670 | 1.2E-05 | 1.4E-53 | 4.4E-33 | 1.2E-28 |
| *b* | | 5.7E-04 | 5.0E-48 | 8.3E-29 | 4.5E-25 |
| *c* | | | 1.5E-36 | 4.1E-18 | 3.2E-15 |
| *d* | | | | 3.0E-08 | 2.5E-06 |
| *e* | | | | | 0.9977 |
| *f* | | | | | |

**Fig. S6J**

| Experiment | Genotype | Condition | Flies per vial | # vials | Total flies (set-up) | Deaths | Censors | Total flies (actual) | Median (d) |
|---|---|---|---|---|---|---|---|---|---|
| Lifespan (10 µM allopurinol) | $w^{Dah}$ females | 5%S –AP | 15 | 10 | 150 | 146 | 1 | 147 | 68.4 |
| | | 5%S +AP | 15 | 10 | 150 | 149 | 1 | 150 | 68.3 |
| | | 30%S –AP | 15 | 10 | 150 | 148 | 2 | 150 | 44.7 |
| | | 30%S +AP | 15 | 10 | 150 | 144 | 3 | 147 | 48.1 |

**P-value (Log-Rank test)**

| | *b* | *c* | *d* |
|---|---|---|---|
| *a* | 0.8028 | 1.9E-59 | 5.3E-52 |
| *b* | | 3.7E-65 | 3.0E-57 |
| *c* | | | 5.0E-06 |
| *d* | | | |

**Fig. S6K**

| Experiment | Genotype | Condition | Flies per vial | # vials | Total flies (set-up) | Deaths | Censors | Total flies (actual) | Median (d) |
|---|---|---|---|---|---|---|---|---|---|
| Lifespan (10 mM purine) | $w^{Dah}$ females | 5%S –$H_2O$ | 15 | 9 | 135 | 122 | 12 | 134 | 65.9 |
| | | 5%S +$H_2O$ | 15 | 9 | 135 | 113 | 18 | 131 | 68.1 |
| | | 5%S+purine –$H_2O$ | 15 | 10 | 150 | 148 | 1 | 149 | 59.4 |
| | | 5%S+purine +$H_2O$ | 15 | 10 | 150 | 132 | 17 | 149 | 69.2 |

**P-value (Log-Rank test)**

| | *b* | *c* | *d* |
|---|---|---|---|
| *a* | 0.2716 | 3.3E-14 | 0.0856 |
| *b* | | 1.2E-17 | 0.5854 |
| *c* | | | 3.6E-21 |
| *d* | | | |

**Table S4. Compounds Analysed by HILIC UPLC-HRMS Metabolomics in the *Drosophila* Rectal Ampulla Stones (Related to STAR Methods)**

| Compound | Mass (m/z) | Retention Time (min) | Formula | Polarity |
|---|---|---|---|---|
| Allopurinol | 135.03123 | 2.06 | $C_5H_4N_4O$ | Negative |
| $^{13}C_5$-Hypoxanthine | 140.04801 | 3.16 | $[^{13}C]_5H_4N_4O$ | Negative |
| Hypoxanthine | 135.03123 | 3.16 | $C_5H_4N_4O$ | Negative |
| Xanthine | 151.02615 | 3.90 | $C_5H_4N_4O_2$ | Negative |
| Allantoin | 157.03671 | 3.94 | $C_4H_6N_4O_3$ | Negative |
| Uric acid | 167.02106 | 10.98 | $C_5H_4N_4O_3$ | Negative |

## Table S5. Clinical Information on the Human Cohort (Related to STAR Methods)

| | Number of Participants | Age (years) | BMI ($kg/m^2$) | eGFR (mg/mL per 1.73 $m^2$) | Blood Glucose (mg/dL) |
|---|---|---|---|---|---|
| **Total** | 650 | 60 (51 - 69) | 26.5 (24.0 - 29.2) | 85 (77 - 96) | 96 (91 - 102) |
| **Males** | 367 | 60 (52 - 68) | 26.8 (24.8 - 29.3) | 86 (78 - 97) | 98 (93 - 103.5) |
| **Females** | 283 | 61 (51 - 70) | 25.9 (22.5 - 29.1) | 83 (75 - 95) | 94 (89 - 100) |

Age, body mass index (BMI), estimated glomerular filtration rate (eGFR), and blood glucose levels are given as median with range from lower (25%) to upper quartile (75%).
