# Supplementary material for: Sugar-Induced Obesity and Insulin Resistance Are Uncoupled from Shortened Survival in Drosophila
Source: Cell Metab. 2020 Apr 7;31(4):710–725.e7. doi: 10.1016/j.cmet.2020.02.016 (PMC7156915; doi:10.1016/j.cmet.2020.02.016)
Supplement: Document S2. Article plus Supplemental Information [file mmc2.pdf]

Cell Metabolism

Article

Sugar-Induced Obesity and Insulin Resistance Are Uncoupled from Shortened Survival in *Drosophila*

Graphical Abstract

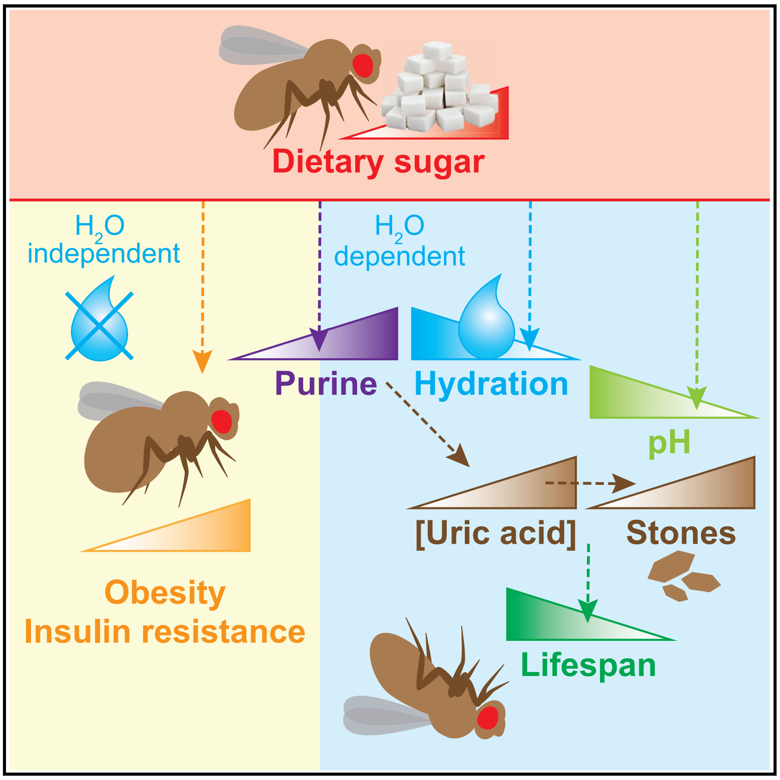

Authors

Esther van Dam,
Lucie A.G. van Leeuwen,
Eliano dos Santos, ..., Andre Franke,
Christoph Kaleta, Helena M. Cochemé

Correspondence

helena.cocheme@lms.mrc.ac.uk

In Brief

van Dam et al. disentangle the physiological effects of high-sugar feeding on survival using adult *Drosophila*. They uncouple diet-induced obesity and insulin resistance from shortened lifespan by rescuing sugar-induced water imbalance. Dietary sugar is linked to kidney dysfunction and dysregulation of purine catabolism in both flies and humans, emphasizing this pathway as a promising target for potential therapeutic interventions.

Highlights

- Sugar-induced lifespan shortening can be uncoupled from obesity and insulin resistance
- A high-sugar diet induces water imbalance in adult *Drosophila*
- High-sugar feeding shortens fly lifespan through dysregulation of purine catabolism
- Dietary sugar intake in humans is associated with circulating purine levels

van Dam et al., 2020, Cell Metabolism *31*, 710–725
April 7, 2020 © 2020 The Authors. Published by Elsevier Inc.
https://doi.org/10.1016/j.cmet.2020.02.016

Sugar-Induced Obesity and Insulin Resistance Are Uncoupled from Shortened Survival in *Drosophila*

Esther van Dam,[1,2,7] Lucie A.G. van Leeuwen,[1,2,7] Eliano dos Santos,[1,2,7] Joel James,[1,2,7] Lena Best,[3,7] Claudia Lennicke,[1,2] Alec J. Vincent,[1,2] Georgios Marinos,[3] Andrea Foley,[1,2] Marcela Buricova,[1,2] Joao B. Mokochinski,[1,2] Holger B. Kramer,[1,2] Wolfgang Lieb,[4] Matthias Laudes,[5] Andre Franke,[6] Christoph Kaleta,[3] and Helena M. Cochemé[1,2,8,*]
[1]MRC London Institute of Medical Sciences, Du Cane Road, London W12 0NN, UK
[2]Institute of Clinical Sciences, Imperial College London, Hammersmith Hospital Campus, Du Cane Road, London W12 0NN, UK
[3]Institute for Experimental Medicine, Kiel University, 24105 Kiel, Germany
[4]Institute of Epidemiology, Kiel University, 24105 Kiel, Germany
[5]Department of Internal Medicine I, University Hospital Schleswig-Holstein, 24105 Kiel, Germany
[6]Institute of Clinical Molecular Biology, Kiel University, 24105 Kiel, Germany
[7]These authors contributed equally
[8]Lead Contact
*Correspondence: helena.cocheme@lms.mrc.ac.uk
https://doi.org/10.1016/j.cmet.2020.02.016

SUMMARY

High-sugar diets cause thirst, obesity, and metabolic dysregulation, leading to diseases including type 2 diabetes and shortened lifespan. However, the impact of obesity and water imbalance on health and survival is complex and difficult to disentangle. Here, we show that high sugar induces dehydration in adult *Drosophila*, and water supplementation fully rescues their lifespan. Conversely, the metabolic defects are water-independent, showing uncoupling between sugar-induced obesity and insulin resistance with reduced survival *in vivo*. High-sugar diets promote accumulation of uric acid, an end-product of purine catabolism, and the formation of renal stones, a process aggravated by dehydration and physiological acidification. Importantly, regulating uric acid production impacts on lifespan in a water-dependent manner. Furthermore, metabolomics analysis in a human cohort reveals that dietary sugar intake strongly predicts circulating purine levels. Our model explains the pathophysiology of high-sugar diets independently of obesity and insulin resistance and highlights purine metabolism as a pro-longevity target.

INTRODUCTION

Diet-induced obesity and associated metabolic disorders such as type 2 diabetes (T2D) are a major global issue reaching epidemic proportions, widely attributed to the increased consumption of sugary foods and drinks, with serious consequences to human health (WHO, 2016, 2020). Many severe co-morbidities are associated with T2D (e.g., nephropathy and cardiomyopathy), and the life expectancy of individuals with T2D is significantly reduced (Chatterjee et al., 2017; DeFronzo et al., 2015). In fact, diet-induced obesity and age are major risk factors for developing T2D, yet despite clear correlations between insulin resistance, obesity, and aging, the exact underlying mechanisms still remain unclear (Chatterjee et al., 2017; DeFronzo et al., 2015). Thirst is an early symptom of hyperglycemia, as excess non-reabsorbed glucose is eliminated via enhanced urine production, resulting in dehydration. Yet the role of water balance in the context of sugar-induced metabolic disease and survival defects is not fully appreciated.

The combination of short lifespan, low costs, and ease of genetic manipulations, together with the evolutionary conservation of central signaling pathways regulating metabolism and energy homeostasis, make the fruit fly *Drosophila melanogaster* a powerful model system to study dietary interventions, metabolic disorders, and aging (Baker and Thummel, 2007; Mattila and Hietakangas, 2017; Owusu-Ansah and Perrimon, 2014;

Context and Significance

Excess consumption of sugar has been implicated in the global epidemic of obesity and metabolic disease. How sugar-rich diets are detrimental to health and longevity is still unclear, and the interplay with water intake is often overlooked. Here, Cochemé and colleagues disentangle the diabetic-like effects of high-sugar diets and shortened survival in *Drosophila* by manipulating water balance. They find that sugar fuels the purine degradation pathway, leading to the accumulation of uric acid as kidney stones. Consistent with the fly data, they show in a human cohort that dietary sugar intake is associated with kidney function and blood purine levels. This work highlights the purine pathway as an unappreciated regulator of survival and a potential therapeutic target for health and lifespan benefits.

 © 2020 The Authors. Published by Elsevier Inc.
This is an open access article under the CC BY license (http://creativecommons.org/licenses/by/4.0/).

Padmanabha and Baker, 2014). Importantly, the endocrine system of the fly is remarkably similar to mammals, and the insulin/insulin-like growth factor signaling (IIS) pathway is strongly evolutionarily conserved (Partridge et al., 2011; Riera et al., 2016; Teleman, 2009).

A high-sugar diet has been used to model diabetic complications in *Drosophila*, applied both to larvae and adults, causing a range of detrimental effects on growth, metabolism, glucose homeostasis, cardiac function, and lifespan (Morris et al., 2012; Musselman et al., 2011; Na et al., 2013; Rovenko et al., 2015). Like humans, adult flies fed a high-sugar diet develop hyperglycemia, insulin resistance, and obesity (Morris et al., 2012; Skorupa et al., 2008). High-sugar feeding also significantly shortens lifespan in flies (Lushchak et al., 2014; Na et al., 2013; Skorupa et al., 2008), which has been attributed to the associated metabolic defects, yet causal evidence is lacking. As flies are a particularly valuable *in vivo* model to study metabolism and aging, it is important to understand the mechanisms underlying the pathophysiology of high-sugar diets in order to strengthen potential translation for diabetes research and therapies.

In this study, we focus on the response of adult flies to a high-sugar diet and the regulation of lifespan. We develop an experimental setup allowing us to distinguish between phenotypes that are directly caused by the sugar and those that are related to other factors such as sugar-induced water imbalance. Strikingly, we show that sugar-induced obesity and insulin resistance are unlinked from organismal aging. Furthermore, we find that the high-sugar diet fuels increased purine catabolism, promoting uric acid accumulation and the formation of stones in the renal tubules, which are exacerbated by dehydration and physiological acidification. In agreement with our results in *Drosophila*, we show that dietary sugar intake is also a strong predictor of renal function and circulating purine levels in a large human cohort. Overall, this study has important implications for high-sugar diets as a paradigm of metabolic disease and offers molecular insights into the complex pathology of diabetes models and their intricate relationship with survival.

RESULTS

High-Sugar Feeding Induces Thirst and Dehydration in Adult *Drosophila*

Drosophila are able to actively regulate water intake in response to diet composition (Fanson et al., 2012). Water imbalance was previously shown to cause the shortened lifespan of adult flies fed a high-salt diet (Piper et al., 2010), stressing the importance of dehydration on survival. Therefore, to assess whether a high-sugar diet induced thirst and dehydration in *Drosophila*, we fed adult wild-type (WT) *white Dahomey* (w^{Dah}) flies a standard laboratory food containing 5% w/v sucrose (5%S), or a high-sugar diet with 4-fold increased levels of sucrose up to 20% w/v (20%S). The protein source, yeast, was maintained constant throughout all experiments (see Table S1 for all media recipes). An exogenous water source was provided to the flies in the form of an agar gel (Figure 1A), a strategy previously used to rescue salt toxicity and explore mechanisms of dietary restriction in adult *Drosophila* (Ja et al., 2009; Piper et al., 2010). To assay thirst, we maintained flies on the 5%S and 20%S diets ± H_2O for 7 days, then measured their drinking from an agar source using the automated FlyPAD system, where proboscis extension completes a circuit between electrodes and registers a signal (Itskov et al., 2014). Flies on the 20%S diet without additional water exhibited a significantly increased number of sips, which was abolished when flies were pre-treated with the 20%S diet supplemented with water (Figures 1B and 1C). As further corroboration, we assayed thirst by two additional independent methods: first, we exposed flies to agar containing blue dye and quantified the amount of water ingested colorimetrically (Figure S1A); and second, we performed an adaptation of the liquid Capillary Feeder (CAFE) system (Ja et al., 2007) to supply drinking water to the vials and measured the volume of water consumed by the flies (Figure S1B). Again, by both of these approaches, flies pre-treated with the 20%S diet were significantly thirstier, which disappeared upon water supplementation. Furthermore, by all three independent drinking assays, WT flies fed the 5%S control food did not drink additional water, indicating that our standard diet does not induce thirst in WT flies (Figures 1B, 1C, S1A, and S1B).

To test for dehydration, we assessed the volume of total hemolymph extracted from flies (i.e., the circulating interstitial fluid, as flies have an open circulatory system). Hemolymph volume on the 20%S diet was unchanged at day 7 (d7), but significantly decreased at day 28 (d28) (Figure 1D). This loss was fully rescued back to control levels by water supplementation (Figure 1D), consistent with the flies being dehydrated upon long-term chronic high-sucrose feeding when unable to replenish their thirst. Together, these data confirm that flies subjected to a high-sucrose diet experience thirst, and that supplementation with drinking water was able to rescue this sugar-induced dehydration. Importantly, this experimental setup allows us to distinguish between the water-dependent and water-independent effects of high-sugar feeding on lifespan and physiology in adult *Drosophila*.

Water Supplementation Fully Rescues the Shortened Lifespan of WT Adult Flies on a High-Sucrose Diet

To explore how this sugar-induced water imbalance affected adult physiology, we focused initially on WT aging. A high-sucrose diet has been reported to shorten adult lifespan (Lushchak et al., 2014; Skorupa et al., 2008), and we confirmed that WT female flies on the 20%S food were dramatically shorter-lived than on the standard 5%S food (~21% decrease in median lifespan; Figures 1E and S1C). Remarkably, supplementation of the 20%S diet with water fully rescued the lifespan back to control levels (Figures 1E and S1C). Furthermore, despite known deleterious effects of age on water balance (Gibbs and Markow, 2001; Zheng et al., 2018), supplementation of the control 5%S food with water had no effect on WT lifespan (Figures 1E and S1C), confirming that WT flies were not under water stress on our standard laboratory diet.

To exclude changes in feeding behavior as a potential confounder in our results, we assessed feeding rates on the high-sucrose diet. Importantly, while WT flies fed less on the 20%S diet compared to standard 5%S, water supplementation did not affect the level of feeding on each respective food type, as measured by observation of proboscis extension (Figure S1D) or excretion of dye-containing food (Figure S1E; Shell et al., 2018). This confirmed that the lifespan rescue of the

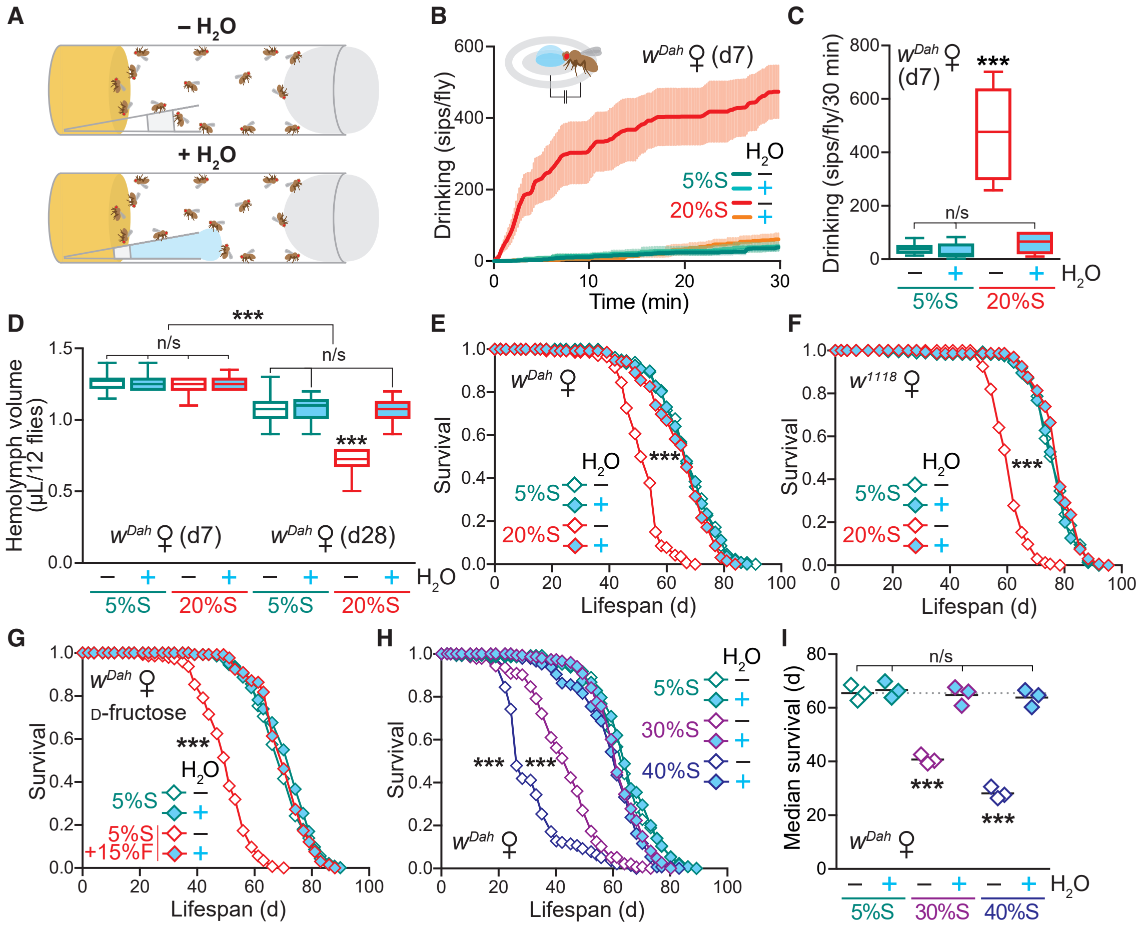

Figure 1. A High-Sucrose Diet that Induces Thirst and Dehydration and Shortens Lifespan in Adult *Drosophila* Is Rescued by Water Supplementation

(A) Scheme of the experimental setup, illustrating the supplementation of media vials with agar-filled tips as an *ad libitum* water source.

(B and C) Drinking assay to quantify the thirst of WT (w^{Dah}) flies in response to a high-sugar diet. Females were pre-treated for 7 days on a standard (5%S) or high-sucrose (20%S) diet ± H_2O. (B) Automated FlyPAD analysis showing the cumulative number of sips from an agar water source over 30 min. Data are means ± SEM of n = 6–8 individual flies per condition. (C) Box-and-whisker plots (min-max error bars) of the data from (B), analyzed by one-way ANOVA with Tukey correction (n/s, $p > 0.05$; ***$p < 0.001$).

(D) Hemolymph volume of WT females pre-treated for 7 and 28 days on 5%S or 20%S ± H_2O. Hemolymph was extracted from groups of 12 flies (n = 12–20 replicates per condition). Data are presented as box-and-whisker plots (min-max error bars), analyzed by one-way ANOVA with Tukey correction (n/s, $p > 0.05$; ***$p < 0.001$).

(E) Lifespan of WT females on 5%S and 20%S ± H_2O (n ~ 150 per condition).

(F) Lifespan of w^{1118} females on 5%S and 20%S ± H_2O (n ~ 165 per condition).

(G) Lifespan of WT females ± H_2O on a control diet (5%S) supplemented with 15% D-fructose (n ~ 150 per condition).

(H) Lifespan of WT females on 30%S and 40%S ± H_2O (n ~ 120–135 per condition).

(I) Summary of median survival ± H_2O for n = 3 independent lifespan experiments on control (5%S) and high sucrose (30%S and 40%S) diets. Data were analyzed by one-way ANOVA with Tukey correction (n/s, $p > 0.05$; ***$p < 0.001$).

Statistical analysis for all survival curves (E, F, G, and H) was performed by log-rank test (n/s, $p > 0.05$; ***$p < 0.001$). See Table S2 for exact n numbers and p values. See also Figure S1.

20%S + H_2O flies was not simply due to decreased ingestion of the "harmful" high-sugar food.

To rule out genetic background effects, we assessed the water response in different fly strains. Presence of the endosymbiont *Wolbachia* is known to affect fly metabolism and lifespan (Ikeya et al., 2009; Toivonen et al., 2007); therefore, we tested a *Wolbachia*-uninfected strain of w^{Dah} and found that it responded similarly to a high-sucrose diet, showing that the process was independent of *Wolbachia* status (Figure S1F). Furthermore, the parental (red-eyed) *Dahomey* strain behaved comparably to w^{Dah} (Figure S1G), indicating that the *white* mutation reported to influence feeding and behavior was not involved. Finally, we also tested the response in females of the isogenic control strain w^{1118} and again found a similar pattern (Figure 1F). Importantly, the water rescue was not gender-specific, as it was also observed in w^{1118} males (Figure S1H) and not linked to egg production, as it occurred in sterile ovo^{D1} mutant females (Figure S1I). We also quantified egg laying directly, which is often

regarded as an indicator of female *Drosophila* health and is frequently associated with a lifespan trade-off (Flatt, 2011). As previously reported for high-sugar diets (Lushchak et al., 2014), we observed lower fecundity on the 20%S food (Figure S1J). Interestingly, water supplementation did not rescue this decreased egg laying (Figure S1J) even though longevity was fully restored to control levels, representing an uncoupling of lifespan and fecundity. To control for the effects of decreased food intake between the 5%S and 20%S diets, which may explain the reduced fecundity, we supplemented the agar tips with a cocktail of essential amino acids (Grandison et al., 2009a); however, this did not improve egg laying on the 20%S food (Figure S1J). Therefore, not all phenotypes in response to a high-sugar diet can be rescued by water supplementation.

To address whether the water rescue of lifespan occurs on other high-sugar diets, we tested the constituent monosaccharides of sucrose, D-fructose and D-glucose. For these experiments, we performed lifespan assays with a standard sucrose base (5%S) supplemented with a 15% w/v excess of each respective sugar. We found that a high D-fructose (Figure 1G) or D-glucose (Figure S1K) diet significantly shortened adult lifespan to a similar extent as high sucrose (Figure S1L) and that this effect was fully rescued by water supplementation, confirming that our initial observations were not specific to sucrose.

To enhance the severity of the dietary challenge, we investigated the response of WT flies at even higher sugar concentrations, within a range used experimentally for *Drosophila*. When sucrose levels were further increased from 20%S to 30%S and 40%S, we found a corresponding dose-dependent decrease in lifespan compared to 5%S by ~38% and ~57%, respectively (Figures 1H and 1I). Remarkably, this greater decline in longevity was still fully rescued by water supplementation (Figure 1I). Overall, we have shown that water supplementation fully and robustly rescued the shortened lifespan from a high sucrose diet across multiple genetic background strains and in a gender-independent manner.

Response of Metabolic Traits to a High-Sucrose Diet and Water Supplementation

The shortened lifespan of adult flies on a high-sugar diet is widely assumed to be a consequence of the associated metabolic defects; namely obesity, glycation, hyperglycemia, and insulin resistance. Therefore, we were interested to investigate how water supplementation affected these high-sugar-induced metabolic phenotypes in the context of rescued lifespan. Consistent with previous reports (Morris et al., 2012; Skorupa et al., 2008), we confirmed that WT adult flies fed a high-sucrose diet had increased adiposity, both by lipid staining of fat body tissue (Figure 2A) and measurement of whole-body triglyceride (TAG) levels (Figure 2B). Remarkably, diet-induced obesity was unaffected by water supplementation, and the 20%S + H_2O flies retained their adiposity levels despite the rescued lifespan (Figure 2B).

Glycation damage has been implicated as an important factor underlying diabetic complications (Goh and Cooper, 2008). Advanced glycation end-products (AGEs), formed when reducing sugars such as D-glucose and D-fructose react non-enzymatically with amino groups on proteins, have been shown to increase in *Drosophila* with age (Jacobson et al., 2010; Oudes et al., 1998) and upon high-sugar feeding (Garrido et al., 2015). Proteins are typically glycated through lysine residues, forming adducts such as carboxy-methyl-lysine (CML). Using an anti-AGE (CML) antibody, we detected elevated glycation adducts in whole flies in response to 20%S feeding (Figures 2C and S2A). Interestingly, glycation levels were not affected by water supplementation, again demonstrating that despite the lifespan rescue, the 20%S + H_2O flies still displayed many of the supposedly harmful traits associated with high-sugar feeding. In contrast, as a control, protein carbonylation, indicative of oxidative damage, was unchanged for all conditions (Figures S2B and S2C).

To evaluate the effect of water supplementation on hyperglycemia induced by high-sugar feeding, we measured the levels of trehalose in the hemolymph, the major circulating sugar in *Drosophila* (Mattila and Hietakangas, 2017). Hemolymph trehalose levels were significantly increased on the 20%S diet, and this hyperglycemic state was retained upon water supplementation (Figure 2D). To monitor insulin resistance, we performed an insulin response test on the abdominal fat body tissue by assessing AKT phosphorylation. The 20%S diet caused insulin resistance, as evidenced from decreased phospho-AKT levels, which was not rescued by water supplementation (Figures 2E, 2F, and S2D). This was further corroborated by measuring glucose uptake into fat body tissue using a fluorescent non-metabolizable glucose analog (Figure 2G). Altogether, while the lifespan of WT flies was completely rescued on the 20%S + H_2O condition to that of "healthy" controls, they remained obese, hyperglycemic, and insulin resistant, which are hallmarks of metabolic disease. Therefore, we show that high-sugar-diet-induced "diabesity" can be fully uncoupled from mortality *in vivo*.

Heightened Sensitivity of *Δfoxo* Mutants in Response to Dietary Sucrose

Dietary sugar is sensed via the IIS pathway, which is a key regulator of growth, metabolism, and lifespan (Partridge et al., 2011; Riera et al., 2016; Teleman, 2009). Null flies for the transcription factor FOXO, the major downstream effector of the IIS pathway, have impaired nutrient sensing and are significantly shorter lived than WT (Slack et al., 2011; Figure S3A). Therefore, we were interested in testing the response of *Δfoxo* flies to a high-sugar diet and the effects of water supplementation. Again, we found that the 20%S diet induced thirst (Figures S3B and S3C) and shortened lifespan (Figures 3A, 3B, and S3A) in *Δfoxo* flies. However, this decreased survival was only partially rescued by water (Figures 3A, 3B, and S3A), suggesting a combination of both water-dependent and directly sugar-related effects. Contrary to WT flies, decreased hemolymph volume was already detectable in the *Δfoxo* mutants at d7 of 20%S treatment (Figure 3C), indicating a hypersensitivity to the dehydration effects of a high-sugar diet. Unexpectedly, and in contrast to WT flies, water supplementation further extended the lifespan of the *Δfoxo* mutant on the standard 5%S diet (Figures 3A and 3B). This result suggests that *Δfoxo* mutants, with their dysregulated IIS, were already experiencing dehydration stress under control sugar conditions.

To explore this observation further, we tested the effect of lowering dietary sucrose levels on survival of the *Δfoxo* mutant. Remarkably, simply decreasing the concentration of sucrose in the food from 5% to 2.5% was sufficient to abolish the

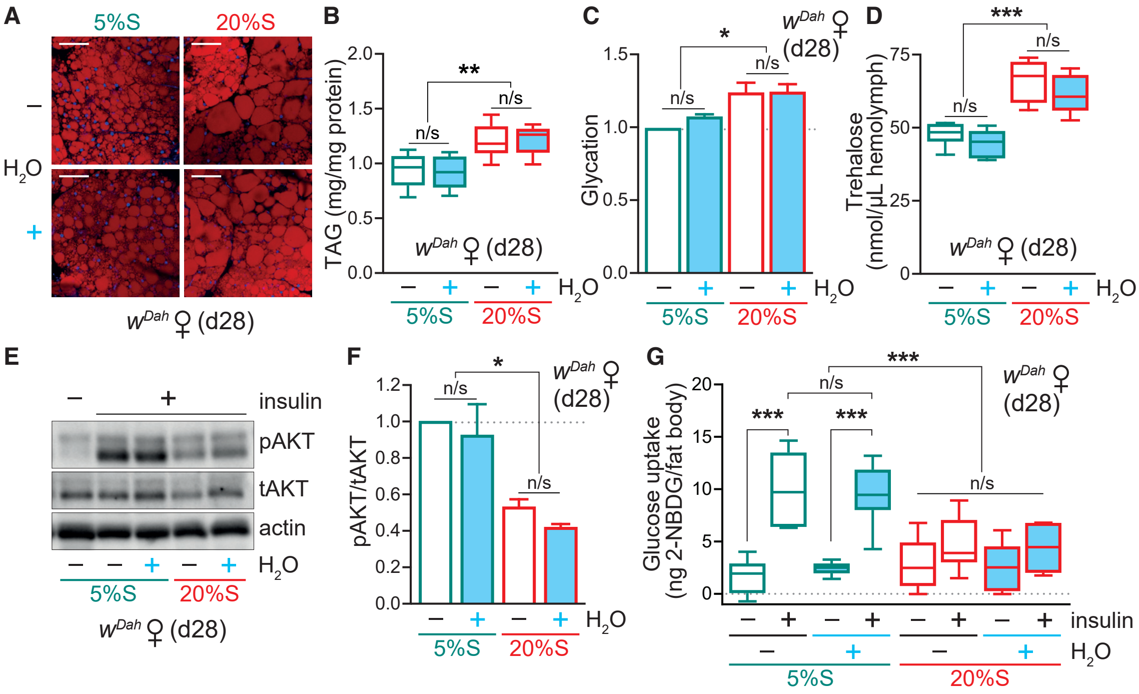

Figure 2. Metabolic Effects of a High-Sucrose Diet and Water Supplementation in WT Adults

(A) Lipid staining by Nile Red of abdominal fat body tissue from d28 WT (w^{Dah}) females fed on 5%S or 20%S ± H_2O, imaged by confocal microscopy. Scale bar: 50 μm.

(B) Whole body TAG levels in d28 WT females fed on 5%S or 20%S ± H_2O. Data (n = 8 replicates, each with n = 4 flies per sample) are presented as box-and-whisker plots (min-max error bars), analyzed by one-way ANOVA with Tukey correction (n/s, $p > 0.05$; **$p < 0.01$).

(C) Glycation damage in whole body d28 WT females fed on 5%S or 20%S ± H_2O, assessed by western blotting (see Figure S2A). Data are means + SEM of n = 4 samples per condition, analyzed by one-way ANOVA with Tukey correction (n/s, $p > 0.05$; *$p < 0.05$).

(D) Circulating trehalose levels in the hemolymph of d28 WT females pre-treated on 5%S or 20%S ± H_2O. Data (n = 6 replicates per condition, each with n = 12 flies per sample) are presented as box-and-whisker plots (min-max error bars), analyzed by one-way ANOVA with Tukey correction (n/s, $p > 0.05$; ***$p < 0.001$).

(E and F) Insulin response of abdominal fat body dissected from d28 WT females fed on 5%S or 20%S ± H_2O (see Figure S2D). (E) Western blot for phospho-AKT with total AKT and actin as controls. (F) Quantification of bands by densitometry. Data are means + SEM of n = 3 experiments (each with n = 5 fat bodies per sample), analyzed by one-way ANOVA with Tukey correction (n/s, $p > 0.05$; *$p < 0.05$).

(G) Glucose uptake into abdominal fat body dissected from d28 WT females fed on 5%S or 20%S ± H_2O (n = 6 replicates for "– insulin" and n = 10 replicates for "+ insulin," each with n = 5 fat bodies per sample). Data are presented as box-and-whisker plots (min-max error bars), analyzed by one-way ANOVA with Tukey correction (n/s, $p > 0.05$; ***$p < 0.001$).

See also Figure S2.

water-dependent lifespan extension (Figure 3D). Lowering the sucrose content further to 1% and 0% resulted in a concomitant decrease in survival, without any water differences (Figures S3D and S3E). Feeding rates of the *Δfoxo* flies were the same on the 1%S, 2.5%S and 5%S diets ± H_2O (Figure S3F), therefore excluding the modulation of lifespan via a dietary restriction mechanism. Consistent with our hypothesis, we performed drinking assays on aged *Δfoxo* flies treated for 21 days (rather than 28 days, since they are short-lived) and found that flies on the 5%S – H_2O diet were thirstier compared to the 5%S + H_2O condition, whereas drinking was unaffected on the 2.5%S diet ± H_2O (Figure 3E), highlighting their hypersensitivity to sucrose levels in the diet. The *Δfoxo* mutants have lower fecundity relative to WT (Slack et al., 2011), which was further decreased upon high-sugar feeding, regardless of H_2O status (Figure S3G), again showing an uncoupling between fecundity and longevity.

Importantly, as with WT flies, the 20%S diet also promoted obesity in the *Δfoxo* mutant, independently of water supplementation, as measured by whole body TAG content (Figure 3F). Furthermore, the TAG levels of *Δfoxo* flies on 5%S ± H_2O were indistinguishable, again uncoupling the obesity and longevity phenotypes. Overall, the *Δfoxo* mutants are hypersensitive to the deleterious effects of a high-sugar diet, even under control conditions of 5%S. Together, these data reveal that flies with dysregulated IIS are more vulnerable to the toxic effects of a high-sucrose diet. These results highlight the importance of considering water-dependent effects of dietary sugar, and that the optimal sugar level will vary according to the genetic context.

Effects of a High-Sugar Diet on Stress Responses and Gut Physiology

The shortened lifespan of flies on a high-sugar diet was assumed to be a consequence of their obesity and insulin resistance. Since this was not the case, we next sought other physiological explanations for the high-sugar-induced pathology. We first tested the response of WT flies to a range of stresses in the context of diet and water status, since survival is often correlated with stress resistance (Gems and Partridge, 2008). Consistent

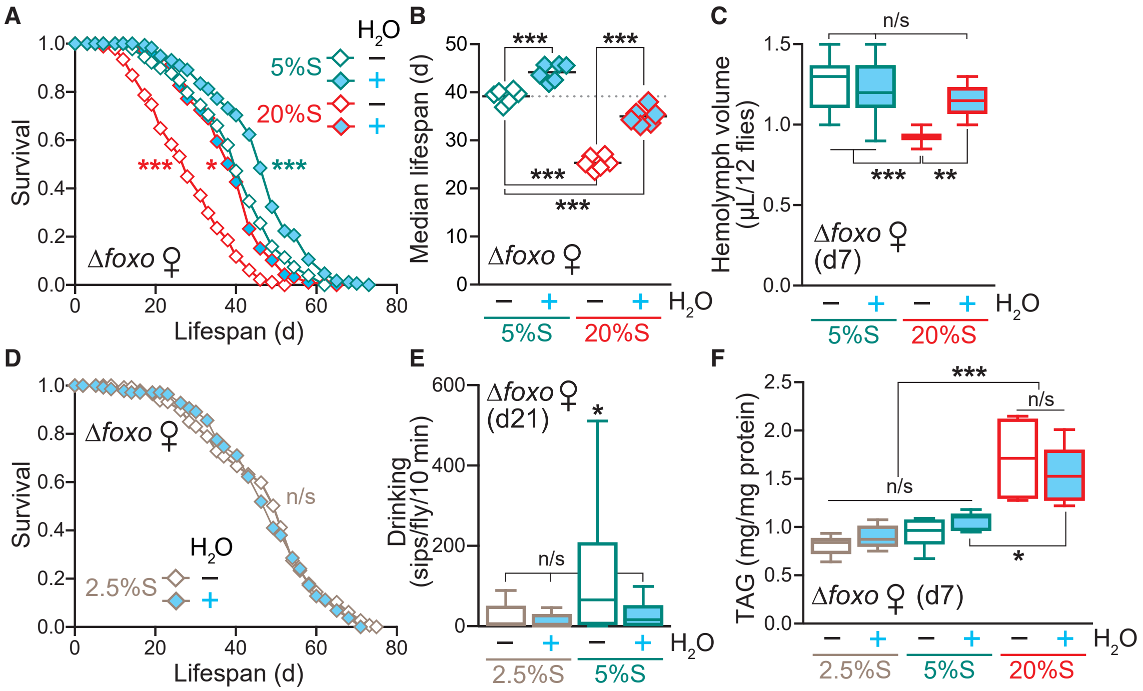

Figure 3. *Δfoxo* Mutants Are Hypersensitive to Dietary Sugar

(A) Lifespan of *Δfoxo* females on 5%S and 20%S ± H_2O (n ~ 195 flies per condition). Statistical analysis was performed by log-rank test (*p < 0.05; ***p < 0.001 against the 5%S – H_2O control). See Table S2 for exact n numbers and p values.

(B) Summary of median survival data for n = 6 independent *Δfoxo* lifespan experiments, analyzed by one-way ANOVA with Tukey correction (***p < 0.001).

(C) Hemolymph volume of *Δfoxo* females pre-treated for 7 days on 5%S or 20%S ± H_2O (n = 12 replicates per condition, each with n = 12 flies per sample). Data are presented as box-and-whisker plots (min-max error bars), analyzed by one-way ANOVA with Tukey correction (n/s, p > 0.05; **p < 0.01; ***p < 0.001).

(D) Lifespan of *Δfoxo* females on 2.5%S ± H_2O (n ~ 150 flies per condition). Statistical analysis was performed by log-rank test (n/s, p > 0.05). See Table S2 for exact n numbers and p values.

(E) Drinking assay of *Δfoxo* females pre-treated for 21 days on 2.5%S, 5%S, or 20%S ± H_2O measured over 10 min by FlyPAD (n = 30 individual flies per condition). Data are presented as box-and-whisker plots (min-max error bars), analyzed by one-way ANOVA with Tukey correction (n/s, p > 0.05; *p < 0.05).

(F) Whole body TAG levels of *Δfoxo* females pre-treated for 7 days on 2.5%S, 5%S, or 20%S ± H_2O (n = 5–6 replicates per condition, each with n = 4 flies per sample). Data are presented as box-and-whisker plots (min-max error bars), analyzed by one-way ANOVA with Tukey correction (n/s, p > 0.05; *p < 0.05; ***p < 0.001).

See also Figure S3.

with their sugar-induced dehydration (Figure 1D), d28 flies on the 20%S – H_2O condition were sensitive to desiccation stress, which was rescued by water pre-treatment (Figure 4A). We also subjected d7 flies pre-treated on 5%S or 20%S food ± H_2O to a subsequent high-salt challenge by transferring to a medium containing 500 mM NaCl (all without further water supplementation), and found that high-sugar feeding significantly sensitized the flies to high-salt stress (Figures 4B and S4A). Water supplementation during the 20%S high-sugar pre-treatment offered partial protection, but unlike lifespan, did not rescue the survival curve back to the 5%S response. This suggests that even though the high-sugar diet is supplemented with water, flies still endured sugar-induced damage that became apparent upon acute high-salt stress. In contrast, there was no difference between all conditions (5%S and 20%S ± H_2O) when flies were exposed to oxidative stress (Figure S4B), confirming that the effect is specific to metabolically induced dehydration stress. Interestingly, flies pre-treated on the 20%S diet were more resistant to starvation stress than 5%S flies, irrespective of water supplementation (Figure S4C), presumably reflecting their increased TAG reserves (Figures 2A and 2B). Therefore, stress responses cannot explain the full water-dependent rescue of WT lifespan on a high-sugar diet.

Since the gut is critical in the context of nutritional physiology (Miguel-Aliaga et al., 2018) and is the first organ exposed to the high-sugar diet, we next explored effects on intestinal function by analyzing the pattern of fly excreta assayed upon feeding the flies blue-dye-containing food (Cognigni et al., 2011; Figure S4D). The 20%S diet caused a pronounced decrease in the total number (Figure 4C) and size (Figure 4D) of deposits, both consistent with a dehydration effect (Cognigni et al., 2011). The 20%S diet also caused an alteration to the morphology of excreta, with an increase in oblong (reproductive oblong deposits [RODs]) versus circular deposits (Figure 4E); again, a reported signature of dehydration (Cognigni et al., 2011), and indeed all three parameters responded to water supplementation (Figures 4C–4E). Furthermore, the presence of a water source increased the lightness of deposits for both the 5%S and 20%S diets (Figure 4F), indicating that the excreta are more dilute. Therefore, the high-sucrose diet is exerting effects on gut physiology associated with water imbalance, which can be modulated by water supplementation.

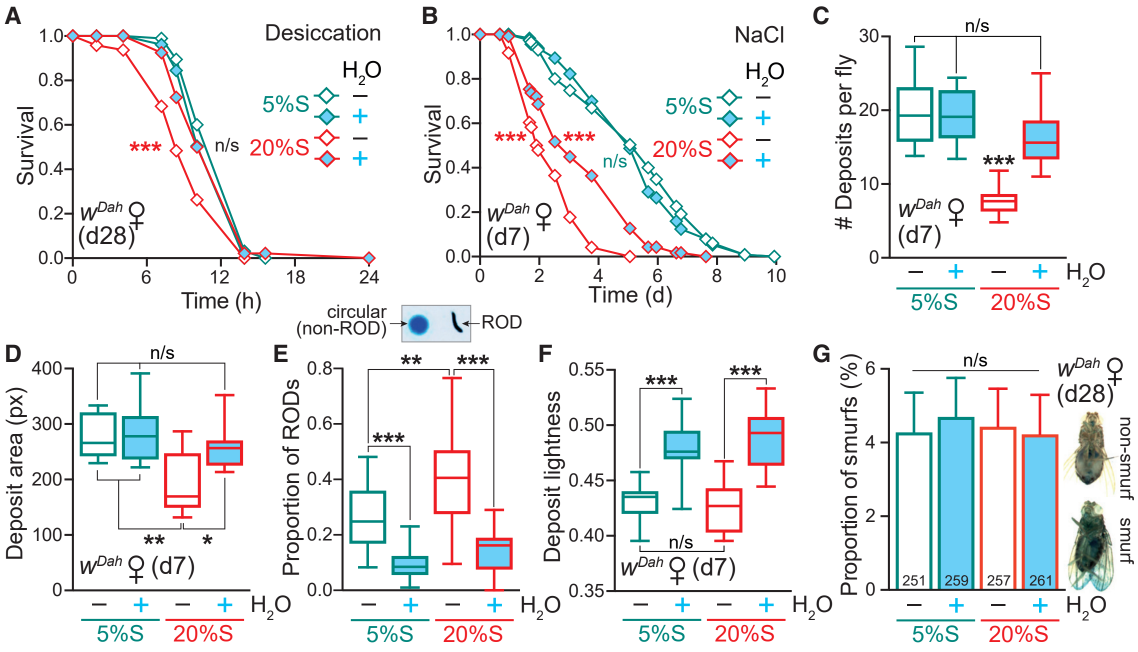

Figure 4. Effects of a High-Sugar Diet on Stress Responses and Gut Physiology

(A and B) Stress response of WT (w^{Dah}) females pre-treated on 5%S or 20%S ± H_2O, then exposed to (A) desiccation at d28 (n ~ 105 flies per condition) and (B) high salt at d7 (500 mM NaCl, n ~ 100–120 flies per condition). Statistical analysis of survival curves was performed by log-rank test (n/s, $p > 0.05$; ***$p < 0.001$). See Table S2 for exact n numbers and p values.

(C–F) Analysis of fly excreta from WT females pre-treated in vials for 7 days on 5%S or 20%S ± H_2O, then transferred to dishes for 24 h (n = 5 flies per plate). Food was supplemented with 2.5% w/v blue dye, while the agar for the water supplementation was undyed (see Figure S4D). Data are presented as box-and-whisker plots (min-max error bars), analyzed by one-way ANOVA with Tukey correction (n/s, $p > 0.05$; *$p < 0.05$; **$p < 0.01$; ***$p < 0.001$). (C) Number of deposits per fly recorded over 24 h (n = 10 plates per condition). (D) Mean area of deposits (n = 10 plates per condition). (E) Proportion of RODs (reproductive oblong deposits) (n = 21–24 plates per condition). (F) Mean lightness of deposits on a 0–1 scale (n = 10 plates per condition).

(G) The proportion of WT females exhibiting a Smurf phenotype at d28. Inset: example image of a non-Smurf fly, where the blue dye is restricted solely to the digestive tract, and a Smurf fly, where gut barrier integrity is compromised and the blue dye disperses throughout the fly body. Data are means + SEM of n = 20 vials per condition, analyzed by one-way ANOVA with Tukey correction (n/s, $p > 0.05$). The total number of flies scored per condition is indicated.
See also Figure S4.

To explore whether gut damage was responsible for the shortened lifespan of flies on a high-sugar diet, we assessed intestinal barrier function, an important parameter of gut health known to deteriorate with age, as determined by the Smurf assay, where flies are fed blue-dye-containing food and screened for dispersal of the dye beyond the digestive tract when gut integrity is compromised (Rera et al., 2012). Flies pre-treated for 28 days on 5%S or 20%S ± H_2O did not display a significant increase in the proportion of Smurfs (Figure 4G), indicating that even close to the onset of death, the barrier function of the gut was still intact in response to the high-sucrose diet. Therefore, although gut physiology was influenced by the high-sugar diet and water supplementation, gut dysfunction does not appear to underlie the lifespan-shortening effect.

A High-Sucrose Diet Is Associated with Tubule Dysfunction from Dysregulation of Purine Catabolism Causing Kidney Stones

In view of the water imbalance, we next assessed the function of the Malpighian tubules, the fly equivalent of the kidney (Figure 5A), which are vital organs for excretion, osmoregulation, and water homeostasis (Dow and Romero, 2010). To investigate the effects of a high-sugar diet on renal physiology, we first examined tubule morphology by light microscopy (Figure S5A). Flies fed the 20%S – H_2O diet exhibited a strongly darkened tubule phenotype, which was fully rescued by water supplementation (Figures 5B and 5C). These dark deposits were intraluminal concretions suggestive of uric acid accumulation (Ghimire et al., 2019). Therefore, we assayed uric acid content biochemically from whole flies (Figure 5D) and dissected tubules (Figure S5B), which was significantly elevated on the 20%S diet (~2- and ~4-fold, respectively) and again fully restored to the 5%S control baseline by water supplementation. In addition to the high-sucrose diet, excess D-glucose or D-fructose also led to the development of tubule stones, which were rescued by water (Figure S5C). We hypothesized that dietary sugars provided precursors for purine biosynthesis fueling the increased levels of uric acid, a waste product derived from purine catabolism (Figure 5E).

To explore the potentially pathogenic role of uric acid accumulation, we treated flies with the purine analog allopurinol (AP), a xanthine oxidase (XO) inhibitor that blocks uric acid production (Bratty et al., 2011; Figure 5E). Supplementation of the 20%S diet with AP (1 mM) completely abolished the elevated uric acid levels in the flies (Figure 5F) and the tubule stones (Figures 5G and 5H). Furthermore, consistent with their hypersensitivity to dietary sugar, aged *Δfoxo* flies developed increased tubule

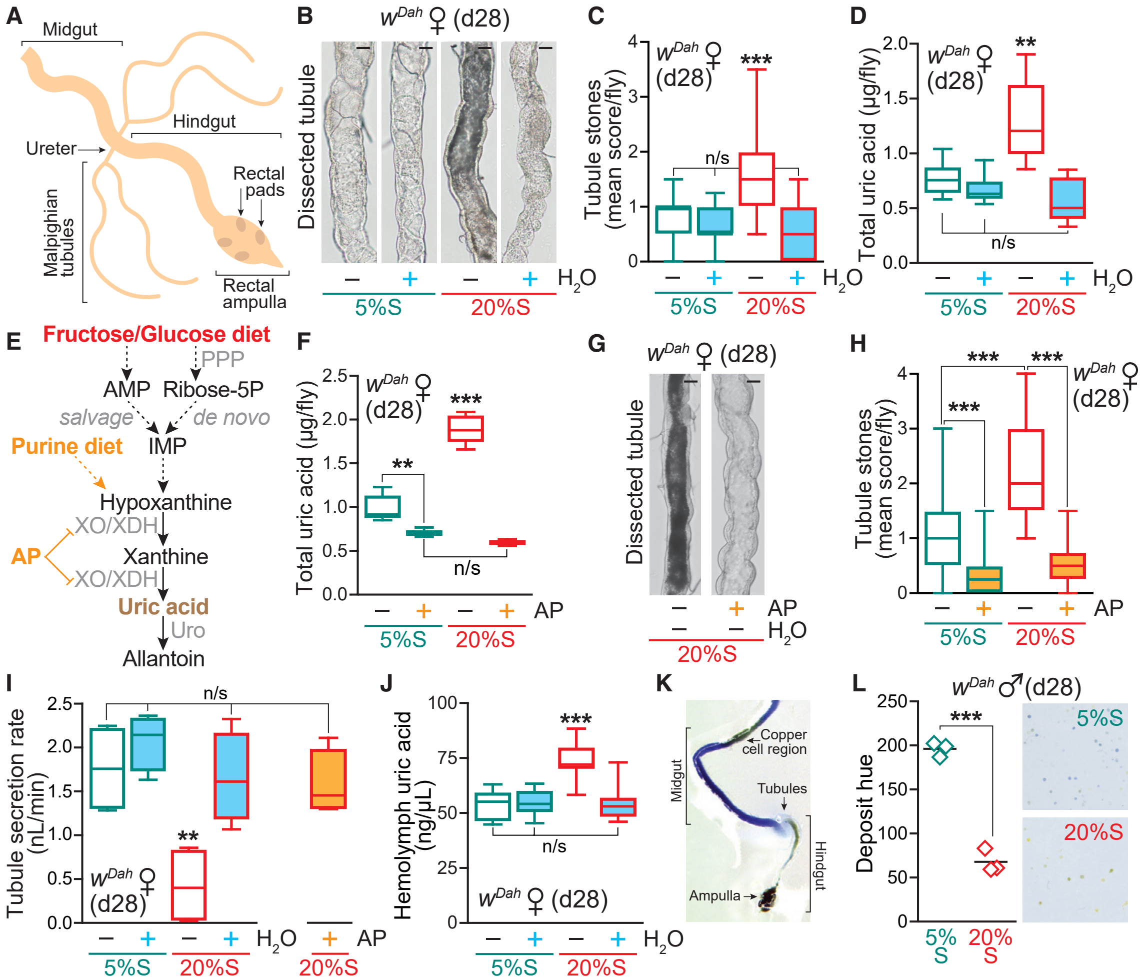

Figure 5. A High-Sugar Diet Induces Uric Acid Deposition and Tubule Dysfunction

(A) Diagram of the *Drosophila* lower digestive tract, terminating in the rectal ampulla. The Malpighian (renal) tubules connect at the junction between the midgut and the hindgut.

(B) Light microscopy images of dissected tubules from WT (w^{Dah}) females fed for 28 days on 5%S or 20%S ± H_2O. Scale bar: 25 µm.

(C) Tubule phenotype scoring of WT females maintained for 28 days on 5%S or 20%S ± H_2O. Data (n = 50 flies per condition) are presented as box-and-whisker plots (min-max error bars), analyzed by Kruskal-Wallis test with Dunn correction (n/s, $p > 0.05$; ***$p < 0.001$). See Figure S5A for the scoring scale.

(D) Uric acid content of whole WT females fed for 28 days on 5%S or 20%S ± H_2O (n = 6 replicates per condition, each with n = 5 flies per sample). Data are presented as box-and-whisker plots (min-max error bars), analyzed by one-way ANOVA with Tukey correction (n/s, $p > 0.05$; **$p < 0.01$).

(E) Biochemical pathway showing how dietary sugars such as D-glucose and D-fructose can lead to enhanced purine metabolism and to the formation of uric acid. Following the action of enzymes such as hexokinase, adenosine monophosphate (AMP) is formed and can be converted to the purine precursor inosine monophosphate (IMP). Alternatively, the purine precursor ribose-5-P is formed via the pentose phosphate pathway (PPP) by *de novo* biosynthesis. The drug allopurinol (AP) prevents uric acid production by inhibiting the enzyme xanthine oxidase (XO)/xanthine dehydrogenase (XDH). Urate oxidase (Uro) catalyzes the degradation of uric acid to allantoin. The enzyme XO/XDH (encoded by *rosy* in *Drosophila*) and Uro are strongly expressed in the tubules (>20-fold and >13,000-fold higher than in the hindgut of females, respectively; Leader et al., 2018).

(F) Uric acid content of whole WT females fed for 28 days on 5%S or 20%S ± AP (1 mM). Box-and-whisker plots (min-max error bars) of n = 5 replicates per condition (each with n = 5 flies per sample), analyzed by one-way ANOVA with Tukey correction (n/s, $p > 0.05$; **$p < 0.01$; ***$p < 0.001$).

(G) Light microscopy images of dissected tubules from WT females fed for 28 days on 20%S ± AP (1 mM). Scale bar: 25 µm.

(H) Tubule phenotype scoring of WT females maintained for 28 days on 5%S or 20%S ± AP (1 mM). Data (n = 50 flies per condition, except n = 25 for 20%S + AP) are presented as box-and-whisker plots (min-max error bars), analyzed by Kruskal-Wallis test with Dunn correction (***$p < 0.001$). See Figure S5A for the scoring scale.

(I) Secretion rates of tubules from WT females pre-treated for 28 days on 5%S or 20%S ± H_2O or ± AP (1 mM). See Figure S5E for a diagram of the secretion assay. Data (n = 4 replicates per condition) are presented as box-and-whisker plots (min-max error bars), analyzed by one-way ANOVA with Tukey correction (n/s, $p > 0.05$; **$p < 0.01$).

(J) Hemolymph uric acid concentration from WT females fed for 28 days on 5%S or 20%S ± H_2O. Data (n = 8 replicates per condition, each with n = 12 flies per sample) are presented as box-and-whisker plots (min-max error bars), analyzed by one-way ANOVA with Tukey correction (n/s, $p > 0.05$; ***$p < 0.001$).

(legend continued on next page)

stones on the 5%S diet (Figure S5D). Therefore, the tubule stone pathology is water-dependent, can be induced by a high-sugar diet, and modulated both pharmacologically and genetically.

To investigate the effects of a high-sugar diet and uric acid deposition on tubule physiology and function, we assayed the secretion rates of dissected WT tubules *ex vivo* (Figure S5E). Consistent with blockage by the intraluminal concretions, flies fed the 20%S diet for 28 days displayed severely compromised tubule secretion rates of ~0.5 nL/min compared to ~1.5-2 nL/min for controls (Figure 5I). Importantly, the impaired tubule secretion on a high-sugar diet was fully rescued by water supplementation, as well as by AP treatment (Figure 5I).

Multiple factors contribute to uric acid stone formation, including uric acid concentration and pH (Moe, 2006). First, we measured circulating uric acid levels in the fly hemolymph in response to a high-sugar diet. The concentration of uric acid was significantly increased at d28 on the 20%S diet, exacerbated by the hemolymph dehydration, and was rescued back to control levels by water supplementation (Figures 5J and S5F). Second, since uric acid crystallization is favored at acidic pH (Moe, 2006), we assessed hemolymph and urine pH in response to a high-sugar diet. Despite the significantly decreased hemolymph volume, we did not detect changes in hemolymph pH at d28 (Figure S5G), suggesting tight homeostatic control. Next, to assess urine pH, we supplemented the fly food with a pH indicator dye (Cognigni et al., 2011). Since in *Drosophila* the renal tubules connect to the digestive tract at the hindgut (see Figure 5A), the deposits produced by the fly are combined urine and feces. Consistent with previous observations (Cognigni et al., 2011), dissection of the digestive tract from dye-fed flies confirmed acidification of the hindgut posterior to the tubules (Figure 5K). Moreover, our excreta analysis revealed pronounced acidification of deposits in response to the 20%S food (Figures 5L and S5H). Therefore, the high-sugar diet results in both elevated uric acid levels and acidified conditions, which promote uric acid stone formation.

Pharmacological Treatments and Dietary Interventions Targeting Purine Metabolism Impact on Lifespan

To gain mechanistic insight and test whether uric acid accumulation upon high-sugar feeding contributed to the shortened survival, we performed lifespan experiments with AP supplementation in the food. Interestingly, a chronic high dose of the XO inhibitor AP (1 mM) shortened lifespan on both control and high-sugar diets (Figure S6A), despite clearing the tubules (Figures 5G and 5H). Upon dissection of AP-treated flies, we observed substantial concretions in the rectal ampulla (Figures 6A, S6B, and S6C), the final anatomical region of the *Drosophila* hindgut (see Figure 5A), with ~80% of control 5%S + AP flies and 100% of high-sugar 20%S + AP flies exhibiting ampulla stones at d28, compared to only <10% for both diets without the drug (Figure 6B). This severe pathology is consistent with the shortened lifespan and has previously been described in honeybees (termed “rectal enteroliths”) in the context of colony collapse disorder (vanEngelsdorp et al., 2017) and recently in a *Drosophila* model of kidney disease by RNAi of urate oxidase (Lang et al., 2019). The high-sugar diet (20%S) alone caused a moderate but significant increase in the presence and severity of rectal ampulla stones, which was fully rescued to control levels by water supplementation (Figure 6B). Metabolomics analysis confirmed that the rectal ampulla stones formed on the high-sugar diet (20%S) contained high levels of uric acid, consistent with dysregulation of purine catabolism (Figure S6D; Table S4). Rectal ampulla stones upon AP treatment were composed of xanthine and hypoxanthine, highlighting the accumulation of upstream metabolites in the purine degradation pathway when uric acid formation is blocked (Figures S6D and S6E). The rectal ampulla is involved in pH regulation (Cognigni et al., 2011) and is known to be acidified (Figure 5K), explaining why this region of the digestive tract was particularly susceptible to the formation of stones. Throughout our study, stones were never observed in the midgut (including the acidic copper cell region; see Figure 5K), consistent with flow of stone-precursor metabolites from the tubules into the hindgut.

To overcome this toxicity, we tested a lower AP dose (100 μM) and found that this treatment extended the survival of WT females on a high-sugar diet (20%S ± AP), despite shortening lifespan on control food (5%S ± AP; Figures 6C and S6F). AP supplementation (100 μM) for 7 days did not affect food intake or fecundity on either the 5%S or 20%S diets (Figures S6G and S6H), suggesting that this dose does not have obvious adverse physiological effects in younger flies. Further decreasing the AP concentration to 10 μM had beneficial longevity effects on the high-sugar diet without shortening the survival of flies on the control food 5%S ± AP (Figures S6I and S6J). This partial lifespan rescue by AP indicated that uric acid production contributed to the decreased longevity induced by high-sugar diets. Therefore, to recapitulate the dysregulation of purine catabolism observed on a high-sugar diet, we directly fed flies a high-purine diet. Supplementing our standard 5%S diet with excess purine led to increased levels of uric acid (Figure 6D) and the formation of tubule stones (Figure 6E) to a similar extent as the high-sugar diet, which were both rescued to control levels by water supplementation. Importantly, a high-purine diet significantly shortened lifespan, which was fully rescued by water supplementation (Figures 6F and S6K). Together, these pharmacological and dietary modulations confirmed that dysregulation of purine catabolism as a consequence of high-sugar feeding caused water-dependent uric acid accumulation and concretions underlying decreased lifespan.

Human Metabolomics Analysis Links Dietary Sugar Intake with Renal Function and Circulating Purine Levels

Next, to test whether dietary habits could be linked to circulating purine levels in humans, we analyzed a population cohort of 650

(K) Dissected WT female gut after feeding with the pH indicator dye bromophenol blue (0.5% w/v) showing acidification of the hindgut (posterior to the tubules) and the rectal ampulla. The copper cell region in the midgut, known to be acidified, is also apparent.

(L) Physiological acidification in response to the high-sugar diet. WT males were pre-treated for 28 days on 5%S or 20%S, then incubated in plates with medium containing the pH indicator dye bromocresol purple (0.5% w/v) for 48 h. Mean hue of deposits (n = 3 plates per condition), analyzed by unpaired two-tailed Student’s t test (***$p < 0.001$). Inset: scan of a typical plate illustrating the pH-dependent color shift (see Figure S5H).

See also Figure S5.

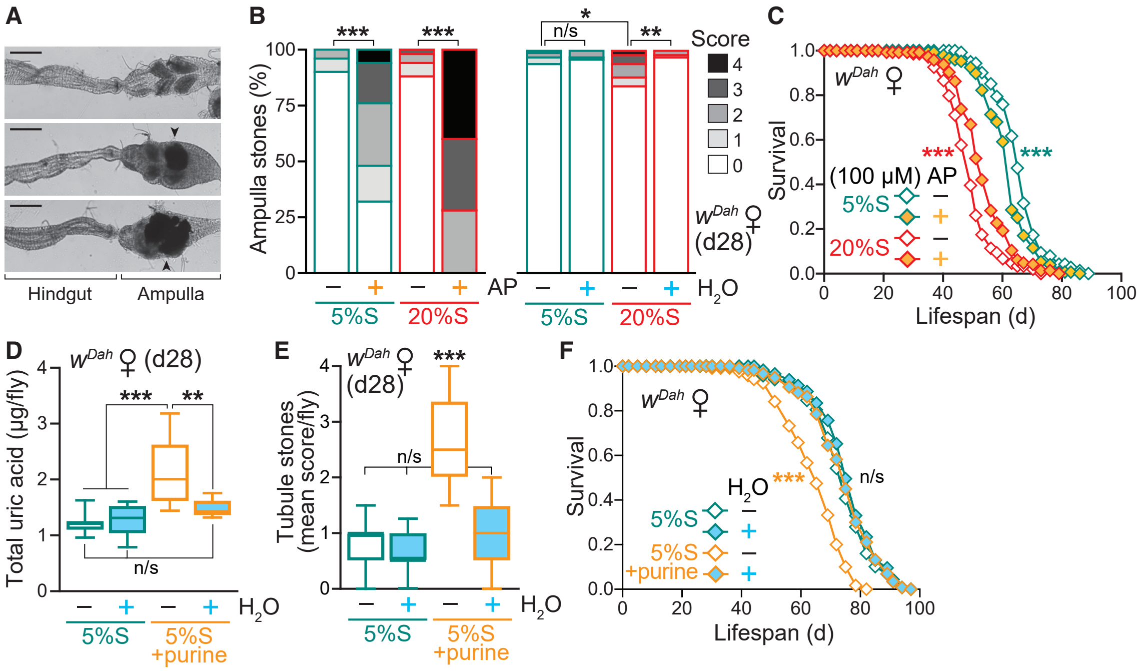

Figure 6. Pharmacological Treatments and Dietary Interventions Targeting Purine Metabolism Impact on Lifespan

(A) Light microscopy images of dissected ampulla: above, a clear ampulla with rectal pads visible; below, examples of stones present in the ampulla. Scale bar: 100 µm.

(B) Rectal ampulla stone phenotype scoring of WT (w^{Dah}) females pre-treated for 28 days on 5%S or 20%S ± AP (1 mM) (n = 50 flies per condition, except n = 25 for 20%S + AP) and 5%S or 20%S ± H_2O (n = 100 flies per condition). Data were analyzed by Kruskal-Wallis test with Dunn correction (n/s, $p > 0.05$; *$p < 0.05$; **$p < 0.01$; ***$p < 0.001$). See Figure S6B for the scoring scale.

(C) Allopurinol treatment (100 µM) extends the survival of WT females on a high-sucrose diet (20%S ± AP), despite shortening lifespan on control food (5%S ± AP) (n ~ 150 flies per condition).

(D) Uric acid content of WT females is elevated after 28 days on a high purine diet (5%S + 10 mM purine), and rescued by water supplementation. Data (n = 8 replicates per condition, each with n = 5 flies per sample) are presented as box-and-whisker plots (min-max error bars), analyzed by one-way ANOVA with Tukey correction (n/s, $p > 0.05$; **$p < 0.01$; ***$p < 0.001$).

(E) Tubule phenotype scoring of females maintained for 28 days on a high-purine diet (5%S + 10 mM purine) ± H_2O. Data (n = 50–60 flies per condition) are presented as box-and-whisker plots (min-max error bars), analyzed by Kruskal-Wallis test with Dunn correction (n/s, $p > 0.05$; ***$p < 0.001$). See Figure S5A for the scoring scale.

(F) A high purine diet (10 mM) shortens lifespan, which is fully rescued by water supplementation (n ~ 120 flies per condition).

Statistical analysis of survival curves (C and F) was performed by log-rank test (n/s, $p > 0.05$; ***$p < 0.001$). See Table S2 for exact n numbers and p values. See also Figure S6.

individuals (Figure 7A). This dataset combines extensive phenotypic information, including clinical parameters (Table S5), with detailed dietary records and comprehensive blood metabolomics (Brandstetter et al., 1999; Koch et al., 2017; Müller et al., 2015). The diet composition was evaluated from food questionnaires for each individual study participant. The estimated glomerular filtration rate (eGFR) determined from creatinine levels in the blood serum (Levey et al., 2006) served as a predictor of renal function and kidney health in our human study subjects. Concentrations of the purines guanosine, xanthosine, inosine, xanthine, hypoxanthine, and uric acid were measured by liquid chromatography-mass spectrometry (LC-MS) from serum samples (Figure S7A).

Using linear modeling, we assessed the direction of interaction between eGFR and each individual circulating purine. Serum levels of guanosine and inosine, corresponding to precursors in the purine metabolic pathway, were negatively associated with kidney function (Figure 7B). Additionally, we found a very strong negative correlation of uric acid serum levels with eGFR in our cohort (Figures 7B and S7B), which is in line with uric acid concentrations as a predictor of chronic kidney disease progression (Bellomo et al., 2010; Johnson et al., 2013a).

Variation of the eGFR was tested against the intake of dietary food groups while controlling for relevant clinical parameters, notably the participant's age and BMI, as well as blood plasma levels of glucose and triglycerides. We found that eGFR was very strongly associated with age, as well as the consumption of sweets, which are rich in sucrose and other sugars (Figure 7C). Thus, the consumption of high-sugar-containing food explains variations in renal function in our human cohort.

To obtain further insight into the association between diet and renal function, we derived the molecular composition of all food items consumed, and observed that sugars, but interestingly, not dietary purines, were associated with eGFR (Figure 7D). Dietary sugar intake by many study participants could mainly be linked to the consumption of sweets and fruits; whereas for some individuals, total dietary sugars were almost exclusively derived from soft drinks or juices (Figure S7C). Milk products, likely due to lactose

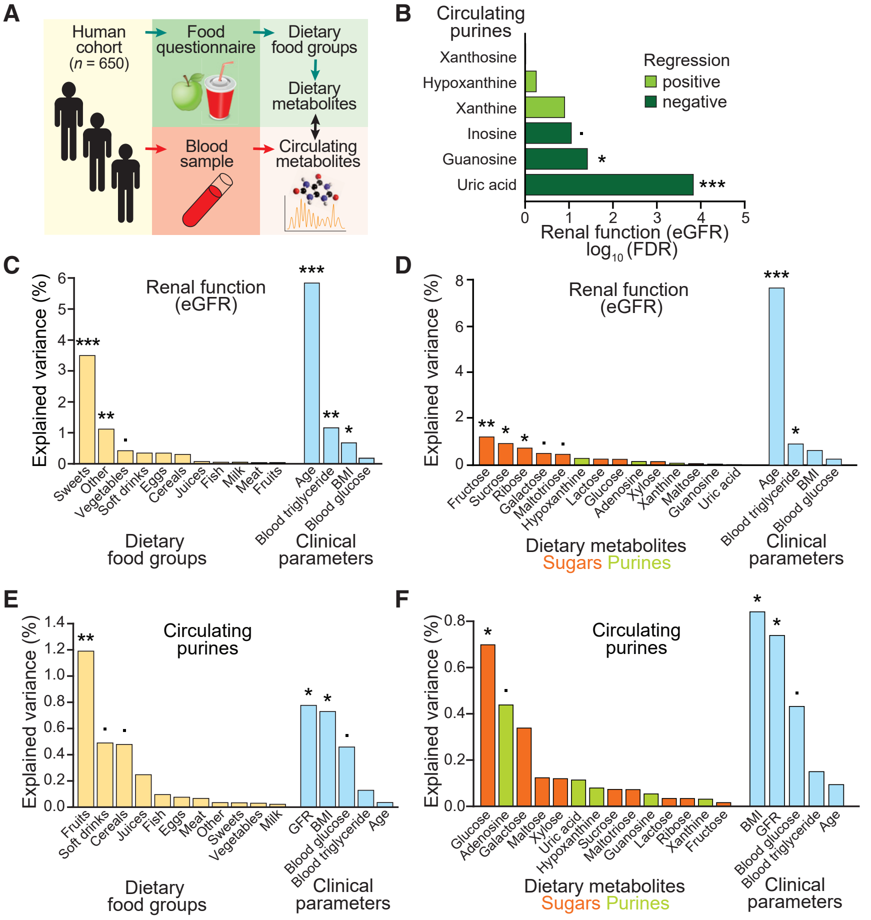

Figure 7. Human Metabolomics Analysis Links Dietary Sugar Intake with Renal Function and Circulating Purine Levels

(A) Scheme of the experimental setup to assess dietary intake and circulating metabolites in a German population cohort (n = 650). Dietary habits and food choices were recorded via the EPIC food frequency questionnaire for the past 12 months on the day of examination and used to impute dietary intake of individual metabolites. Blood was drawn at a single time point, and the serum was subjected to metabolomics by LC-MS to obtain levels of circulating metabolites.

(B) Linear model of eGFR predicting concentrations of each individual circulating purine. Logarithmic FDRs are plotted as bars and color-coded for positive (light green) or negative (dark green) regressions.

(C and D) Explained variance of diet on eGFR via PERMANOVA (· $p < 0.1$; *$p < 0.05$; **$p < 0.01$; ***$p < 0.001$). Clinical parameters are separated from dietary food groups for visual clarity. (C) Analysis of variance for dietary food groups. The food items contributing significantly from the "other" group were fats and oils (**) and non-alcoholic beverages (*). (D) Analysis of variance for imputed dietary metabolites, color-coded in orange for sugars and green for purines.

(E and F) Explained variance of diet on the levels of circulating purines in the serum via PERMANOVA (· $p < 0.1$; *$p < 0.05$; **$p < 0.01$). Clinical parameters are separated from dietary food groups for visual clarity. Analysis of variance for dietary food groups (E), and for imputed dietary metabolites, color-coded in orange for sugars and green for purines (F).

See also Figure S7.

content, and other food groups contributed only moderately to the total sugar intake of individual diets (Figure S7C).

To further strengthen the link between dietary habits (i.e., sugar consumption) and serum purine levels, we tested the association between circulating purines and dietary food groups while controlling for relevant clinical parameters, including eGFR. Our metabolomics data showed a significant interaction between circulating levels of purines and the consumption of fruits, as well as a marginally significant interaction with soft drinks and cereals (Figure 7E). All these food groups are rich in carbohydrates: soft drinks are expected to be rich in sucrose while cereals mainly contain starch, and fruits are known to have high fructose and glucose content. Surprisingly, circulating purine levels in the serum were not associated with the consumption of purine-rich food groups such as fish and meat (Figure 7E). In order to exclude that dietary intake of these food items was not a general modulator of serum metabolome, we performed a further analysis for circulating fatty acids. We found that levels of circulating fatty acids were most strongly influenced by the consumption of fish and meat (Figures S7D and S7E). Therefore, dietary intake of foods rich in carbohydrates and sugars can strongly predict circulating purine levels, but not fatty acids, in human serum.

In order to identify the specific dietary metabolites within these food groups that most strongly influenced circulating purine levels, we assessed their imputed molecular composition and found that dietary glucose was most strongly associated with circulating purine levels (Figure 7F). As dietary habits can differ between females and males, the previous analysis of the full cohort (n = 650) was repeated after stratification by gender. In female participants (n = 283), the associations did not reach significance thresholds (Figure S7F); however, in males (n = 367), dietary glucose intake was confirmed to be significantly associated with circulating purine levels (Figure S7G). Dietary glucose intake of most study participants could be mainly attributed to the consumption of fruits and sweets; whereas for some individuals, total dietary glucose was almost exclusively derived from soft drinks (Figure S7H). We also detected a strong correlation between dietary intake of glucose and fructose in our cohort (Figure S7I). Furthermore, using analysis of variance (Figures 7E and 7F) and linear modeling (Figure S7J), we found that BMI was significantly correlated with circulating purine levels, and in concordance with a recent study profiling individuals with obesity (Cirulli et al., 2019), especially positively correlated with serum uric acid (Figure S7K). Altogether, from our metabolomics analysis of a human cohort, circulating purine levels were positively correlated to dietary sugar intake, specifically glucose, with the consumption of fruits, sweets, and soft drinks being the most likely contributing food groups. We also found that eGFR was strongly associated with dietary sugars and the

consumption of sweets, as well as negatively correlated with circulating levels of the purines guanosine and uric acid. Therefore, consistent with our findings in *Drosophila*, a high-sugar diet also influences renal function and purine metabolism in humans.

DISCUSSION

Obesity is a major public health concern, with over 650 million adults worldwide estimated as clinically obese (WHO, 2020). Much of this obesity epidemic is attributed to increased consumption of sugary foods and drinks (Lustig et al., 2012). These lifestyle factors have been implicated in decreased human life expectancy, although the impact of obesity on health and survival is complex and not fully understood (Flegal et al., 2013; Hughes, 2013; Ortega et al., 2018). In this study, we show that a high-sugar diet causes a complex pathophysiological response in adult *Drosophila*. High-sugar feeding induced thirst, obesity, hyperglycemia, insulin resistance, and glycation damage—the classical hallmarks of T2D and metabolic disease. Critically, the lifespan-shortening effect of a high-sugar diet was rescued by water supplementation, whereas the metabolic pathologies of obesity and insulin resistance, widely attributed as responsible for the decreased survival, were in fact water independent. Indeed, flies on the high-sugar diet supplemented with water still exhibited all the diabetic-like metabolic defects, yet remarkably had the same survival as healthy controls, showing that obesity and insulin resistance per se do not shorten lifespan. Therefore, our study provides an elegant system to uncouple these aging and disease phenotypes, which is particularly interesting in light of the "fat but fit" paradox (Flegal et al., 2013; Hughes, 2013; Ortega et al., 2018).

We have demonstrated that the lifespan-shortening effects of a high-sugar diet occur via enhanced supply of metabolic precursors for purine biosynthesis, fueling increased purine catabolism. Our finding that *Δfoxo* mutants were hypersensitive to dietary sugar and the formation of tubule stones is consistent with a recent report that FOXO overexpression protects against the formation of uric acid concretions on a high-yeast diet in a fly model of urate oxidase deficiency (Lang et al., 2019). Furthermore, an SNP in human FOXO3 was found to be significantly associated with serum uric acid levels (Lang et al., 2019). Together, these observations highlight the potentially conserved role of the IIS pathway in regulating purine metabolism. From a human population cohort comprising 650 participants for which comprehensive serum metabolomics and detailed dietary records were available, we found that consumption of sugar-rich foods displayed a strong association with circulating purine levels. At the molecular level, the strongest predictor of circulating purine levels was dietary glucose intake. Our observations are supported by an earlier study, which focused only on the consumption of soft drinks and juices, and showed an association with serum uric acid measurements (Choi et al., 2008). Altogether, our population cohort analysis links dietary sugars to circulating purines and supports a shared mechanism between *Drosophila* and humans.

A combination of factors promotes the development of concretions in response to a high-sugar diet: (1) enhanced purine flux, increasing production of stone-precursor metabolites including uric acid, (2) the dehydration state, further concentrating their local levels within the organism, and (3) the acidification of the tubules and digestive tract, biochemically favoring uric acid stone formation (Barr, 1990; Johnson et al., 2018; Moe, 2006). Several human disorders are directly caused by uric acid accumulation, including gout (a form of inflammatory arthritis due to uric acid crystal deposition in the joints) and kidney stones (Barr, 1990; Moe, 2006). T2D is associated with an increased risk for uric acid kidney stones (Daudon et al., 2006), and consistent with the role of low pH in promoting uric acid crystallization, individuals with T2D exhibit urine acidification (pH < 5.5; Bell, 2012). Beyond the specific diseases of gout and kidney stones, elevated serum uric acid (hyperuricemia) has emerged from many clinical studies as a biomarker or risk factor for metabolic disease. Hyperuricemia was found to increase the prevalence of metabolic syndrome (Choi and Ford, 2007; Ford et al., 2007) and to predict the onset of T2D in several prospective studies (Bhole et al., 2010; Johnson et al., 2013b; Kodama et al., 2009; Niskanen et al., 2006). These observations raise the intriguing possibility that asymptomatic hyperuricemia (i.e., elevated serum uric acid without gout or kidney stones) may actually contribute to the progression of metabolic disease and may itself be worthy of therapeutic intervention (Johnson et al., 2013b; Levy and Cheetham, 2015). Hyperuricemia is also associated with cardiovascular disease and is a strong predictor of cardiovascular mortality (Anker et al., 2003; Kanbay et al., 2013; Niskanen et al., 2004; Zuo et al., 2016). Furthermore, hyperuricemia was identified as a predictor of mortality in acute care of hospitalized elderly patients (Breuer et al., 2017) and from several prospective studies as a risk factor for all-cause mortality (Niskanen et al., 2004; Zuo et al., 2016). Overall, this highlights that uric acid is associated with a range of metabolic and physiological disorders and potentially linked to human mortality as suggested by our study.

Here, we show that water imbalance plays an important role in mediating the pathology of both high-sugar and high-purine diets. Water is vital for all living systems, yet its importance is often overlooked in biological studies (Chaplin, 2006). For example, dehydration stress induces innate immunity in *Drosophila* through steroid signaling in the renal tubules (Zheng et al., 2018). Water dysregulation is also relevant in the context of metabolic disease. For instance, thirst is an early symptom of T2D (WHO, 2016), and low water intake is a predictor of hyperglycemia (Roussel et al., 2011). Hydration status is also known to affect kidney function and plasma uric acid levels in humans, and the geographical distribution of kidney stone prevalence is higher in hotter climates associated with dehydration (Chapman et al., 2019; Johnson et al., 2018; Moe, 2006; Sánchez-Lozada et al., 2019). Consistent with our findings in *Drosophila*, human epidemiological studies show positive effects of fluid intake on decreasing the risk of kidney stone formation (Curhan et al., 1998). Uric acid concentrations above $\sim$6–7 mg/dL are prone to crystallization (Barr, 1990), which, interestingly, is equivalent to the $\sim$70 ng/μL detected in the hemolymph of flies fed the high-sugar diet, showing that the levels are surprisingly well controlled and suggesting an evolutionarily conserved biochemical mechanism between distant organisms.

The XO inhibitor AP is widely prescribed to patients for the treatment of conditions associated with uric acid accumulation, including gout and kidney stones (Pacher et al., 2006). In our

study, we adopted a pharmacological approach to prevent uric acid formation by treating flies with AP. Importantly, despite AP treatment successfully clearing the tubules of uric acid stones, the accumulation of upstream metabolites in the purine degradation pathway (Bratty et al., 2011) was itself pathophysiological. Xanthine and hypoxanthine are also biochemically prone to form crystals (Miller et al., 2013), as we observed in the rectal ampulla, which may explain the incomplete rescue of survival upon AP treatment in our experiments. Consistent with our findings, genetic disruption of purine catabolism is detrimental: null mutants for XO, encoded by the *rosy* (*ry*) gene in *Drosophila*, develop xanthine tubule stones (Miller et al., 2013) and are short-lived (Shepherd et al., 1989). Therefore, although AP provided a partial rescue of the high-sugar-induced uric-acid-associated mortality, the blockage of purine catabolism was metabolically disruptive and ultimately detrimental to survival, suggesting a need for novel therapies and approaches, which could be tested for rescue of survival with the *Drosophila* model system.

Overall, our study highlights the purine catabolism pathway as a metabolic mediator of longevity, emphasizing the importance of diet and hydration as factors in promoting uric acid levels. Using *Drosophila* and a human population cohort, we provide a model to explain the pathophysiology of high-sugar diets, independently of obesity and insulin resistance. Our work paves the way for exploring the underappreciated role of both water and uric acid in the context of lifespan and provides a novel approach for discovering therapeutic strategies aimed at reducing high-sugar-induced mortality while maintaining the healthy regulation of purine catabolism.

Limitations of Study

The full relevance of our findings in *Drosophila* to sugar-induced pathophysiology in humans requires further studies in mammalian models. The human dietary information is based on self-reported intake from a food frequency questionnaire, which has inherent bias and refers to an annual average, whereas the serum metabolomics is performed on a single fasted blood sample. Our findings are from an aged cohort in Germany (average 59 years, SD 12.6 years); therefore, the analysis should be expanded to other populations to account for possible genetic and environmental factors. While the human data in our study correlate dietary sugar intake with renal function and serum purine levels, associations with human longevity still need to be explored.

STAR★METHODS

Detailed methods are provided in the online version of this paper and include the following:

- KEY RESOURCES TABLE
- LEAD CONTACT AND MATERIALS AVAILABILITY
- EXPERIMENTAL MODEL AND SUBJECT DETAILS
  - Fly Strains
  - Human Population Cohort
- METHOD DETAILS
  - Fly Media and Agar Tips
  - Fly Husbandry
  - Lifespan and Stress Assays
  - Fecundity Assays
  - Drinking and Feeding Assays
  - Freezing of Fly Samples for Molecular Assays
  - Whole Body Triglyceride Assay
  - Lipid Staining
  - Hemolymph Analysis
  - Fat Body Insulin and Glucose Assays
  - Protein Glycation and Carbonylation Assays
  - Gut Function Assays
  - Tubule and Rectal Ampulla Imaging
  - Uric Acid Assay
  - Tubule Secretion Assay
  - Metabolomics Analysis of Rectal Ampulla Stones
  - Human Blood Analysis and Metabolomics
  - Food Questionnaire and Molecular Diet Composition
  - Analysis of Variance
  - Linear Modeling
- QUANTIFICATION AND STATISTICAL ANALYSIS
  - Fly statistical analysis
  - Human Statistical Analysis
- DATA AND CODE AVAILABILITY

SUPPLEMENTAL INFORMATION

Supplemental Information can be found online at https://doi.org/10.1016/j.cmet.2020.02.016.

ACKNOWLEDGMENTS

Work in the group of H.M.C. is funded by the Medical Research Council, UK (MC-A654-5QB90). E.v.D. was supported by an Erasmus mobility studentship. J.J. was the recipient of a Commonwealth Split-site PhD Scholarship. C.K. acknowledges support by the DFG, Germany (CRC1182 “Metaorganisms” sub-project A1 and EXC2167 “Precision Medicine in Chronic Inflammation” sub-project RTF-VIII). We are grateful to members of the Cochemé lab and our colleagues in the groups of Irene Miguel-Aliaga and Susumu Hirabayashi for helpful discussion and input, especially Dafni Hadjieconomou and Chris Studd. We thank Linda Partridge, Cathy Slack, and the Bloomington Stock Center for fly lines. We acknowledge Paola Cognigni and Matthew Wayland for advice on excreta analysis with TURD software. We thank Filipe Cabreiro, Mike Murphy, Ivana Bjedov, and Jorge Ivan Castillo-Quan for valuable feedback.

AUTHOR CONTRIBUTIONS

H.M.C. conceived the project and supervised the fly work. E.v.D., L.A.G.v.L., E.d.S., J.J., C.L., A.J.V., A. Foley, M.B., and H.M.C. performed and analyzed fly experiments. J.B.M. and H.B.K. performed the fly metabolomics analysis. L.B. and G.M. analyzed the human dietary and metabolomics data, supervised by C.K. W.L., M.L., and A. Franke are responsible for overseeing the human cohort and biobank. H.M.C. wrote the manuscript with input from all authors.

DECLARATION OF INTERESTS

The authors declare no competing interests.

Received: August 19, 2019
Revised: January 29, 2020
Accepted: February 24, 2020
Published: March 19, 2020

REFERENCES

Anker, S.D., Doehner, W., Rauchhaus, M., Sharma, R., Francis, D., Knosalla, C., Davos, C.H., Cicoira, M., Shamim, W., Kemp, M., et al. (2003). Uric acid

and survival in chronic heart failure: validation and application in metabolic, functional, and hemodynamic staging. Circulation *107*, 1991–1997.

Baker, K.D., and Thummel, C.S. (2007). Diabetic larvae and obese flies-emerging studies of metabolism in *Drosophila*. Cell Metab. *6*, 257–266.

Barr, W.G. (1990). Uric Acid. In Clinical Methods: The History, Physical, and Laboratory Examinations, H.K. Walker, W.D. Hall, and J.W. Hurst, eds. (Butterworths), pp. 770–772.

Bass, T.M., Grandison, R.C., Wong, R., Martinez, P., Partridge, L., and Piper, M.D. (2007). Optimization of dietary restriction protocols in *Drosophila*. J. Gerontol. A Biol. Sci. Med. Sci. *62*, 1071–1081.

Bell, D.S. (2012). Beware the low urine pH–the major cause of the increased prevalence of nephrolithiasis in the patient with type 2 diabetes. Diabetes Obes. Metab. *14*, 299–303.

Bellomo, G., Venanzi, S., Verdura, C., Saronio, P., Esposito, A., and Timio, M. (2010). Association of uric acid with change in kidney function in healthy normotensive individuals. Am. J. Kidney Dis. *56*, 264–272.

Benjamini, Y., and Hochberg, Y. (1995). Controlling the False Discovery Rate - a Practical and Powerful Approach to Multiple Testing. Journal of the Royal Statistical Society. Series B *57*, 289–300.

Bhole, V., Choi, J.W., Kim, S.W., de Vera, M., and Choi, H. (2010). Serum uric acid levels and the risk of type 2 diabetes: a prospective study. Am. J. Med. *123*, 957–961.

Brandstetter, B.R., Korfmann, A., Kroke, A., Becker, N., Schulze, M.B., and Boeing, H. (1999). Dietary habits in the German EPIC cohorts: food group intake estimated with the food frequency questionnaire. European Investigation into Cancer and Nutrition. Ann. Nutr. Metab. *43*, 246–257.

Bratty, M.A., Hobani, Y., Dow, J.A.T., and Watson, D.G. (2011). Metabolomic profiling of the effects of allopurinol on *Drosophila melanogaster*. Metabolomics *7*, 542–548.

Breuer, G.S., Schwartz, Y., Freier-Dror, Y., and Nesher, G. (2017). Uric acid level as predictor of mortality in the acute care setting of advanced age population. Eur. J. Intern. Med. *44*, 74–76.

Broughton, S.J., Piper, M.D., Ikeya, T., Bass, T.M., Jacobson, J., Driege, Y., Martinez, P., Hafen, E., Withers, D.J., Leevers, S.J., and Partridge, L. (2005). Longer lifespan, altered metabolism, and stress resistance in *Drosophila* from ablation of cells making insulin-like ligands. Proc. Natl. Acad. Sci. USA *102*, 3105–3110.

Chaplin, M. (2006). Do we underestimate the importance of water in cell biology? Nat. Rev. Mol. Cell Biol. *7*, 861–866.

Chapman, C.L., Johnson, B.D., Sackett, J.R., Parker, M.D., and Schlader, Z.J. (2019). Soft drink consumption during and following exercise in the heat elevates biomarkers of acute kidney injury. Am. J. Physiol. Regul. Integr. Comp. Physiol. *316*, R189–R198.

Chatterjee, S., Khunti, K., and Davies, M.J. (2017). Type 2 diabetes. Lancet *389*, 2239–2251.

Choi, H.K., and Ford, E.S. (2007). Prevalence of the metabolic syndrome in individuals with hyperuricemia. Am. J. Med. *120*, 442–447.

Choi, J.W., Ford, E.S., Gao, X., and Choi, H.K. (2008). Sugar-sweetened soft drinks, diet soft drinks, and serum uric acid level: the Third National Health and Nutrition Examination Survey. Arthritis Rheum. *59*, 109–116.

Cirulli, E.T., Guo, L., Leon Swisher, C., Shah, N., Huang, L., Napier, L.A., Kirkness, E.F., Spector, T.D., Caskey, C.T., Thorens, B., et al. (2019). Profound Perturbation of the Metabolome in Obesity Is Associated with Health Risk. Cell Metab. *29*, 488–500.e2.

Cognigni, P., Bailey, A.P., and Miguel-Aliaga, I. (2011). Enteric neurons and systemic signals couple nutritional and reproductive status with intestinal homeostasis. Cell Metab. *13*, 92–104.

Curhan, G.C., Willett, W.C., Speizer, F.E., and Stampfer, M.J. (1998). Beverage use and risk for kidney stones in women. Ann. Intern. Med. *128*, 534–540.

Daudon, M., Traxer, O., Conort, P., Lacour, B., and Jungers, P. (2006). Type 2 diabetes increases the risk for uric acid stones. J. Am. Soc. Nephrol. *17*, 2026–2033.

DeFronzo, R.A., Ferrannini, E., Groop, L., Henry, R.R., Herman, W.H., Holst, J.J., Hu, F.B., Kahn, C.R., Raz, I., Shulman, G.I., et al. (2015). Type 2 diabetes mellitus. Nat. Rev. Dis. Primers *1*, 15019.

Dow, J.A., and Romero, M.F. (2010). *Drosophila* provides rapid modeling of renal development, function, and disease. Am. J. Physiol. Renal Physiol. *299*, F1237–F1244.

Dow, J.A., Maddrell, S.H., Görtz, A., Skaer, N.J., Brogan, S., and Kaiser, K. (1994). The malpighian tubules of *Drosophila melanogaster*: a novel phenotype for studies of fluid secretion and its control. J. Exp. Biol. *197*, 421–428.

Fanson, B.G., Yap, S., and Taylor, P.W. (2012). Geometry of compensatory feeding and water consumption in *Drosophila melanogaster*. J. Exp. Biol. *215*, 766–773.

Flatt, T. (2011). Survival costs of reproduction in *Drosophila*. Exp. Gerontol. *46*, 369–375.

Flegal, K.M., Kit, B.K., Orpana, H., and Graubard, B.I. (2013). Association of all-cause mortality with overweight and obesity using standard body mass index categories: a systematic review and meta-analysis. JAMA *309*, 71–82.

Ford, E.S., Li, C., Cook, S., and Choi, H.K. (2007). Serum concentrations of uric acid and the metabolic syndrome among US children and adolescents. Circulation *115*, 2526–2532.

Garrido, D., Rubin, T., Poidevin, M., Maroni, B., Le Rouzic, A., Parvy, J.P., and Montagne, J. (2015). Fatty acid synthase cooperates with glyoxalase 1 to protect against sugar toxicity. PLoS Genet. *11*, e1004995.

Gems, D., and Partridge, L. (2008). Stress-response hormesis and aging: "that which does not kill us makes us stronger". Cell Metab. *7*, 200–203.

Ghimire, S., Terhzaz, S., Cabrero, P., Romero, M.F., Davies, S.A., and Dow, J.A.T. (2019). Targeted renal knockdown of Na^+/H^+ exchanger regulatory factor *Sip1* produces uric acid nephrolithiasis in *Drosophila*. Am. J. Physiol. Renal Physiol. *317*, F930–F940.

Ghosh, A.C., and O'Connor, M.B. (2014). Systemic Activin signaling independently regulates sugar homeostasis, cellular metabolism, and pH balance in *Drosophila melanogaster*. Proc. Natl. Acad. Sci. USA *111*, 5729–5734.

Gibbs, A.G., and Markow, T.A. (2001). Effects of age on water balance in *Drosophila* species. Physiol. Biochem. Zool. *74*, 520–530.

Goh, S.Y., and Cooper, M.E. (2008). Clinical review: The role of advanced glycation end products in progression and complications of diabetes. J. Clin. Endocrinol. Metab. *93*, 1143–1152.

Grandison, R.C., Piper, M.D., and Partridge, L. (2009a). Amino-acid imbalance explains extension of lifespan by dietary restriction in *Drosophila*. Nature *462*, 1061–1064.

Grandison, R.C., Wong, R., Bass, T.M., Partridge, L., and Piper, M.D. (2009b). Effect of a standardised dietary restriction protocol on multiple laboratory strains of *Drosophila melanogaster*. PLoS ONE *4*, e4067.

Hughes, V. (2013). The big fat truth. Nature *497*, 428–430.

Ikeya, T., Broughton, S., Alic, N., Grandison, R., and Partridge, L. (2009). The endosymbiont Wolbachia increases insulin/IGF-like signalling in *Drosophila*. Proc. Biol. Sci. *276*, 3799–3807.

Itskov, P.M., Moreira, J.M., Vinnik, E., Lopes, G., Safarik, S., Dickinson, M.H., and Ribeiro, C. (2014). Automated monitoring and quantitative analysis of feeding behaviour in *Drosophila*. Nat. Commun. *5*, 4560.

Ja, W.W., Carvalho, G.B., Mak, E.M., de la Rosa, N.N., Fang, A.Y., Liong, J.C., Brummel, T., and Benzer, S. (2007). Prandiology of *Drosophila* and the CAFE assay. Proc. Natl. Acad. Sci. USA *104*, 8253–8256.

Ja, W.W., Carvalho, G.B., Zid, B.M., Mak, E.M., Brummel, T., and Benzer, S. (2009). Water- and nutrient-dependent effects of dietary restriction on *Drosophila* lifespan. Proc. Natl. Acad. Sci. USA *106*, 18633–18637.

Jacobson, J., Lambert, A.J., Portero-Otín, M., Pamplona, R., Magwere, T., Miwa, S., Driege, Y., Brand, M.D., and Partridge, L. (2010). Biomarkers of aging in *Drosophila*. Aging Cell *9*, 466–477.

Johnson, R.J., Nakagawa, T., Jalal, D., Sánchez-Lozada, L.G., Kang, D.H., and Ritz, E. (2013a). Uric acid and chronic kidney disease: which is chasing which? Nephrol. Dial. Transplant. *28*, 2221–2228.

Johnson, R.J., Nakagawa, T., Sanchez-Lozada, L.G., Shafiu, M., Sundaram, S., Le, M., Ishimoto, T., Sautin, Y.Y., and Lanaspa, M.A. (2013b). Sugar, uric acid, and the etiology of diabetes and obesity. Diabetes *62*, 3307–3315.

Johnson, R.J., Perez-Pozo, S.E., Lillo, J.L., Grases, F., Schold, J.D., Kuwabara, M., Sato, Y., Hernando, A.A., Garcia, G., Jensen, T., et al. (2018). Fructose increases risk for kidney stones: potential role in metabolic syndrome and heat stress. BMC Nephrol. *19*, 315.

Kanbay, M., Segal, M., Afsar, B., Kang, D.H., Rodriguez-Iturbe, B., and Johnson, R.J. (2013). The role of uric acid in the pathogenesis of human cardiovascular disease. Heart *99*, 759–766.

Koch, M., Borggrefe, J., Barbaresko, J., Groth, G., Jacobs, G., Siegert, S., Lieb, W., Müller, M.J., Bosy-Westphal, A., Heller, M., and Nöthlings, U. (2014). Dietary patterns associated with magnetic resonance imaging-determined liver fat content in a general population study. Am. J. Clin. Nutr. *99*, 369–377.

Koch, M., Freitag-Wolf, S., Schlesinger, S., Borggrefe, J., Hov, J.R., Jensen, M.K., Pick, J., Markus, M.R.P., Höpfner, T., Jacobs, G., et al. (2017). Serum metabolomic profiling highlights pathways associated with liver fat content in a general population sample. Eur. J. Clin. Nutr. *71*, 995–1001.

Kodama, S., Saito, K., Yachi, Y., Asumi, M., Sugawara, A., Totsuka, K., Saito, A., and Sone, H. (2009). Association between serum uric acid and development of type 2 diabetes. Diabetes Care *32*, 1737–1742.

Lang, S., Hilsabeck, T.A., Wilson, K.A., Sharma, A., Bose, N., Brackman, D.J., Beck, J.N., Chen, L., Watson, M.A., Killilea, D.W., et al. (2019). A conserved role of the insulin-like signaling pathway in diet-dependent uric acid pathologies in *Drosophila melanogaster*. PLoS Genet. *15*, e1008318.

Leader, D.P., Krause, S.A., Pandit, A., Davies, S.A., and Dow, J.A.T. (2018). FlyAtlas 2: a new version of the *Drosophila melanogaster* expression atlas with RNA-Seq, miRNA-Seq and sex-specific data. Nucleic Acids Res. *46* (D1), D809–D815.

Levey, A.S., Coresh, J., Greene, T., Stevens, L.A., Zhang, Y.L., Hendriksen, S., Kusek, J.W., and Van Lente, F.; Chronic Kidney Disease Epidemiology Collaboration (2006). Using standardized serum creatinine values in the modification of diet in renal disease study equation for estimating glomerular filtration rate. Ann. Intern. Med. *145*, 247–254.

Levy, G., and Cheetham, T.C. (2015). Is It Time to Start Treating Asymptomatic Hyperuricemia? Am. J. Kidney Dis. *66*, 933–935.

Lushchak, O.V., Gospodaryov, D.V., Rovenko, B.M., Yurkevych, I.S., Perkhulyn, N.V., and Lushchak, V.I. (2014). Specific dietary carbohydrates differentially influence the life span and fecundity of *Drosophila melanogaster*. J. Gerontol. A Biol. Sci. Med. Sci. *69*, 3–12.

Lustig, R.H., Schmidt, L.A., and Brindis, C.D. (2012). Public health: The toxic truth about sugar. Nature *482*, 27–29.

Mattila, J., and Hietakangas, V. (2017). Regulation of Carbohydrate Energy Metabolism in *Drosophila melanogaster*. Genetics *207*, 1231–1253.

Miguel-Aliaga, I., Jasper, H., and Lemaitre, B. (2018). Anatomy and Physiology of the Digestive Tract of *Drosophila melanogaster*. Genetics *210*, 357–396.

Miller, J., Chi, T., Kapahi, P., Kahn, A.J., Kim, M.S., Hirata, T., Romero, M.F., Dow, J.A., and Stoller, M.L. (2013). *Drosophila melanogaster* as an emerging translational model of human nephrolithiasis. J. Urol. *190*, 1648–1656.

Moe, O.W. (2006). Kidney stones: pathophysiology and medical management. Lancet *367*, 333–344.

Morris, S.N., Coogan, C., Chamseddin, K., Fernandez-Kim, S.O., Kolli, S., Keller, J.N., and Bauer, J.H. (2012). Development of diet-induced insulin resistance in adult *Drosophila melanogaster*. Biochim. Biophys. Acta *1822*, 1230–1237.

Müller, N., Schulte, D.M., Türk, K., Freitag-Wolf, S., Hampe, J., Zeuner, R., Schröder, J.O., Gouni-Berthold, I., Berthold, H.K., Krone, W., et al. (2015). IL-6 blockade by monoclonal antibodies inhibits apolipoprotein (a) expression and lipoprotein (a) synthesis in humans. J. Lipid Res. *56*, 1034–1042.

Musselman, L.P., Fink, J.L., Narzinski, K., Ramachandran, P.V., Hathiramani, S.S., Cagan, R.L., and Baranski, T.J. (2011). A high-sugar diet produces obesity and insulin resistance in wild-type *Drosophila*. Dis. Model. Mech. *4*, 842–849.

Na, J., Musselman, L.P., Pendse, J., Baranski, T.J., Bodmer, R., Ocorr, K., and Cagan, R. (2013). A *Drosophila* model of high sugar diet-induced cardiomyopathy. PLoS Genet. *9*, e1003175.

Niskanen, L.K., Laaksonen, D.E., Nyyssönen, K., Alfthan, G., Lakka, H.M., Lakka, T.A., and Salonen, J.T. (2004). Uric acid level as a risk factor for cardiovascular and all-cause mortality in middle-aged men: a prospective cohort study. Arch. Intern. Med. *164*, 1546–1551.

Niskanen, L., Laaksonen, D.E., Lindström, J., Eriksson, J.G., Keinänen-Kiukaanniemi, S., Ilanne-Parikka, P., Aunola, S., Hämäläinen, H., Tuomilehto, J., and Uusitupa, M. (2006). Serum uric acid as a harbinger of metabolic outcome in subjects with impaired glucose tolerance: the Finnish Diabetes Prevention Study. Diabetes Care *29*, 709–711.

Ortega, F.B., Ruiz, J.R., Labayen, I., Lavie, C.J., and Blair, S.N. (2018). The Fat but Fit paradox: what we know and don't know about it. Br. J. Sports Med. *52*, 151–153.

Oudes, A.J., Herr, C.M., Olsen, Y., and Fleming, J.E. (1998). Age-dependent accumulation of advanced glycation end-products in adult *Drosophila melanogaster*. Mech. Ageing Dev. *100*, 221–229.

Owusu-Ansah, E., and Perrimon, N. (2014). Modeling metabolic homeostasis and nutrient sensing in *Drosophila*: implications for aging and metabolic diseases. Dis. Model. Mech. *7*, 343–350.

Pacher, P., Nivorozhkin, A., and Szabó, C. (2006). Therapeutic effects of xanthine oxidase inhibitors: renaissance half a century after the discovery of allopurinol. Pharmacol. Rev. *58*, 87–114.

Padmanabha, D., and Baker, K.D. (2014). *Drosophila* gains traction as a repurposed tool to investigate metabolism. Trends Endocrinol. Metab. *25*, 518–527.

Partridge, L., Alic, N., Bjedov, I., and Piper, M.D. (2011). Ageing in *Drosophila*: the role of the insulin/Igf and TOR signalling network. Exp. Gerontol. *46*, 376–381.

Piper, M.D., Wong, R., Grandison, R.C., Bass, T.M., Martinez, P.M., and Partridge, L. (2010). Water-independent effects of dietary restriction in *Drosophila*. Proc. Natl. Acad. Sci. USA *107*, E54–E56, author reply E57.

Pryor, R., Norvaisas, P., Marinos, G., Best, L., Thingholm, L.B., Quintaneiro, L.M., De Haes, W., Esser, D., Waschina, S., Lujan, C., et al. (2019). Host-Microbe-Drug-Nutrient Screen Identifies Bacterial Effectors of Metformin Therapy. Cell *178*, 1299–1312.e29.

Rera, M., Clark, R.I., and Walker, D.W. (2012). Intestinal barrier dysfunction links metabolic and inflammatory markers of aging to death in *Drosophila*. Proc. Natl. Acad. Sci. USA *109*, 21528–21533.

Riera, C.E., Merkwirth, C., De Magalhaes Filho, C.D., and Dillin, A. (2016). Signaling Networks Determining Life Span. Annu. Rev. Biochem. *85*, 35–64.

Roussel, R., Fezeu, L., Bouby, N., Balkau, B., Lantieri, O., Alhenc-Gelas, F., Marre, M., and Bankir, L.; D.E.S.I.R. Study Group (2011). Low water intake and risk for new-onset hyperglycemia. Diabetes Care *34*, 2551–2554.

Rovenko, B.M., Kubrak, O.I., Gospodaryov, D.V., Perkhulyn, N.V., Yurkevych, I.S., Sanz, A., Lushchak, O.V., and Lushchak, V.I. (2015). High sucrose consumption promotes obesity whereas its low consumption induces oxidative stress in *Drosophila melanogaster*. J. Insect Physiol. *79*, 42–54.

Sánchez-Lozada, L.G., Roncal-Jimenez, C.A., García-Arroyo, F.E., Jensen, T., Lanaspa, M.A., and Johnson, R.J. (2019). The perils of rehydrating with soft drinks following heat and exercise. Am. J. Physiol. Regul. Integr. Comp. Physiol. *316*, R187–R188.

Schellinger, J.N., and Rodan, A.R. (2015). Use of the Ramsay Assay to Measure Fluid Secretion and Ion Flux Rates in the *Drosophila melanogaster* Malpighian Tubule. J. Vis. Exp. *105*, 53144.

Schindelin, J., Arganda-Carreras, I., Frise, E., Kaynig, V., Longair, M., Pietzsch, T., Preibisch, S., Rueden, C., Saalfeld, S., Schmid, B., et al. (2012). Fiji: an open-source platform for biological-image analysis. Nat. Methods *9*, 676–682.

Shell, B.C., Schmitt, R.E., Lee, K.M., Johnson, J.C., Chung, B.Y., Pletcher, S.D., and Grotewiel, M. (2018). Measurement of solid food intake in *Drosophila* via consumption-excretion of a dye tracer. Sci. Rep. *8*, 11536.

Shepherd, J.C., Walldorf, U., Hug, P., and Gehring, W.J. (1989). Fruit flies with additional expression of the elongation factor EF-1 alpha live longer. Proc. Natl. Acad. Sci. USA *86*, 7520–7521.

Skorupa, D.A., Dervisefendic, A., Zwiener, J., and Pletcher, S.D. (2008). Dietary composition specifies consumption, obesity, and lifespan in *Drosophila melanogaster*. Aging Cell *7*, 478–490.

Slack, C., Giannakou, M.E., Foley, A., Goss, M., and Partridge, L. (2011). dFOXO-independent effects of reduced insulin-like signaling in *Drosophila*. Aging Cell *10*, 735–748.

Teleman, A.A. (2009). Molecular mechanisms of metabolic regulation by insulin in *Drosophila*. Biochem. J. *425*, 13–26.

Toivonen, J.M., Walker, G.A., Martinez-Diaz, P., Bjedov, I., Driege, Y., Jacobs, H.T., Gems, D., and Partridge, L. (2007). No influence of Indy on lifespan in *Drosophila* after correction for genetic and cytoplasmic background effects. PLoS Genet. *3*, e95.

vanEngelsdorp, D., Traynor, K.S., Andree, M., Lichtenberg, E.M., Chen, Y., Saegerman, C., and Cox-Foster, D.L. (2017). Colony Collapse Disorder (CCD) and bee age impact honey bee pathophysiology. PLoS ONE *12*, e0179535.

Wayland, M.T., Defaye, A., Rocha, J., Jayaram, S.A., Royet, J., Miguel-Aliaga, I., Leulier, F., and Cognigni, P. (2014). Spotting the differences: probing host/microbiota interactions with a dedicated software tool for the analysis of faecal outputs in *Drosophila*. J. Insect Physiol. *69*, 126–135.

WHO (2016). Global Report on Diabetes (World Health Organization).

WHO (2020). "Obesity and Overweight" Factsheet (World Health Organization), https://www.who.int/news-room/fact-sheets/detail/obesity-and-overweight.

Wong, R., Piper, M.D., Wertheim, B., and Partridge, L. (2009). Quantification of food intake in *Drosophila*. PLoS One *4*, e6063.

Zheng, W., Rus, F., Hernandez, A., Kang, P., Goldman, W., Silverman, N., and Tatar, M. (2018). Dehydration triggers ecdysone-mediated recognition-protein priming and elevated anti-bacterial immune responses in *Drosophila* Malpighian tubule renal cells. BMC Biol. *16*, 60.

Zuo, T., Liu, X., Jiang, L., Mao, S., Yin, X., and Guo, L. (2016). Hyperuricemia and coronary heart disease mortality: a meta-analysis of prospective cohort studies. BMC Cardiovasc. Disord. *16*, 207.

STAR★METHODS

KEY RESOURCES TABLE

REAGENT or RESOURCE	SOURCE	IDENTIFIER
Antibodies		
Rabbit polyclonal anti-*Drosophila* phospho-AKT (Ser505), dil: 1/1000	Cell Signaling Technology	Cat# 4054; RRID:AB_331414
Rabbit polyclonal anti-total AKT, dil: 1/1000	Cell Signaling Technology	Cat# 9272; RRID:AB_329827
Mouse monoclonal anti-β-actin, dil: 1/1000	Abcam	Cat# ab8224; RRID:AB_449644
Goat polyclonal anti-AGE (advanced glycated end-products), dil:1/2000	Millipore	Cat# AB9890; RRID:AB_805248
Chemicals, Peptides, and Recombinant Proteins		
Sucrose (granulated sugar)	Tate & Lyle	N/A
Brewer's yeast	MP Biomedicals	Cat# 903312
Agar	Sigma-Aldrich	Cat# A7002
Nipagin (Methyl 4-hydroxybenzoate)	Sigma-Aldrich	Cat# H5501
Propionic acid	Sigma-Aldrich	Cat# P1386
D-Fructose	Sigma-Aldrich	Cat# F0127
D-Glucose	Sigma-Aldrich	Cat# G8270
Allopurinol	Sigma-Aldrich	Cat# A8003
Adenine	Sigma-Aldrich	Cat# A8626
Guanine	Sigma-Aldrich	Cat# G11950
Paraquat	Sigma-Aldrich	Cat# 856177
Brilliant Blue FCF food dye	Town End (Leeds) Plc, UK	https://www.dyes.co.uk/water-soluble-powder-colours.html
Nile Red stain	Thermo Fisher Scientific (Invitrogen)	Cat# N1142
Trehalase	Megazyme	Cat# E-TREH
Insulin	Sigma-Aldrich	Cat# I9278
2-NBDG (2-(*N*-(7-nitrobenz-2-oxa-1,3-diazol-4-yl)amino)-2-deoxyglucose)	Thermo Fisher Scientific (Invitrogen)	Cat# N13195
Mineral oil heavy	Sigma-Aldrich	Cat# 330760
Bromophenol blue	Sigma-Aldrich	Cat# B5525
Bromocresol purple	Sigma-Aldrich	Cat# B5880
Pyranine	Thermo Fisher Scientific (Invitrogen)	Cat# H348
$^{13}C_5$-Hypoxanthine	Cambridge Isotope Laboratories	Cat# CLM-8042
Uric acid	Sigma-Aldrich	Cat# U2625
Allantoin	Sigma-Aldrich	Cat# 05670
Xanthine	Sigma-Aldrich	Cat# X0626
Hypoxanthine	Sigma-Aldrich	Cat# H9377
Critical Commercial Assays		
OxyBlot Protein Detection Kit	Millipore	Cat# S7150
QuantiChrom Uric Acid Assay Kit	BioAssay Systems	Cat# DIUA-250
Deposited Data		
Northern German cohort (metabolome, clinical metadata and nutritional questionnaire)	This study	Available from the PopGen biobank (https://www.uksh.de/p2n/Information+for+Researchers.html)
Experimental Models: Organisms/Strains		
D. melanogaster: *white Dahomey* (w^{Dah})	Linda Partridge (Broughton et al., 2005)	N/A
D. melanogaster: *Dahomey*	Linda Partridge	N/A
D. melanogaster: w^{Dah} *Wolbachia* negative	Linda Partridge (Toivonen et al., 2007)	N/A

(*Continued on next page*)

Continued		
REAGENT or RESOURCE	SOURCE	IDENTIFIER
D. melanogaster: w^{1118}	Linda Partridge	N/A
D. melanogaster: ovo^{D1} v^{24}/C(1)DX, y^1 w^1 f^1	Bloomington *Drosophila* Stock Center	RRID:BDSC_1309
D. melanogaster: $foxo^{\Delta 94}$/TM6B, Tb^1	Slack et al., 2011	RRID:BDSC_42220
Software and Algorithms		
TURD (v0.8)	Wayland et al., 2014	http://the-ultimate-reader-of-dung.sourceforge.net
GraphPad Prism 8	GraphPad Software	https://www.graphpad.com/scientific-software/prism/
FIJI (ImageJ v2.0.0)	Schindelin et al., 2012	https://fiji.sc
Image Lab (v5.2.1)	BioRad	https://www.bio-rad.com
Xcalibur software (v4.1)	Thermo Fisher Scientific	https://www.thermofisher.com/
R (v3.6.1)	R Core Team	https://www.r-project.org
Vegan (v2.5-6)	https://cran.r-project.org/package=vegan	N/A
RColorBrewer (v1.1-2)	https://cran.r-project.org/package=RColorBrewer	N/A
Other		
Triglyceride Infinity Reagent	Thermo Fisher Scientific	Cat# TR22421
Glucose Infinity Reagent	Thermo Fisher Scientific	Cat# TR15421
Schneider's *Drosophila* medium	Thermo Fisher Scientific (GIBCO)	Cat# 21720024
Laemmli buffer	BioRad	Cat# 1610737
BCA Protein Assay Kit	Thermo Fisher Scientific (Pierce)	Cat# 23225

LEAD CONTACT AND MATERIALS AVAILABILITY

Further information and requests for resources and reagents should be directed and will be fulfilled by the Lead Contact, Helena Cochemé (helena.cocheme@lms.mrc.ac.uk). This study did not generate new unique reagents.

EXPERIMENTAL MODEL AND SUBJECT DETAILS

Fly Strains

For the majority of experiments, the *white Dahomey* (w^{Dah}) strain of *Drosophila melanogaster* was used as the outbred WT background (Broughton et al., 2005). Additional experiments were performed in the parental *Dahomey* strain, a w^{Dah} strain cured from *Wolbachia* infection (Toivonen et al., 2007), and the isogenic control line w^{1118} (all kindly received from Linda Partridge, UCL). The mutant lines $foxo^{\Delta 94}$ (Slack et al., 2011) and ovo^{D1} (Bloomington, #1309) were backcrossed into the w^{Dah} background for at least 10 generations. Assays were mostly performed on mated females, unless otherwise stated. All experiments were performed at 25°C, 65% humidity on a 12 h light:12 h dark cycle.

Human Population Cohort

We analyzed data from the German population cohort FoCus (Müller et al., 2015), excluding individuals with known diagnosis of inflammatory bowel disease and syndrome, chronic diarrhea, kidney disease, or any type of diabetes. Further stratification was performed based on fasting plasma glucose levels, limited to a maximum of 125 mg/dL, considered as the borderline value between pre-diabetic and diabetic status. We determined the estimated glomerular filtration rate (eGFR) from serum creatinine levels using the formula below (Levey et al., 2006), and excluded participants with eGFR < 60 mL/min per 1.73 m^2 (i.e., mild to moderate loss of kidney function).

$$eGFR\left[\frac{\frac{mL}{min}}{1.73m^2}\right] = 186 \cdot \frac{creatinine\left[\frac{\mu mol}{L}\right]}{88.4}^{-1.154} \cdot age[years]^{-0.203} \cdot sex[female = 0.742; male = 1]$$

We also excluded any individuals on anti-gout medication. Our remaining participant count was n = 650. Besides diagnostic and medication usage information, clinical parameters such as BMI and age were recorded for each participant in the cohort,

summarized in Table S5. The study was approved by the ethical review board of the Medical Faculty of Kiel University (Kiel, Germany). Written informed consent was obtained from all study participants (Koch et al., 2017).

METHOD DETAILS

Fly Media and Agar Tips

Flies were raised on standard sugar-yeast-agar medium (5%S) consisting of: 5% w/v sucrose (granulated sugar, Tate & Lyle), 10% w/v Brewer's yeast (#903312, MP Biomedicals), 1.5% w/v agar (Sigma A7002), supplemented with nipagin (Sigma H5501; 30 mL/L of 10% w/v nipagin in 95% EtOH) and propionic acid (0.3% v/v; Sigma P1386) as preservatives, added once the food had cooled down to < 60°C (Grandison et al., 2009b). For high-sugar food, the sugar content was increased 4-fold to 20% w/v sucrose (20%S). For some experiments, the sugar content was increased 6-fold to 30% w/v sucrose (30%S) or 8-fold to 40% w/v sucrose (40%S). For low-sugar food, the sugar content was deceased to 0%, 1% and 2.5% w/v sucrose (0%S, 1%S and 2.5%S respectively). For selected experiments, the 5%S diet was supplemented with a 15% w/v excess of D-fructose (Sigma F0127) or D-glucose (Sigma G8270). Note that the protein source (yeast) was maintained constant throughout. For drug treatments, the food was supplemented with 10 μM, 100 μM or 1 mM allopurinol (Sigma A8003). For the high-purine diet, the 5%S food was supplemented with 10 mM purine, consisting of 5 mM adenine (Sigma A8626) and 5 mM guanine (Sigma G11950). See Table S1 for all media recipes.

To supply water to the flies, 20 μL filter tips (Starlab) containing 1.5% (w/v) agar were inserted into the food vials. These + H_2O tips were filled to bulging with agar to avoid them drying out (also helped by the lower position of the filter within these 20 μL tips, allowing for a larger volume of agar). As controls, empty 100 μL filter tips (Starlab) were used (the higher position of the filter prevented flies from being stuck in narrower regions of the tip). ~6 mL of food was dispensed into narrow plastic vials (25 × 95 mm; #32-109, FlyStuff, Genesee Scientific). This depth (~2 cm) ensured that the tips were sufficiently secured when inserted into the food and not dislodged during transfer of the flies. Vials were incubated horizontally, with the tip resting at the base (see Figure 1A).

Fly Husbandry

For all experiments, eggs were collected over a < 12 h period to ensure a synchronous population and reared at constant density (~250 eggs per 200 mL bottle) on standard 5%S food (Bass et al., 2007). Eclosing adults of a defined age were kept as a mixed population for ~48 h on standard 5%S to allow maturing and mating, then separated into females and males under mild CO_2 anesthesia, and maintained as separate sexes in vials from then onward. The different diets ± H_2O were initiated at this stage (d2 of adulthood). Flies (typically 15 or 20 flies per vial) were transferred to fresh food every 2-3 days without gassing.

Lifespan and Stress Assays

Lifespan assays were set up as above, with deaths and censors scored every 2-3 days. For desiccation stress, flies were pre-treated on 5%S or 20%S ± H_2O for 28 days, then transferred into empty vials stored inside a container with desiccant. For high salt stress, flies were pre-treated on 5%S or 20%S ± H_2O for 7 days, then transferred onto medium consisting of 500 mM NaCl in 5% w/v sucrose, 1.5% w/v agar. For oxidative stress, flies were pre-treated on 5%S or 20%S ± H_2O for 7 days, then transferred onto 20 mM paraquat (Sigma 856177) in 5%S food. For starvation stress, flies were pre-treated on 5%S or 20%S ± H_2O for 7 days, then transferred onto vials of 1.5% w/v agar. Flies were incubated at 25°C, and deaths were scored several times daily, as appropriate. Typically, a total of n ~100-200 flies were set up per condition for all survival experiments.

Fecundity Assays

Fecundity was assayed as the number of eggs laid per female in each vial after a 24 h period at the indicated age during a lifespan experiment counted under a stereomicroscope (typically, n = 10 vials per condition, each with 15 females per vial). To test the effect of essential amino acid (EAA) supplementation on fecundity, an EAA cocktail was prepared into 1.5% w/v agar tips, and provided with the food. The final EAA concentrations were as follows: Arg, 425 μg/mL; His, 210 μg/mL; Ile, 340 μg/mL; Leu, 475 μg/mL; Lys, 515 μg/mL; Met, 100 μg/mL; Phe, 260 μg/mL; Thr, 365 μg/mL; Trp, 90 μg/mL; Val, 400 μg/mL (Grandison et al., 2009a).

Drinking and Feeding Assays

Drinking was assayed using the automated FlyPAD system (Itskov et al., 2014). Individual flies pre-treated on 5%S or 20%S ± H_2O were transferred without anesthesia into FlyPAD arenas with a source of agar, and the number of sips quantified over time. Alternatively, drinking was assayed by providing flies an agar tip supplemented with 2.5% w/v blue food dye (Brilliant Blue FCF) for ~1.5 h (until the first appearance of blue excreta). Flies were then snap frozen, and the amount of blue dye was quantified spectrophotometrically at 630 nm from clarified homogenates (Wong et al., 2009). Finally, an adaptation of the Capillary Feeder (CAFE) assay (Ja et al., 2007) was used. A 5 μL glass micropipette (ringcaps #9600105, Hirschmann) containing water, supplemented with 0.1 mg/mL blue dye to aid visualization, was inserted at the side of the vial, secured by the cotton plug. The level of the water was marked, and the vials were incubated in a humidified sealed container at 25°C. The volume of water decrease was then recorded after 24 h. Empty vials without flies were also included to control for evaporation.

To assay feeding rates, flies (n ~ 10 vials per condition, each with n = 5 flies) were placed onto observation racks at 25°C, left undisturbed for ~24 h, then the proportion of feeding flies was assayed blinded based on proboscis extension every 2-3 min over 90 min (Wong et al., 2009). Alternatively, feeding was assayed based on the consumption/excretion of dye-containing food as

described (Shell et al., 2018). Media were supplemented with 1% w/v blue food dye and dispensed into plastic caps (#FCS1NA1, MOCAP) that fit wide plastic vials (28.5 × 95 mm; #32-110, FlyStuff, Genesee Scientific). An agar tip was inserted into the food-filled cap for water supplementation. Flies were incubated at 25°C for 24 h, which accounts for any possible circadian effects on feeding, then the food caps and flies were removed, and the insides of the vials were washed with 3 mL H_2O to resuspend all blue-dyed excreta. The absorbance at 630 nm was measured in a plate reader (FLUOstar Omega, BMG Labtech), and the total volume of blue-dyed food excreted per fly over 24 h was calculated as an indicator of overall feeding (Shell et al., 2018).

Freezing of Fly Samples for Molecular Assays

Live flies were rapidly transferred to microtubes pre-chilled on dry ice via a small plastic funnel and snap frozen in liquid nitrogen, then stored at –80°C until required. Flies were always frozen at approximately the same time of day to minimize any circadian variation.

Whole Body Triglyceride Assay

To measure whole body triglyceride (TAG) levels, frozen flies (n = 4 per sample) were homogenized in 0.05% v/v Tween-20. Samples were incubated for 5 min at 70°C, and supernatants assayed using Triglyceride Infinity Reagent (TR22421, Thermo Fisher Scientific) in a 96-well plate, measuring the absorbance at 540 nm in a plate reader (FLUOstar Omega, BMG Labtech) against a glycerol standard curve. Protein content of homogenates was determined using the BCA assay (Pierce #23225) for normalization.

Lipid Staining

For fat body staining, live flies were anesthetized on ice and dissected in ice-cold PBS. The ventral cuticle was cut away from the abdomen using microscissors, and the internal organs and genitalia were removed, leaving the intact abdominal fat body attached to the dorsal cuticle. Samples were fixed in 4% paraformaldehyde on ice for 30 min followed by 3x 10 min washes in PBS. Tissues were then incubated in Nile Red stain (10 μg/mL; Invitrogen N1142), followed by 3x 10 min washes in PBS, and finally mounted onto a slide in VectaShield with DAPI. Images were obtained by confocal microscopy (Leica SP5 II) at excitation/emission 552/636 nm. Brightness was adjusted in Adobe Photoshop to allow visualization of lipid droplet size.

Hemolymph Analysis

To extract hemolymph, live cohorts of 12 flies (n > 6 replicates per condition) were decapitated under mild CO_2 anesthesia using a scalpel blade, and the bodies were placed upside down into pipette tips within a 1.5 mL tube, and gently centrifuged for 15 min at 1,500 x g, 4°C to drain the hemolymph. The volume of extracted hemolymph was recorded using a pipettor. Glucose content was assayed using Glucose Infinity Reagent (TR15421, Thermo Fisher Scientific) in a 96-well plate, measuring the absorbance at 340 nm in a plate reader (FLUOstar Omega, BMG Labtech). To measure trehalose content, 10 mU of trehalase enzyme (#E-TREH, Megazyme) was added to the sample wells to cleave the disaccharide trehalose into two glucose molecules and incubated at 37°C for a minimum of 2 h. Samples were diluted 10-fold into fresh Glucose Infinity Reagent, and the assay repeated to obtain the amount of glucose released from trehalose. For pH analysis, hemolymph was extracted as described above, except that flies were anesthetized and decapitated on ice rather than CO_2. Hemolymph pH was measured using the pH-sensitive dye pyranine (Invitrogen H348) adapted from a published protocol (Ghosh and O'Connor, 2014). 1 μL of pyranine dye (2.4 mM stock in H_2O) was mixed with 1 μL of hemolymph sample, and the absorbance ratio 485/424 nm (corresponding to the protonated and deprotonated peaks of pyranine) was measured using a NanoDrop One (Thermo Fisher). A standard curve was prepared using 1 μL of 50 mM Tris-HCl ranging between pH 6.6 and 7.8, plotted as a second degree polynomial equation.

Fat Body Insulin and Glucose Assays

To assess insulin sensitivity, fat bodies (abdominal carcasses with the ovaries and digestive tract removed, n = 5 per sample) were dissected from live females anesthetized on ice and pooled into 1 mL Schneider's medium (GIBCO 21720024). Samples were incubated for 15 min at 25°C, then treated ± 5 μM insulin (Sigma I9278) for a further 15 min at 25°C. The supernatant was removed and the fat bodies were homogenized into Laemmli buffer (BioRad #1610737) with 5% v/v β-mercaptoethanol. Samples were analyzed by western blotting, probing for phospho-AKT-Ser505 (1:1,000; Cell Signaling #4054) with total AKT (1:1,000; Cell Signaling #9272) and actin (1:1,000; Abcam #ab8224) as controls. Bands were analyzed by densitometry using FIJI software (ImageJ; Schindelin et al., 2012). To measure glucose uptake, dissected adult fat bodies (n = 5 per sample) were incubated in 100 μL Schneider's medium with 200 μM 2-NBDG (2-(*N*-(7-nitrobenz-2-oxa-1,3-diazol-4-yl)amino)-2-deoxyglucose; Invitrogen N13195) ± 5 μM insulin for 15 min at 25°C, protected from light. Samples were washed twice, then homogenized in 120 μL PBS using a pellet pestle and motor. 100 μL of supernatant was loaded in a 96-well plate, and the fluorescence (excitation/emission 485/520 nm) measured in a plate reader (FLUOstar Omega, BMG Labtech) against a 2-NBDG standard curve.

Protein Glycation and Carbonylation Assays

Levels of glycation in whole frozen flies (n = 10 per samples) were assessed by western blotting using an anti-AGE (advanced glycation end-product) antibody (1:2,000; Millipore AB9890), normalized against total protein assessed using the stain-free labeling system (Bio-Rad). Carbonylation was measured using the OxyBlot Protein Detection Kit (Millipore S7150). Whole frozen flies (n = 5 per sample) were homogenized in 100 μL of CelLytic M Cell Lysis Reagent (Sigma C2978) supplemented with 50 mM DTT and protease inhibitor cocktail (complete mini EDTA-free, Roche 11 836 170 001). Protein samples (20 μg, assayed by the Bradford method) were

subjected to OxyBlot derivatization according to the manufacturer's instructions, followed by western blotting. Lanes were analyzed by densitometry using Image Lab software (Bio-Rad).

Gut Function Assays

Intestinal function was determined by analyzing fly excreta (Cognigni et al., 2011). Flies (n = 5 females per plate) pre-treated for 7 days on 5%S or 20%S ± H_2O were transferred without anesthesia to a 6 cm diameter Petri dish containing a $^1/_4$ wedge of the same food type supplemented with 2.5% w/v blue food dye (– H_2O), or a $^1/_8$ wedge of dyed food alongside a $^1/_8$ wedge of undyed 1.5% w/v agar, separated by a strip of cellophane to prevent diffusion (+ H_2O) (see Figure S4D). Flies were incubated in the inverted dishes for 24 h, then the plate lids were scanned (Epson V700 Photo, 1200 dpi), and analyzed by TURD software (version 0.8; Wayland et al., 2014) with the following parameters: block size: 25, offset: 10, brush shape: circle, brush size 3, min size: 35 (max 3,500), circularity threshold: 0.6. For pH analysis of excreta, 5%S and 20%S media were prepared without propionic acid or nipagin, then supplemented with 0.5% w/v bromophenol blue (Sigma B5525) or bromocresol purple (Sigma B5880), and the final pH adjusted to 5.5. Plates were prepared as above and incubated for 48 h at 25°C. Males were used for these experiments to avoid interference from crawling larvae. The TURD software parameters were as above, except the block size was set to 99. Intestinal barrier integrity was assayed by the Smurf assay (Rera et al., 2012). Flies were aged for 28 days on 5%S or 20%S ± H_2O, then transferred onto media supplemented with 2.5% w/v blue food dye. After 24 h, flies were scored for a Smurf phenotype (see Figure 4G inset).

Tubule and Rectal Ampulla Imaging

For imaging of the rectal ampulla and tubules, live flies were anesthetized on ice, dissected in ice-cold PBS onto poly-L-lysine coated slides, and imaged on a Nikon Eclipse 50i microscope with a DXM1200C digital camera. Images were stitched together in Adobe Photoshop CC, using the automated Photomerge function. To quantify the tubule stone phenotype, each of the 4 tubule arms per fly was scored according to the scale in Figure S5A, and averaged to give a mean score per fly (ranging between 0-4).

Uric Acid Assay

Uric acid levels were quantified spectrophotometrically using the QuantiChrom Uric Acid Assay Kit (DIUA-250, BioAssay Systems) according to the manufacturer's instructions. Whole frozen bodies (n = 5 flies per sample) were homogenized in 100 μL of 0.05% v/v Tween-20 using a pellet pestle and motor, while tubules dissected from live flies (n = 6 tubule pairs per sample) were homogenized in 12 μL. Samples and uric acid standards (5 μL) were loaded in a 96-well plate with 200 μL of assay buffer, incubated for 30 min at 30°C, and the absorbance at 590 nm measured in a plate reader (FLUOstar Omega, BMG Labtech). For hemolymph analysis, samples were added directly to the assay buffer.

Tubule Secretion Assay

Tubule secretion rates were measured as described (Dow et al., 1994; Schellinger and Rodan, 2015). Briefly, live flies were anesthetized on ice and the tubules dissected in ice-cold 1:1 *Drosophila* saline:Schneider's medium, then carefully cut at the ureter. One arm of the tubule pair was wrapped around a metal pin secured in a silicone plate, while the other arm was immersed in a bathing drop of 1:1 *Drosophila* saline:Schneider's medium colored with blue food dye for visualization, leaving the ureter exposed under a layer of heavy mineral oil (Sigma 330760). See Figure S5E for a scheme of the experimental setup. Drops secreted from the ureter were removed at 10 min intervals over the course of 1 h using a fine glass rod, and their diameter measured under a microscope. The volume of each drop was calculated ($= 4/3\ \pi r^3$) to give a secretion rate in nL/min.

Metabolomics Analysis of Rectal Ampulla Stones

Dissected rectal ampulla stones (a combined score of 25 per sample according to the scale in Figure S6B) were dissolved in 100 μL of extraction solvent (20:80 v/v water/methanol) and spiked with 150 ng/mL $^{13}C_5$-hypoxanthine (CLM-8042; Cambridge Isotope Laboratories) as internal standard (IS). Samples (n = 4 per condition) were vortexed (30 s), centrifuged (13,000 x g, 2 min), sonicated using an ultrasonic water bath (15 min) and finally centrifuged again (13,000 x g, 10 min). The supernatant was filtered using spin-filter tubes (PTFE 0.22 μm; Thermo Scientific). Pooled quality controls (QCs) were created for each batch by pooling equal aliquots of each study sample in the batch, in order to assess technical replicate reproducibility.

Liquid chromatography high-resolution mass spectrometry analysis (LC-HRMS) was carried out on a Vanquish Flex Binary UHPLC system (Thermo Scientific) coupled to a benchtop hybrid quadrupole-Orbitrap Q Exactive mass spectrometer (Thermo Scientific). Separation was achieved using an Accucore 150 Amide HILIC column (Thermo Scientific, 150 × 2.1 mm, 2.6 μm) equipped with a guard column (Thermo Scientific, 30 × 2.1 mm, 2.6 μm), both held at a temperature of 40°C and a flow rate of 0.2 mL/min. Mobile phases were 10 mM ammonium acetate (pH 4.60) in 90% (v/v) acetonitrile (solvent A) and 10 mM ammonium acetate (pH 4.60) in 10% (v/v) acetonitrile (solvent B). The gradient elution was performed with a 0%–20% solvent B gradient over 10 min, ramping to 47% B at 12 min, held at this condition for 6 min, and returning to 0% B at 20 min. The column was equilibrated for 10 min, yielding a total run time of 30 min. Ionization was performed in the negative mode using a heated electrospray ionization source, with the following parameters: spray voltage 3.0 KV, heater temperature 330°C, capillary temperature 320°C, S-lens RF level 50, sheath and auxiliary gas flow rate, 35 and 10 units, respectively. The mass accuracy was calibrated prior to sample analysis. Mass spectrometric data were acquired in profile mode using the full scan setting (m/z 70-1050). Automatic gain control (AGC) was set to 1e6 and maximum MS^1 injection time at 250 ms. Metabolomics data acquisition and processing was performed with Xcalibur software (version 4.1). Relative

quantification is reported based on the peak area ratio of the analyte and the IS. For selected compounds (hypoxanthine, xanthine and allopurinol), calibration curves (12.5-250 ng/mL; $R^2 > 0.9974$) were performed to assess their absolute amounts. Details of the compounds quantified by HILIC UPLC-HRMS are listed in Table S4.

Human Blood Analysis and Metabolomics

Blood samples were obtained after an overnight fast and processed for fasting blood glucose and triglyceride levels, as previously detailed (Koch et al., 2014). Untargeted metabolomics profiling by LC-MS/MS analysis was performed at the Helmholtz Zentrum München, Germany, as described (Koch et al., 2017). Briefly, protein was precipitated from 100 μL of blood serum and the metabolites were extracted with 475 μL methanol. Duplicate samples were processed in positive (in 50 μL 0.1% formic acid) and negative (in 50 μL 6.5 mM ammonium bicarbonate, pH 8.0) electrospray ionization mode. Reconstitution solvents were mixed with internal standards as retention reference markers. Metabolite identification was performed by comparison of the recorded LC-MS/MS spectra against the Metabolon library, based on retention index, precursor mass and MS/MS fragmentation patterns (Koch et al., 2017). Detected purines were adenosine, guanosine, xanthosine, inosine, xanthine, hypoxanthine, uric acid (see Figure S7A). Adenosine was excluded from further analysis due to equal values across all study participants in our cohort subset.

Food Questionnaire and Molecular Diet Composition

Dietary information for the past 12 months was obtained from a food frequency questionnaire, previously used in the EPIC cohorts, where food items were divided into 16 main groups and further sub-groups (Brandstetter et al., 1999). For our statistical analysis, we considered vegetables (including potatoes and pulses), milk and dairy products, cereals and products thereof, meat, fish, eggs, and sweets as separate main groups. Fat, sauces, soups, and unclassified foods were combined into a mixed group termed 'other'. Alcoholic and non-alcoholic beverages were also included in this 'other' group, except the sub-groups 'juices' and 'soft drinks', which were considered separately in our analysis.

The metabolic composition of the diet was imputed from the food questionnaire, providing detailed amounts of dietary compounds consumed by each individual participant. Molecular concentrations were estimated based on molecular weights and amounts of each of the 141 consumable food items for each participant (Pryor et al., 2019). Following the *Drosophila* experiments, we focused on dietary sugars and nucleotides of the purine metabolism for statistical testing of metabolites. The dietary purines tested were uric acid, adenosine, deoxyadenosine, guanosine, deoxyguanosine, xanthine and hypoxanthine. The dietary sugars tested were lactose, maltose, sucrose, fructose, galactose, glucose, maltotriose and ribose. Both food groups and dietary metabolites employed in statistical tests were normalized for individual caloric intake and multiplied by the median consumed calories of our study population (8,948.1 kJ).

Analysis of Variance

Permutational multivariate analysis of variance (PERMANOVA; R-package 'vegan' version 2.5-6) testing was performed with the Euclidean inter-sample distances as dependent variable based on eGFR or metabolite serum levels. Independent variables were food groups or dietary metabolites together with clinical parameters. The estimation of explained variance for each term was performed via marginal testing. Probability values were generated by comparing F-distributions of the original data to that of 9999 random sample permutations.

Linear Modeling

Linear regression models were calculated for each measured serum purine as a dependent variable separately (R-package 'stats' R-version 3.6.1). Independent variables were dietary glucose, BMI, eGFR, age and fasting plasma glucose concentrations. Probability values for the terms of interest, BMI, eGFR and dietary glucose, were corrected for multiple testing (Benjamini and Hochberg, 1995) across all six linear models (one for each circulating purine). The slope of the explanatory variables was evaluated, and considered as positively (> 0) or negatively (< 0) correlated. Linear regression analysis for serum uric acid concentrations explained by eGFR (Figure S7B) or BMI (Figure S7K) was calculated without any further independent terms or multiple testing correction.

QUANTIFICATION AND STATISTICAL ANALYSIS

Fly statistical analysis

Survival curves were analyzed by log-rank test. The majority of other experiments were analyzed by one-way ANOVA with Tukey's multiple comparison. Tubule and ampulla scoring was analyzed by Kruskal-Wallis test with Dunn's multiple comparison. Data were plotted and analyzed using GraphPad Prism 8 software. Significance of probability values: n/s, $p > 0.05$; *$p < 0.05$; **$p < 0.01$; ***$p < 0.001$. Sample size is not limiting for *Drosophila* studies, and typically n $\sim$ 150 flies were pre-determined and used per condition for survival assays (see Tables S2 and S3 for exact n numbers). For other assays, sample size was determined based on the significance obtained from previous studies with similar experimental setups, or empirically based on the variability of each scored phenotype. Comparable sample sizes were used in each experiment. Experiments were not blinded, except for the feeding assay based on observation of proboscis extension. Experiments were excluded if the positive control failed to work. Control and experimental flies were bred under identical conditions, and were randomized with regard to dietary and/or drug treatment.

Human Statistical Analysis

Data were analyzed in R statistical software using the packages ‘vegan’ (version 2.5-6) and ‘stats’ (R-version 3.6.1), and visualized with the package ‘RColorBrewer’. Significance of probability values or false discovery rates (FDR): n/s, $p > 0.1$; •$p < 0.1$; *$p < 0.05$; **$p < 0.01$; ***$p < 0.001$.

DATA AND CODE AVAILABILITY

Fly data generated during this study are included in this published article and its supplemental information files. Human cohort data is available upon application from the PopGen biobank (https://www.uksh.de/p2n/Information+for+Researchers.html).

Cell Metabolism, Volume *31*

Supplemental Information

Sugar-Induced Obesity and Insulin Resistance Are Uncoupled from Shortened Survival in *Drosophila*

Esther van Dam, Lucie A.G. van Leeuwen, Eliano dos Santos, Joel James, Lena Best, Claudia Lennicke, Alec J. Vincent, Georgios Marinos, Andrea Foley, Marcela Buricova, Joao B. Mokochinski, Holger B. Kramer, Wolfgang Lieb, Matthias Laudes, Andre Franke, Christoph Kaleta, and Helena M. Cochemé

Supplemental Figure 1

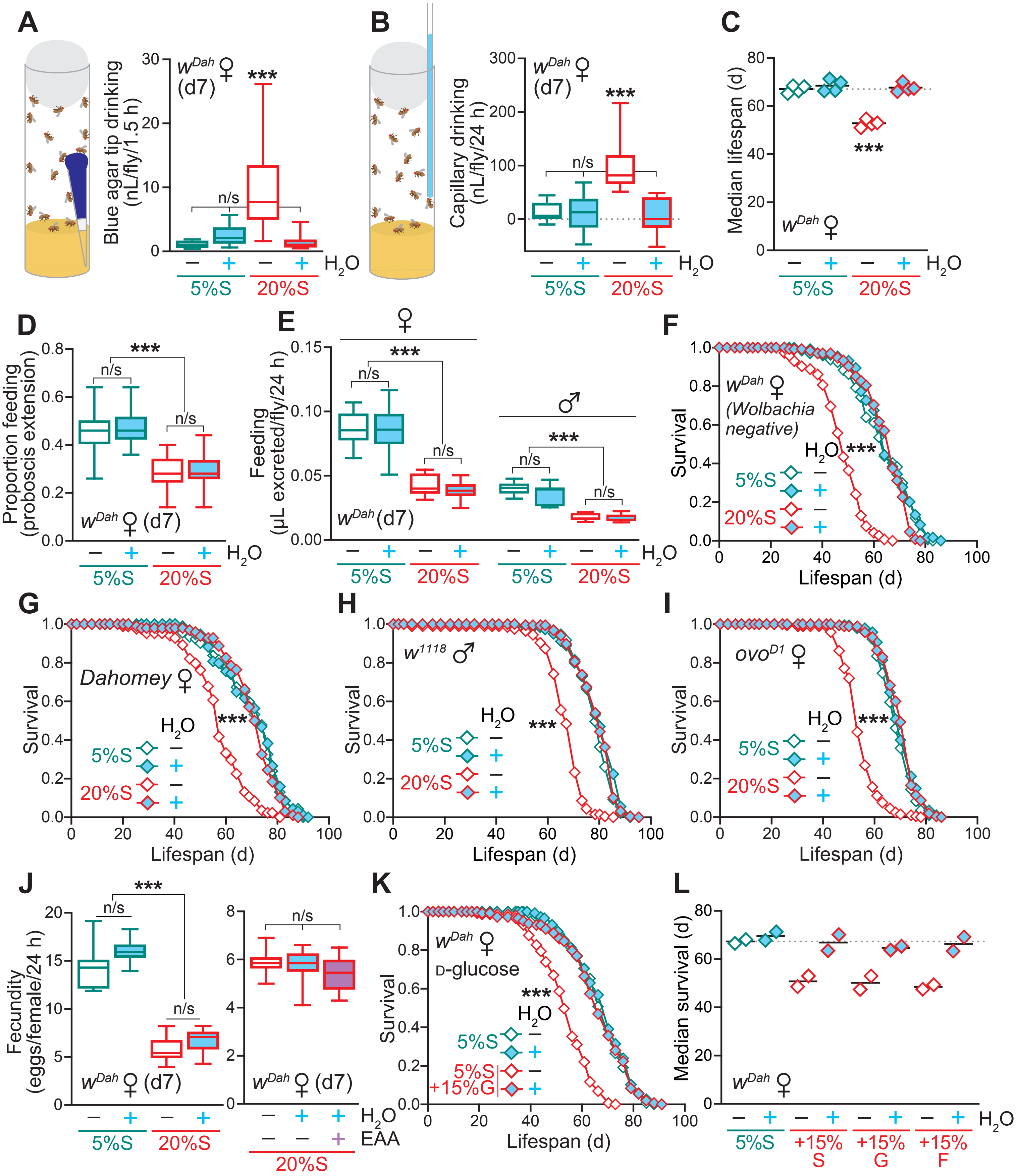

Figure S1. A High-Sucrose Diet that Induces Thirst, Dehydration and Shortens Lifespan in Adult *Drosophila* Is Rescued by Water Supplementation (Related to Figure 1)

(A and B) Drinking assays to quantify the thirst of WT (w^{Dah}) flies in response to a high-sugar diet. Females were pre-treated for 7 days on 5%S or 20%S ± H_2O. Data (n = 10 replicates per condition, each with 15 flies) are presented as box-and-whisker plots (min–max error bars), analyzed by one-way ANOVA with Tukey correction (n/s, $p > 0.05$; ***$p < 0.001$). (A) Blue dye drinking assay, with flies provided agar colored by blue dye and incubated for 1.5 h. The amount of dye ingested was then analyzed spectrophotometrically from fly homogenates to estimate the volume of water consumed from the agar tips. (B) Capillary drinking assay of flies incubated for 24 h. The drinking volume was corrected for an evaporation control, consisting of a capillary inserted into an empty vial (n = 8).

(C) Summary of median survival for n = 4 independent lifespan experiments of WT females on 5%S and 20%S ± H_2O. Data were analyzed by one-way ANOVA with Tukey correction (n/s, $p > 0.05$; ***$p < 0.001$).

(D and E) Feeding assays of WT flies pre-treated for 7 days on 5%S or 20%S ± H_2O. (D) Behavioral proboscis extension assay (n = 10 vials per condition, each with 5 females), with the proportion of flies feeding in each vial observed every ~2 min over a 90 min time course. Data are the average of n = 46 time points over the full experiment, presented as box-and-whisker plots (min–max error bars) and analyzed by one-way ANOVA with Tukey correction (n/s, $p > 0.05$; ***$p < 0.001$). (E) Quantification of fly feeding based on the excretion of food containing a blue tracer dye. WT females (n = 10 vials per condition, each with 15 flies) and males (n = 10 vials per condition, each with 20 flies) were pre-treated for 7 days on 5%S or 20%S ± H_2O, then exposed to the same food supplemented with 1% blue dye ± H_2O for 24 h at 25ºC. Excreta from the vials were then solubilised, and the amount of blue dye was assayed spectrophotometrically. Data are presented as box-and-whisker plots (min–max error bars), analyzed by one-way ANOVA with Tukey correction (n/s, $p >0.05$; ***$p < 0.001$).

(F) Lifespan of w^{Dah} females, negative for the endosymbiont *Wolbachia*, on 5%S and 20%S ± H_2O (n ~ 150 flies per condition).

(G) Lifespan of *Dahomey* females on 5%S and 20%S ± H_2O (n ~ 90–105 flies per condition).

(H) Lifespan of w^{1118} males on 5%S and 20%S ± H_2O (n ~ 200 flies per condition).

(I) Lifespan of sterile ovo^{D1} mutant females on 5%S and 20%S ± H_2O (n ~ 200 flies per condition).

(J) Fecundity (eggs laid/female/24 h) of WT females pre-treated for 7 days on 5%S or 20%S ± H_2O. Supplementation of the agar tips with a cocktail of essential amino acids (EAA) did not increase fecundity of females pre-treated on a 20%S diet for 7 days (n = 10 vials per condition, each with 15 flies). Data are presented as box-and-whisker plots (min–max error bars), analyzed by one-way ANOVA with Tukey correction (n/s, $p > 0.05$; ***$p < 0.001$).

(K) Lifespan of WT females ± H_2O on a control diet (5%S) supplemented with 15% D-glucose (n ~ 150 flies per condition).

(L) Summary of median survival ± H_2O for n = 2 independent lifespan experiments on excess 15% D-glucose or D-fructose, compared to the 20%S high-sucrose diet.

Statistical analysis of survival curves (F,G,H,I,K) was performed by log-rank test (n/s, $p > 0.05$; ***$p < 0.001$). See Table S3 for exact n numbers and p values.

Supplemental Figure 2

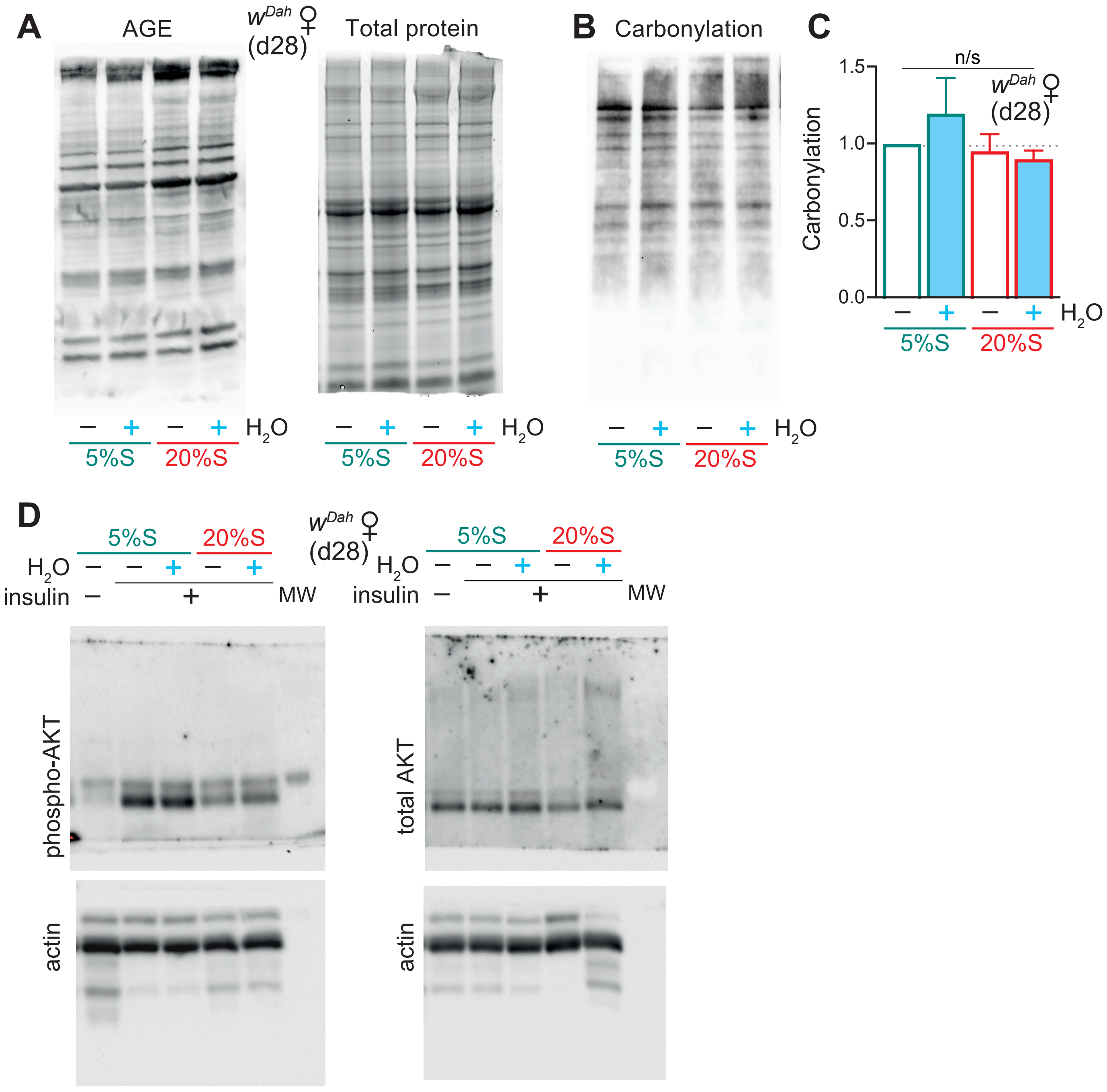

Figure S2. Metabolic Effects of a High-Sucrose Diet and Water Supplementation in WT Adults (Related to Figure 2)

(A) Glycation damage in whole body WT (*w*^{Dah}) females fed for 28 days on 5%S or 20%S ± H_2O. Full anti-AGE western blot and stain-free gel image of total protein relating to the quantification in Figure 2C.

(B and C) Protein carbonylation levels in whole body WT females fed for 28 days on 5%S or 20%S ± H_2O, assessed by oxyblot (B), and quantification by densitometry (C). Data are means + SEM of n = 3 experiments (with n = 5 flies per sample), analyzed by one-way ANOVA (n/s, $p > 0.05$).

(D) Insulin resistance of WT females fed for 28 days on 5%S or 20%S ± H_2O, assessed by AKT phosphorylation. Full western blots relating to Figures 2E,F. Dissected fat bodies (n = 5 per sample) were incubated ± insulin for 15 min, then homogenized in Laemmli buffer. Samples were run in parallel on two SDS-PAGE gels. The blots were cut horizontally, and the upper portion was probed against phospho-AKT and total AKT respectively (~65 kDa), while both lower portions were probed against actin as a loading control (~42 kDa).

Supplemental Figure 3

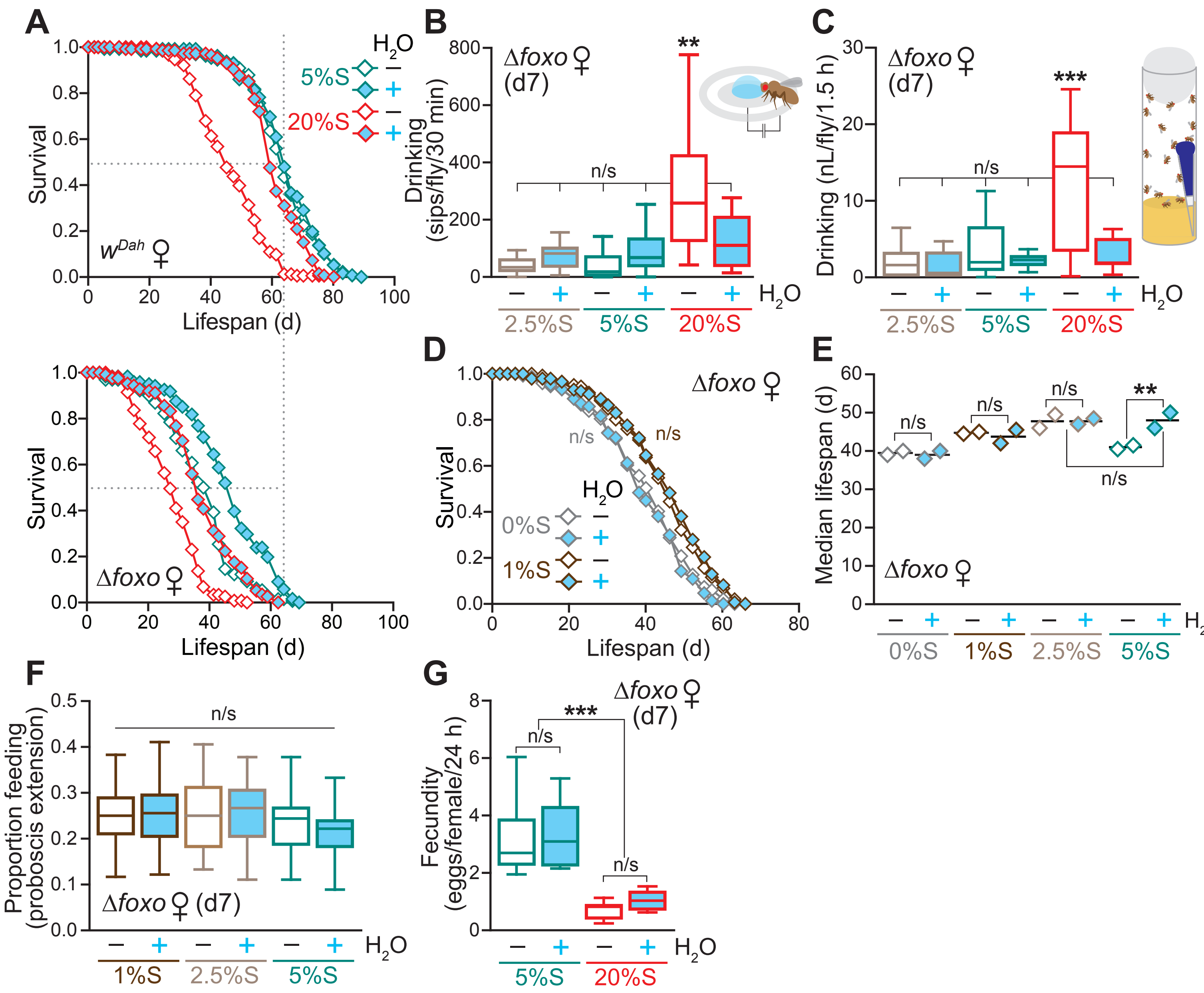

Figure S3. Δ*foxo* Mutants are Hypersensitive to Dietary Sugar (Related to Figure 3)

(A) Lifespan of w^{Dah} and Δ*foxo* females on 5%S and 20%S ± H_2O from a simultaneous experiment (n ~ 135–150 flies per condition). The grey dotted lines indicate median survival.

(B and C) Drinking assays of Δ*foxo* females pre-treated for 7 days on 2.5%S, 5%S or 20%S ± H_2O: (B) FlyPAD (n = 14–19 individual flies per condition), and (C) blue-dyed agar tips (n = 10 vials per condition, each with n = 15 flies). Box-and-whisker plots (min–max error bars), analyzed by one-way ANOVA with Tukey correction (n/s, $p > 0.05$; **$p < 0.01$; ***$p < 0.001$).

(D) Lifespan of Δ*foxo* females on 0%S and 1%S ± H_2O (n ~ 150 flies per condition). Statistical analysis was performed by log-rank test (n/s, $p > 0.05$). See Table S3 for exact n numbers and p values.

(E) Summary of median survival for 2 independent Δ*foxo* lifespan experiments on 0%S, 1%S, 2.5%S and 5%S ± H_2O. Data were analyzed by one-way ANOVA (n/s, $p > 0.05$; **$p < 0.01$).

(F) Quantification of fly feeding based on behavioral proboscis extension. Δ*foxo* females (n = 10 vials per condition, each with 5 flies) were pre-treated for 7 days on 1%S, 2.5%S or 5%S ± H_2O. The proportion of flies feeding in each vial was observed every ~3 min over a 90 min time course. Data are the average of n = 31 time points over the full experiment, presented as box-and-whisker plots (min–max error bars) and analyzed by one-way ANOVA with Tukey correction (n/s, $p > 0.05$).

(G) Fecundity of Δ*foxo* females pre-treated for 7 days on 5%S or 20%S ± H_2O (n = 10 vials per condition, each with 15 flies). Box-and-whisker plots (min–max error bars), analyzed by one-way ANOVA with Tukey correction (n/s, $p > 0.05$; ***$p < 0.001$).

Supplemental Figure 4

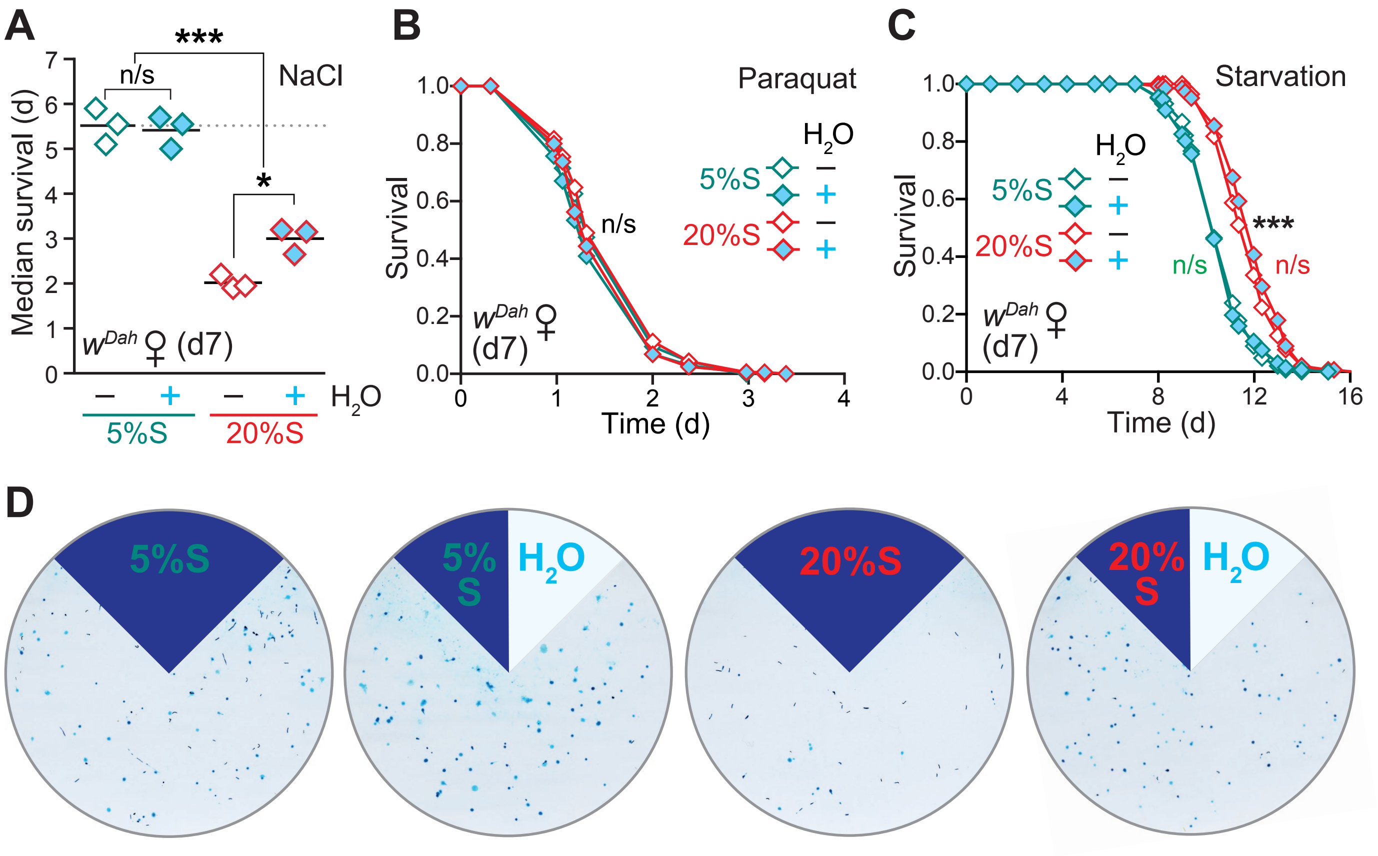

Figure S4. Effects of a High-Sugar Diet on Stress Responses and Gut Physiology (Related to Figure 4)

(A) Summary of median survival for 3 independent stress assays on 500 mM NaCl (see Figure 4B). Data were analyzed by one-way ANOVA with Tukey correction (n/s, $p > 0.05$; *$p < 0.05$; ***$p < 0.001$).

(B and C) Stress response of WT (w^{Dah}) females pre-treated for 7 days on 5%S or 20%S ± H_2O, then exposed to: oxidative stress consisting of 20 mM paraquat in 5%S food (B, n ~ 160 per condition), and starvation stress consisting of 1.5% agar (C, n ~ 150 flies per condition).

(D) Experimental setup and typical scans for the analysis of fly excreta. Food was supplemented with 2.5% w/v blue dye, while the agar for the water supplementation was undyed.

Statistical analysis of survival curves (B,C) was performed by log-rank test (n/s, $p > 0.05$; ***$p < 0.001$). See Table S3 for exact n numbers and p values.

Supplemental Figure 5

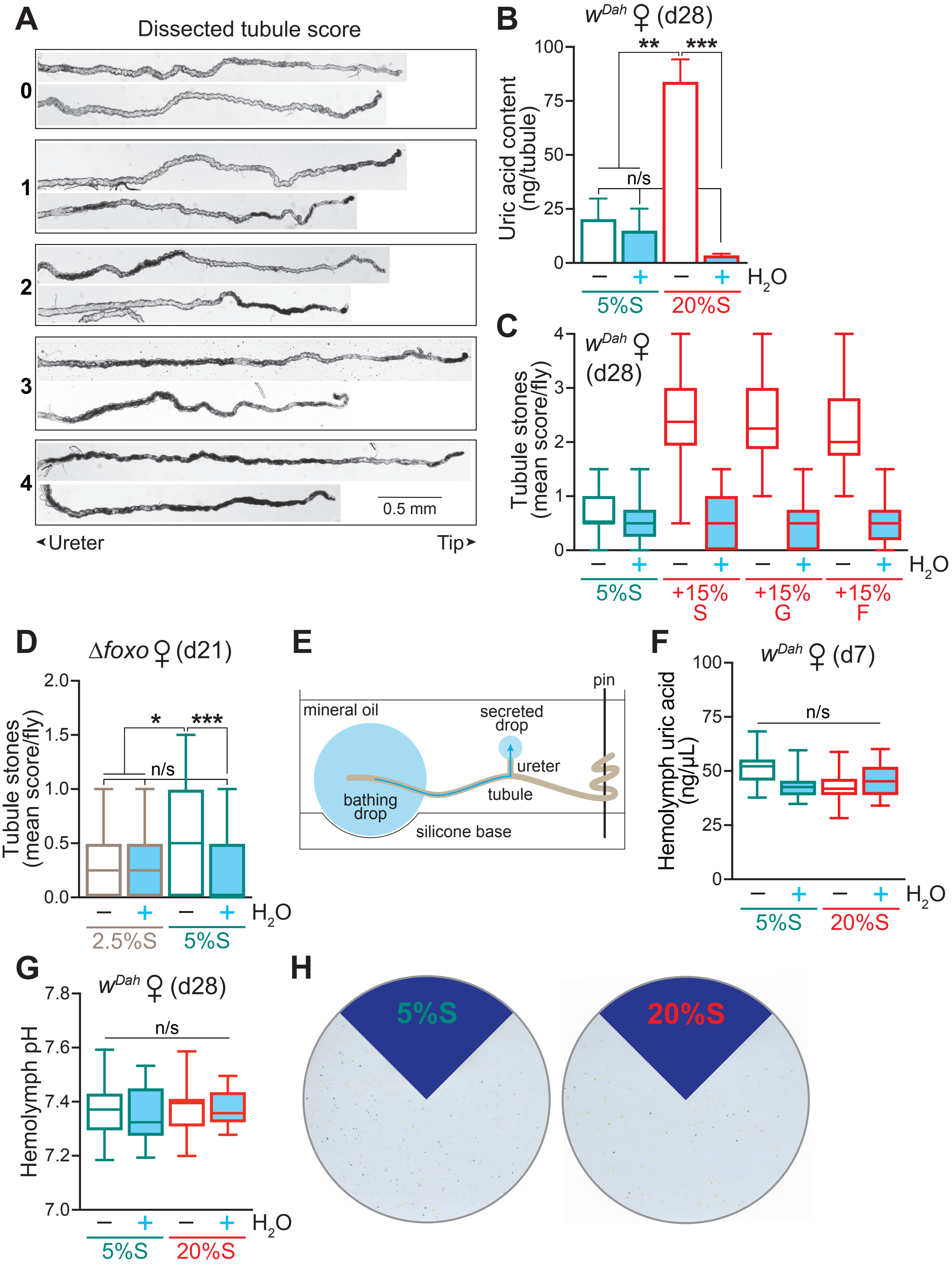

Figure S5. A High-Sugar Diet Induces Uric Acid Deposition and Tubule Dysfunction (Related to Figure 5)

(A) Scoring of the tubule stone phenotype based on light microscopy imaging of dissected tubules, arranged from the ureter (left) to the distal tip (right). Scale bar = 500 µm. Scoring: 0 = clear (~0%), 1 = mild (< 25%), 2 = moderate (25–50%), 3 = strong (50–75%), 4 = severe (> 75%).

(B) Uric acid content of dissected tubules from WT (w^{Dah}) females fed for 28 days on 5%S or 20%S ± H_2O. Data are means + SEM of n = 3 replicates (each with n = 6 pairs of tubules per sample), analyzed by one-way ANOVA with Tukey correction (n/s, p > 0.05; **p < 0.01; ***p < 0.001).

(C) Tubule phenotype scoring according to the scale in (A) of WT females maintained for 28 days ± H_2O on excess 15% D-glucose or D-fructose, compared to the 5%S and 20%S sucrose diets (n = 100 flies per condition). Data are box-and-whisker plots (min–max error bars), analyzed by Kruskal-Wallis test with Dunn correction (n/s, p >0.05; ***p < 0.001).

(D) Tubule phenotype scoring according to the scale in (A) of Δ*foxo* females maintained for 21 days on 2.5%S and 5%S ± H_2O. Box-and-whisker plots with min–max error bars (n = 57–70 flies per condition), analyzed by Kruskal-Wallis test with Dunn correction (n/s, p > 0.05; *p < 0.05; ***p < 0.001).

(E) Scheme of the tubule secretion assay. One arm of the tubule is bathed in a drop of saline with blue food dye, while the other is secured with a pin. Drops secreted from the ureter are measured over time.

(F) Uric acid levels in the hemolymph of flies fed for 7 days on 5%S or 20%S ± H_2O (n = 12 replicates per condition, each with 12 flies per sample). Box-and-whisker plots (min–max error bars), analyzed by one-way ANOVA with Tukey correction (n/s, p > 0.05).

(G) Hemolymph pH of WT females treated for 28 days on 5%S or 20%S ± H_2O (n = 9–17 samples, with 12 flies per sample). Box-and-whisker plots with min–max error bars, analyzed by one-way ANOVA with Tukey correction (n/s, p > 0.05).

(H) Typical scanned plates for the analysis of excreta pH using media supplemented with bromocresol purple (see Figure 5L).

Supplemental Figure 6

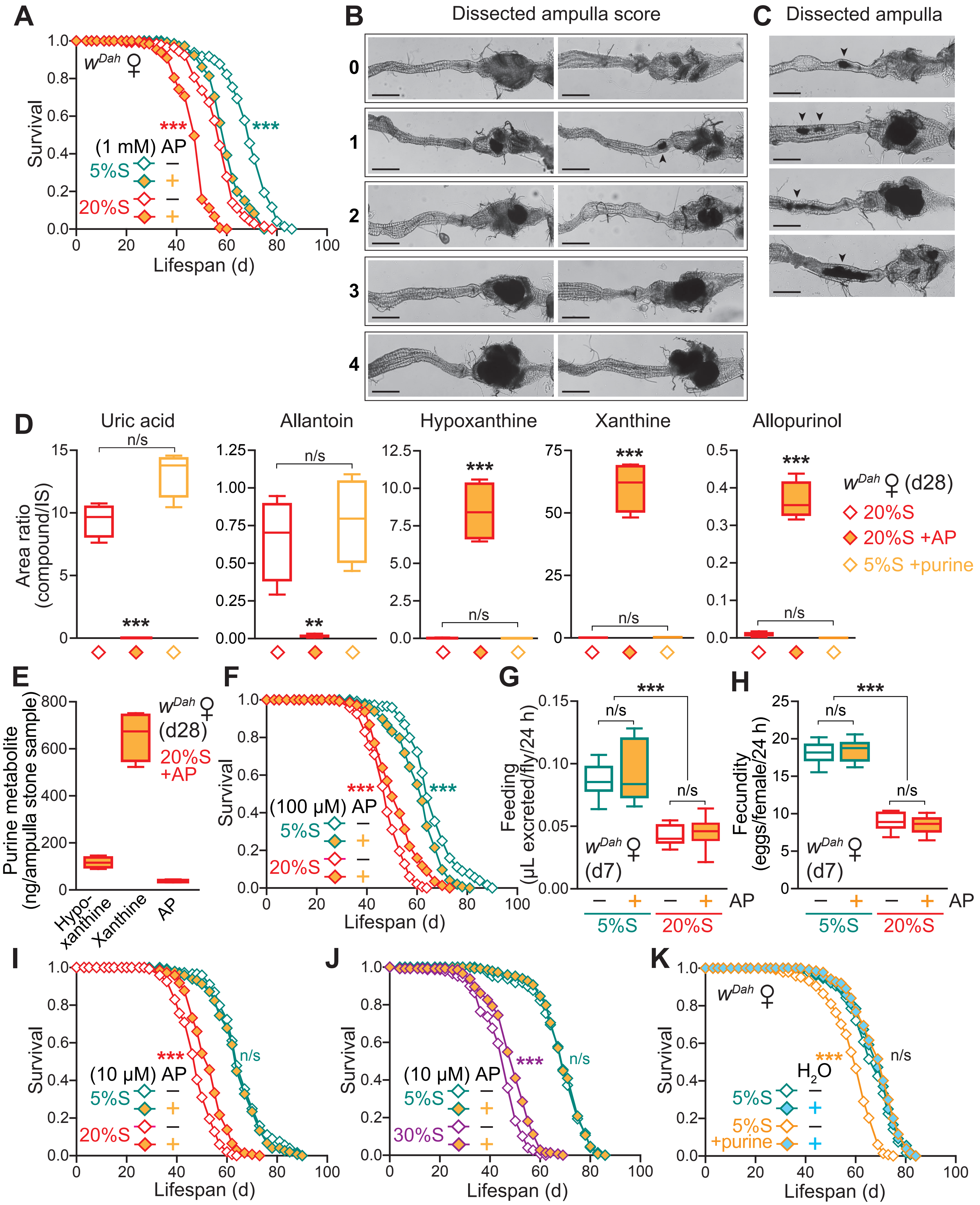

Figure S6. Pharmacological Treatments and Dietary Interventions Targeting Purine Metabolism Impact on Lifespan (Related to Figure 6)

(A) Chronic high allopurinol (1 mM AP) shortens the lifespan of WT (w^{Dah}) females on both the control (5%S ± AP) and high-sugar (20%S ± AP) diets (n ~ 135–150 flies per condition).

(B) Scoring of the rectal ampulla stone phenotype based on light microscopy imaging of dissected hindguts. Scale bar = 100 µm. Scoring: 0 = clear, 1 = mild, 2 = moderate, 3 = strong, 4 = severe.

(C) Light microscopy images showing concretions in the anterior hindgut (arrowheads), apparent upon allopurinol treatment.

(D) Metabolomics analysis of dissected rectal ampulla stones from WT females fed high-sugar (20%S), allopurinol-treated (20%S + AP, 1 mM), or high-purine (5%S + purine, 10 mM) diets for 28 days. n = 4 samples per condition, each with 25 units of stones as determined by the scoring scale in (B). Peak area ratios for the metabolite relative to the internal standard (IS) are presented as box-and-whisker plots (min–max error bars), analyzed by one-way ANOVA with Tukey correction (n/s, $p > 0.05$; ***$p < 0.001$).

(E) Quantification of hypoxanthine, xanthine and allopurinol levels in dissected rectal ampulla stones formed on the 20%S + AP condition (n = 4 per condition). Peak area data from (D) were converted to ng/sample based on calibration curves for each metabolite, and presented as box-and-whisker plots (min–max error bars).

(F) Independent biological repeat of the lifespan on 5%S and 20%S ± 100 µM AP in Figure 6C (n ~ 150 flies per condition).

(G and H) Dietary AP (100 µM) pre-treatment of WT females for 7 days does not affect feeding assessed by the blue dye excretion method (G) or fecundity (H) on each respective diet. Data (n = 10 replicates per condition, each with 15 flies per vial) are presented as box-and-whisker plots (min–max error bars), analyzed by one-way ANOVA with Tukey correction (n/s, $p > 0.05$; ***$p < 0.001$).

(I and J) Supplementation with 10 µM AP extends the lifespan of WT females on the 20%S (I) or 30%S (J) high-sugar diets, without affecting survival on control food 5%S ± AP (n ~ 150 flies per condition).

(K) Independent biological repeat of the lifespan on high-purine (10 mM) ± H_2O in Figure 6F (n ~ 135–150 flies per condition).

Statistical analysis of survival curves (A,F,I,J,K) was performed by log-rank test (n/s, $p > 0.05$; ***$p < 0.001$). See Table S3 for exact n numbers and p values.

Supplemental Figure 7

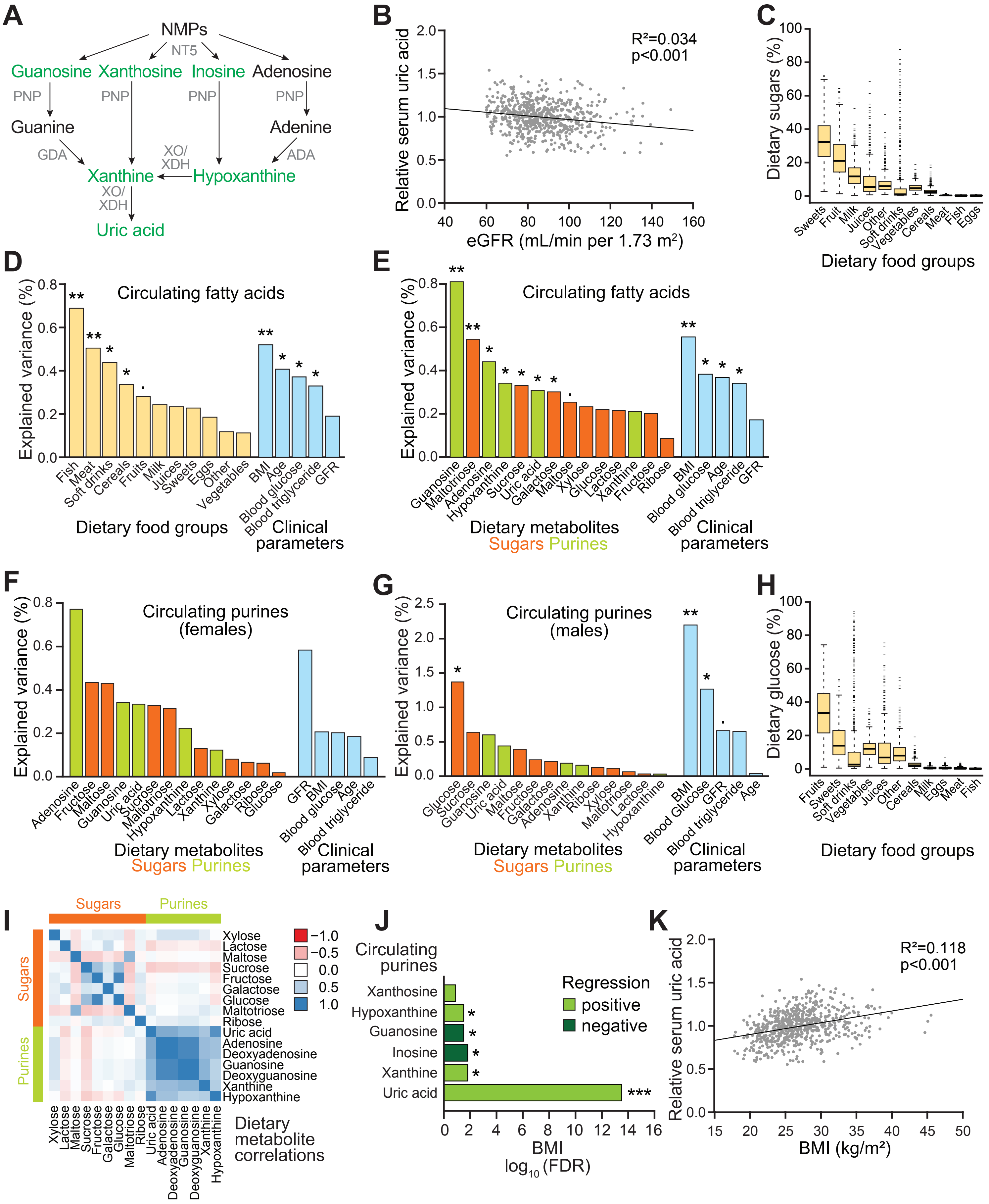

Figure S7. Human Metabolomics Analysis Links Dietary Sugar Intake with Renal Function and Circulating Purine Levels (Related to Figure 7)

(A) Scheme of the purine catabolism pathway, with metabolites measured by LC-MS from human blood serum color-coded in green. Nucleotide monophosphates (NMPs; i.e., GMP, XMP, IMP and AMP) are converted by various 5'-nucleotidases (NT5) into their respective nucleosides. The next steps are catalyzed by purine nucleoside phosphorylase (PNP), and guanine or adenine deaminase (GDA and ADA, respectively). The conversion of hypoxanthine to xanthine and finally to uric acid is catalyzed by the enzyme xanthine oxidase (XO)/xanthine dehydrogenase (XDH).

(B) Scatter plot of estimated glomerular filtration rate (eGFR) against circulating uric acid levels for the full cohort of n = 650 participants. A linear model predicting serum uric acid levels from eGFR, without any further independent terms, was overlaid as a regression line.

(C) Relative contribution of each food group to an individual's total dietary sugar intake (summed mono- and disaccharides). Data for the full cohort of n = 650 participants are displayed as box-and-whisker plots of the interquartile range (IQR), with the line corresponding to median and whiskers to 1.5x IQR.

(D-G) PERMANOVA analysis of circulating metabolites against dietary food groups or dietary metabolites ($^{\bullet}p < 0.1$; $^{*}p < 0.05$; $^{**}p < 0.01$). Clinical parameters are separated for visual clarity. Explained variance of dietary food groups (D) or dietary metabolites (E), color-coded in orange for sugars and green for purines, on the concentrations of circulating fatty acids. Analysis of variance of dietary metabolites against circulating purines in only female (F) or male (G) participants.

(H) Relative contribution of each food group to an individual's total dietary glucose intake. Fruit consumption accounted for more than 33.4% of total glucose intake in half of the cohort. Soft drinks showed a broad distribution as apparent from the dots corresponding to outliers, with some participants receiving up to 93.8% of their total glucose intake from soft drinks, while others hardly consuming any soft drinks at all (minimum = 0.33% of total glucose intake). Data for the full cohort of n = 650 participants are displayed as box-and-whisker plots of the interquartile range (IQR), with the line corresponding to median and whiskers to 1.5x IQR.

(I) Correlations between dietary metabolites imputed from food items in the food questionnaire. Spearman's rank-order correlation coefficient (rho; range –1 to 1) plotted as a heatmap (blue = positive, red = negative correlation). Darker colours are indicative of stronger correlations. Amongst dietary sugars, the only clear pattern is a very strong positive correlation between the dietary intake of glucose and fructose (*rho* > 0.937).

(J) Linear model of BMI predicting serum levels of individual purines (same analysis as for eGFR, shown in Figure 7B). Logarithmic FDRs are plotted as bars and color-coded for positive (light green) or negative (dark green) regressions.

(K) Scatter plot of BMI against circulating uric acid levels for the full cohort of n = 650 participants. A linear model predicting serum uric acid levels from BMI, without any further independent terms, was overlaid as a regression line.

Table S1. *Drosophila* Diet Recipes (Related to STAR Methods)

Sucrose Diets (per L of Media)

		Low sucrose diets			Control diet	High sucrose diets		
**Ingredient**	**Source**	**0%S**	**1%S**	**2.5%S**	**5%S**	**20%S**	**30%S**	**40%S**
Sucrose (% w/v)	Tate & Lyle, Granulated sugar	0 g	10 g	25 g	50 g	200 g	300 g	400 g
Yeast (10% w/v)	MP Biomedicals, #903312	100 g						
Agar (1.5% w/v)	Sigma, A7002	15 g						
Nipagin	Sigma, H5501	30 mL of 10% w/v nipagin in 95% EtOH						
Propionic acid (0.3% v/v)	Sigma, P1386	3 mL						
H_2O		up to 1 L						

Other High Sugar Diets (per L of Media)

**Ingredient**	**Source**	**5%S**	**5%S +15%G**	**5%S +15%F**
Sucrose (5% w/v)	Tate & Lyle, Granulated sugar	50 g		
D-Glucose (15% w/v)	Sigma G8270	-	150 g	-
D-Fructose (15% w/v)	Sigma F0127	-	-	150 g
Yeast (10% w/v)	MP Biomedicals, #903312	100 g		
Agar (1.5% w/v)	Sigma, A7002	15 g		
Nipagin	Sigma, H5501	30 mL of 10% w/v nipagin in 95% EtOH		
Propionic acid (0.3% v/v)	Sigma, P1386	3 mL		
H_2O		up to 1 L		

Allopurinol (AP) Treatment (per L of Media)

		5%S ±AP				20%S ±AP			
**Ingredient**	**Source**	-	+10 μM	+100 μM	+1 mM	-	+10 μM	+100 μM	+1 mM
Sucrose (% w/v)	Tate & Lyle, Granulated sugar	50 g				200 g			
Yeast (10% w/v)	MP Biomedicals, #903312	100 g							
Agar (1.5% w/v)	Sigma, A7002	15 g							
Nipagin	Sigma, H5501	30 mL of 10% w/v nipagin in 95% EtOH							
Propionic acid (0.3% v/v)	Sigma, P1386	3 mL							
Allopurinol	Sigma, A8003	-	1.361 mg	13.61 mg	136.11 mg	-	1.361 mg	13.61 mg	136.11 mg
H_2O		up to 1 L							

High Purine Diet (per L of Media)

**Ingredient**	**Source**	**5%S**	**5%S +10 mM purine**
Sucrose (% w/v)	Tate & Lyle, Granulated sugar	50 g	
Yeast (10% w/v)	MP Biomedicals, #903312	100 g	
Agar (1.5% w/v)	Sigma, A7002	15 g	
Nipagin	Sigma, H5501	30 mL of 10% w/v nipagin in 95% EtOH	
Propionic acid (0.3% v/v)	Sigma, P1386	3 mL	
Adenine (5 mM)	Sigma, A8626	-	675.65 mg
Guanine (5 mM)	Sigma, G11950	-	755.65 mg
H_2O		up to 1 L	

Table S2. *Drosophila* Survival Data (Related to Figures 1, 3, 4, 6)

Fig. 1E

Experiment	Genotype	Condition	Flies per vial	# vials	Total flies (set-up)	Deaths	Censors	Total flies (actual)	Median (d)
Lifespan	w^{Dah} females	5%S –H_2O	15	10	150	129	9	138	66.3
		5%S +H_2O	15	10	150	110	32	142	66.4
		20%S –H_2O	15	10	150	141	7	148	51.0
		20%S +H_2O	15	10	150	137	5	142	66.0

P-value (Log-Rank test)

	b	*c*	*d*
a	0.9347	4.4E-37	0.0585
b		3.8E-34	0.0689
c			5.5E-31
d			

Fig. 1F

Experiment	Genotype	Condition	Flies per vial	# vials	Total flies (set-up)	Deaths	Censors	Total flies (actual)	Median (d)
Lifespan	w^{1118} females	5%S –H_2O	15	11	165	157	9	166	75.2
		5%S +H_2O	15	11	165	152	15	167	75.5
		20%S –H_2O	15	11	165	162	4	166	59.5
		20%S +H_2O	15	11	165	161	3	164	76.9

P-value (Log-Rank test)

	b	*c*	*d*
a	0.6184	3.1E-59	0.1403
b		4.6E-58	0.0345
c			1.9E-63
d			

Fig. 1G

Experiment	Genotype	Condition	Flies per vial	# vials	Total flies (set-up)	Deaths	Censors	Total flies (actual)	Median (d)
Lifespan (D-fructose)	w^{Dah} females	5%S –H_2O	15	10	150	145	2	147	68.1
		5%S +H_2O	15	10	150	142	8	150	71.3
		5%S+15% D-F –H_2O	15	10	150	144	2	146	49.4
		5%S+15% D-F +H_2O	15	10	150	132	14	146	69.2

P-value (Log-Rank test)

	b	*c*	*d*
a	0.2099	1.3E-51	0.7720
b		5.8E-52	0.2468
c			6.3E-35
d			

Fig. 1H

Experiment	Genotype	Condition	Flies per vial	# vials	Total flies (set-up)	Deaths	Censors	Total flies (actual)	Median (d)
Lifespan	w^{Dah} females	5%S –H_2O	15	9	135	130	6	136	62.7
		5%S +H_2O	15	9	135	123	11	134	63.7
		30%S –H_2O	15	8	120	113	3	116	42.6
		30%S +H_2O	15	8	120	118	0	118	60.7
		40%S –H_2O	15	8	120	114	2	116	26.1
		40%S +H_2O	15	8	120	115	3	118	60.2

P-value (Log-Rank test)

	b	*c*	*d*	*e*	*f*
a	0.6042	2.5E-37	0.0130	1.0E-49	0.0004
b		5.1E-38	0.0014	1.6E-51	2.2E-05
c			2.3E-38	2.5E-11	1.7E-28
d				9.1E-55	0.1575
e					7.3E-47
f					

Fig. 3A

Experiment	Genotype	Condition	Flies per vial	# vials	Total flies (set-up)	Deaths	Censors	Total flies (actual)	Median (d)
Lifespan	Δ*foxo* females	5%S –H_2O	15	13	195	176	15	191	39.5
		5%S +H_2O	15	13	195	173	20	193	45.7
		20%S –H_2O	15	13	195	180	14	194	26.7
		20%S +H_2O	15	13	195	185	6	191	38.0

P-value (Log-Rank test)

	b	*c*	*d*
a	6.0E-07	2.8E-21	0.0329
b		2.4E-40	5.9E-13
c			5.9E-17
d			

Fig. 3D

Experiment	Genotype	Condition	Flies per vial	# vials	Total flies (set-up)	Deaths	Censors	Total flies (actual)	Median (d)
Lifespan	Δ*foxo* *females*	2.5%S –H_2O	15	10	150	146	4	150	49.4
		2.5%S +H_2O	15	10	150	136	9	145	46.9

P-value (Log-Rank test)

	b
a	0.4767
b	

Fig. 4A

Experiment	Genotype	Condition (pre-treatment)	Flies per vial	# vials	Total flies (set-up)	Deaths	Censors	Total flies (actual)	Median (d)
Desiccation stress	w^{Dah} females d28	5%S –H_2O	15	7	105	95	0	95	0.446
		5%S +H_2O	15	7	105	103	0	103	0.418
		20%S –H_2O	15	7	105	95	0	95	0.350
		20%S +H_2O	15	7	105	94	0	94	0.417

P-value (Log-Rank test)

	b	*c*	*d*
a	0.0784	1.4E-09	0.1037
b		8.5E-07	0.7036
c			3.3E-05
d			

Fig. 4B

Experiment	Genotype	Condition (pre-treatment)	Flies per vial	# vials	Total flies (set-up)	Deaths	Censors	Total flies (actual)	Median (d)
Salt stress (500 mM NaCl)	w^{Dah} females d7	5%S –H_2O	20	6	120	115	0	115	5.1
		5%S +H_2O	20	6	120	113	0	113	5.0
		20%S –H_2O	20	5	100	96	0	96	1.9
		20%S +H_2O	20	6	120	118	0	118	2.7

P-value (Log-Rank test)

	b	*c*	*d*
a	0.5093	1.8E-27	6.5E-13
b		3.5E-31	1.9E-12
c			3.7E-08
d			

Fig. 6C

Experiment	Genotype	Condition	Flies per vial	# vials	Total flies (set-up)	Deaths	Censors	Total flies (actual)	Median (d)
Lifespan (100 μM allopurinol)	w^{Dah} females	5%S –AP	15	10	150	140	6	146	64.7
		5%S +AP	15	10	150	147	0	147	61.0
		20%S –AP	15	10	150	149	0	149	47.5
		20%S +AP	15	10	150	145	3	148	51.4

P-value (Log-Rank test)

	b	*c*	*d*
a	1.4E-04	2.7E-40	5.3E-24
b		7.0E-30	5.3E-13
c			5.7E-06
d			

Fig. 6F

Experiment	Genotype	Condition	Flies per vial	# vials	Total flies (set-up)	Deaths	Censors	Total flies (actual)	Median (d)
Lifespan (10 mM purine)	w^{Dah} females	5%S –H_2O	15	8	120	118	1	119	72.9
		5%S +H_2O	15	8	120	115	3	118	75.0
		5%S+purine –H_2O	15	8	120	120	0	120	64.3
		5%S+purine +H_2O	15	8	120	114	4	118	73.9

P-value (Log-Rank test)

	b	*c*	*d*
a	0.1633	4.5E-13	0.6151
b		6.9E-18	0.4106
c			5.8E-14
d			

Table S3. *Drosophila* Survival Data (Related to Figures 1, 3, 4, 6 and S1, S3, S4, S6)

Fig. S1F

Experiment	Genotype	Condition	Flies per vial	# vials	Total flies (set-up)	Deaths	Censors	Total flies (actual)	Median (d)
Lifespan	w^{Dah} Wolbachia negative females	5%S –H_2O	15	10	150	144	4	148	62.8
		5%S +H_2O	15	10	150	127	14	141	63.3
		20%S –H_2O	15	10	150	145	2	147	47.0
		20%S +H_2O	15	10	150	129	8	137	65.2

P-value (Log-Rank test)

	b	*c*	*d*
a	0.3795	8.0E-36	0.1177
b		1.1E-40	0.0827
c			4.4E-43
d			

Fig. S1G

Experiment	Genotype	Condition	Flies per vial	# vials	Total flies (set-up)	Deaths	Censors	Total flies (actual)	Median (d)
Lifespan	*Dahomey* females	5%S –H_2O	15	7	105	92	0	92	72.8
		5%S +H_2O	15	7	105	94	3	97	73.0
		20%S –H_2O	15	6	90	84	1	85	56.3
		20%S +H_2O	15	7	105	98	0	98	71.2

P-value (Log-Rank test)

	b	*c*	*d*
a	0.7744	2.1E-14	0.18286
b		1.8E-14	0.12491
c			7.4E-16
d			

Fig. S1H

Experiment	Genotype	Condition	Flies per vial	# vials	Total flies (set-up)	Deaths	Censors	Total flies (actual)	Median (d)
Lifespan	w^{1118} males	5%S –H_2O	20	10	200	185	13	198	78.3
		5%S +H_2O	20	10	200	179	12	191	79.3
		20%S –H_2O	20	10	200	198	2	200	66.7
		20%S +H_2O	20	10	200	187	9	196	79.1

P-value (Log-Rank test)

	b	*c*	*d*
a	0.0133	5.8E-48	0.1633
b		1.1E-45	0.1988
c			2.9E-48
d			

Fig. S1I

Experiment	Genotype	Condition	Flies per vial	# vials	Total flies (set-up)	Deaths	Censors	Total flies (actual)	Median (d)
Lifespan	ovo^{D1} females	5%S –H_2O	20	10	200	193	6	199	67.6
		5%S +H_2O	20	10	200	193	4	197	68.3
		20%S –H_2O	20	10	200	197	1	198	52.6
		20%S +H_2O	20	10	200	193	2	195	70.0

P-value (Log-Rank test)

	b	*c*	*d*
a	0.0531	5.3E-58	0.0109
b		2.3E-64	0.5894
c			4.7E-65
d			

Fig. S1K

Experiment	Genotype	Condition	Flies per vial	# vials	Total flies (set-up)	Deaths	Censors	Total flies (actual)	Median (d)
Lifespan (D-glucose)	w^{Dah} females	5%S –H_2O	15	10	150	136	3	139	66.5
		5%S +H_2O	15	10	150	146	1	147	67.8
		5%S+15% D-G –H_2O	15	10	150	145	4	149	53.0
		5%S+15% D-G +H_2O	15	10	150	143	5	148	65.4

P-value (Log-Rank test)

	b	*c*	*d*
a	0.4259	1.3E-25	0.9382
b		3.6E-28	0.4137
c			5.5E-23
d			

Fig. S3A

Experiment	Genotype	Condition	Flies per vial	# vials	Total flies (set-up)	Deaths	Censors	Total flies (actual)	Median (d)
Lifespan	w^{Dah} females	5%S –H_2O	15	9	135	130	6	136	62.7
		5%S +H_2O	15	9	135	123	11	134	63.7
		20%S –H_2O	15	10	150	153	1	154	44.5
		20%S +H_2O	15	10	150	144	5	149	59.0
Lifespan	Δ*foxo* females	5%S –H_2O	15	9	135	130	6	136	38.1
		5%S +H_2O	15	9	135	121	10	131	45.6
		20%S –H_2O	15	9	135	117	21	138	27.1
		20%S +H_2O	15	9	135	124	13	137	35.4

P-value (Log-Rank test)

	b	*c*	*d*
a	0.6042	1.1E-29	0.001
b		3.9E-31	6.6E-05
c			6.9E-24
d			

	f	*g*	*h*
e	2.0E-08	3.2E-14	0.8824
f		2.5E-31	7.6E-09
g			2.1E-13
h			

Fig. S3D

Experiment	Genotype	Condition	Flies per vial	# vials	Total flies (set-up)	Deaths	Censors	Total flies (actual)	Median (d)
Lifespan	Δ*foxo* females	0%S –H_2O	15	10	150	149	0	149	40.2
		0%S +H_2O	15	10	150	147	3	150	37.9
		1%S –H_2O	15	10	150	146	2	148	44.7
		1%S +H_2O	15	10	150	151	1	152	45.7

P-value (Log-Rank test)

	b	*c*	*d*
a	0.3017	1.0E-03	8.2E-06
b		1.5E-05	3.5E-08
c			0.2079
d			

Fig. S4B

Experiment	Genotype	Condition (pre-treatment)	Flies per vial	# vials	Total flies (set-up)	Deaths	Censors	Total flies (actual)	Median (d)
Oxidative stress (20 mM paraquat)	w^{Dah} females d7	5%S –H_2O	20	8	160	158	0	158	1.29
		5%S +H_2O	20	8	160	161	0	161	1.22
		20%S –H_2O	20	8	160	159	0	159	1.30
		20%S +H_2O	20	8	160	160	0	160	1.25

P-value (Log-Rank test)

	b	*c*	*d*
a	0.1702	0.6081	0.4543
b		0.0578	0.5040
c			0.2292
d			

Fig. S4C

Experiment	Genotype	Condition (pre-treatment)	Flies per vial	# vials	Total flies (set-up)	Deaths	Censors	Total flies (actual)	Median (d)
Starvation stress (1.5% agar)	w^{Dah} females d7	5%S –H_2O	15	10	150	146	0	146	10.2
		5%S +H_2O	15	10	150	132	0	132	10.2
		20%S –H_2O	15	10	150	143	0	143	11.4
		20%S +H_2O	15	10	150	145	0	145	11.7

P-value (Log-Rank test)

	b	*c*	*d*
a	0.8907	4.3E-13	3.2E-17
b		3.4E-11	8.5E-15
c			0.1624
d			

Table S3. *Drosophila* Survival Data (Related to Figures 1, 3, 4, 6 and S1, S3, S4, S6) - *continued*

Fig. S6A

Experiment	Genotype	Condition	Flies per vial	# vials	Total flies (set-up)	Deaths	Censors	Total flies (actual)	Median (d)
Lifespan (1 mM allopurinol)	w^{Dah} females	5%S –AP	15	10	150	146	1	147	68.4
		5%S +AP	15	10	150	145	1	146	58.2
		20%S –AP	15	10	150	143	6	149	56.3
		20%S +AP	15	9	135	133	2	135	46.9

P-value (Log-Rank test)

	b	*c*	*d*
a	4.8E-19	2.4E-28	1.5E-55
b		0.0044	7.5E-41
c			9.7E-27
d			

Fig. S6F
Fig. S6I

Experiment	Genotype	Condition	Flies per vial	# vials	Total flies (set-up)	Deaths	Censors	Total flies (actual)	Median (d)
Lifespan (10 and 100 µM allopurinol)	w^{Dah} females	5%S –AP	15	10	150	143	5	148	63.6
		5%S +AP (10 µM)	15	10	150	149	1	150	63.2
		5%S +AP (100 µM)	15	10	150	148	1	149	61.2
		20%S –AP	15	10	150	148	0	148	46.7
		20%S +AP (10 µM)	15	10	150	139	9	148	51.5
		20%S +AP (100 µM)	15	10	150	144	2	146	49.7

P-value (Log-Rank test)

	b	*c*	*d*	*e*	*f*
a	0.2670	1.2E-05	1.4E-53	4.4E-33	1.2E-28
b		5.7E-04	5.0E-48	8.3E-29	4.5E-25
c			1.5E-36	4.1E-18	3.2E-15
d				3.0E-08	2.5E-06
e					0.9977
f					

Fig. S6J

Experiment	Genotype	Condition	Flies per vial	# vials	Total flies (set-up)	Deaths	Censors	Total flies (actual)	Median (d)
Lifespan (10 µM allopurinol)	w^{Dah} females	5%S –AP	15	10	150	146	1	147	68.4
		5%S +AP	15	10	150	149	1	150	68.3
		30%S –AP	15	10	150	148	2	150	44.7
		30%S +AP	15	10	150	144	3	147	48.1

P-value (Log-Rank test)

	b	*c*	*d*
a	0.8028	1.9E-59	5.3E-52
b		3.7E-65	3.0E-57
c			5.0E-06
d			

Fig. S6K

Experiment	Genotype	Condition	Flies per vial	# vials	Total flies (set-up)	Deaths	Censors	Total flies (actual)	Median (d)
Lifespan (10 mM purine)	w^{Dah} females	5%S –H_2O	15	9	135	122	12	134	65.9
		5%S +H_2O	15	9	135	113	18	131	68.1
		5%S+purine –H_2O	15	10	150	148	1	149	59.4
		5%S+purine +H_2O	15	10	150	132	17	149	69.2

P-value (Log-Rank test)

	b	*c*	*d*
a	0.2716	3.3E-14	0.0856
b		1.2E-17	0.5854
c			3.6E-21
d			

Table S4. Compounds Analysed by HILIC UPLC-HRMS Metabolomics in the *Drosophila* Rectal Ampulla Stones (Related to STAR Methods)

Compound	Mass (m/z)	Retention Time (min)	Formula	Polarity
Allopurinol	135.03123	2.06	$C_5H_4N_4O$	Negative
$^{13}C_5$-Hypoxanthine	140.04801	3.16	$[^{13}C]_5H_4N_4O$	Negative
Hypoxanthine	135.03123	3.16	$C_5H_4N_4O$	Negative
Xanthine	151.02615	3.90	$C_5H_4N_4O_2$	Negative
Allantoin	157.03671	3.94	$C_4H_6N_4O_3$	Negative
Uric acid	167.02106	10.98	$C_5H_4N_4O_3$	Negative

Table S5. Clinical Information on the Human Cohort (Related to STAR Methods)

	Number of Participants	Age (years)	BMI (kg/m^2)	eGFR (mg/mL per 1.73 m^2)	Blood Glucose (mg/dL)
**Total**	650	60 (51 - 69)	26.5 (24.0 - 29.2)	85 (77 - 96)	96 (91 - 102)
**Males**	367	60 (52 - 68)	26.8 (24.8 - 29.3)	86 (78 - 97)	98 (93 - 103.5)
**Females**	283	61 (51 - 70)	25.9 (22.5 - 29.1)	83 (75 - 95)	94 (89 - 100)

Age, body mass index (BMI), estimated glomerular filtration rate (eGFR), and blood glucose levels are given as median with range from lower (25%) to upper quartile (75%).
